# Hygiene practices among young adolescents aged 12-15 years in low- and middle-income countries: a population-based study

**DOI:** 10.7189/jogh.10.020436

**Published:** 2020-12

**Authors:** Liyuan Han, Xuping Gao, Minqi Liao, Xiaoxuan Yu, Ruijie Zhang, Shiwei Liu, Fangfang Zeng

**Affiliations:** 1 Hwa Mei Hospital, University of Chinese Academy of Sciences, Ningbo, Zhejiang, PR China; 2 Department of Global Health, Ningbo Institute of Life and Health Industry, University of Chinese Academy of Sciences, Ningbo, PR China; 3 Department of Child & Adolescent Psychiatry, Peking University Sixth Hospital (Institute of Mental Health), National Clinical Research Center for Mental Disorders and NHC Key Laboratory of Mental Health (Peking University Sixth Hospital), Beijing, PR China; 4 Department of Epidemiology, School of Medicine, Jinan University, Guangzhou, Guangdong, PR China; 5 National Center for Chronic and Noncommunicable Disease Control and Prevention, Chinese Center for Disease Control and Prevention, Beijing, PR China

## Abstract

**Background:**

Poor personal hygiene increases disease risk, however, the prevalence of hygiene practices among adolescents is poorly described in low- and middle-income countries (LMICs). We aimed to assess the hygiene practices among young adolescents in LMICs using data from the Global School-based Student Health Surveys (GSHS).

**Methods:**

This population-based study analysed the GSHS data for adolescents aged 12-15 years from 75 LMICs. Data were collected between 2003 and 2015 using standardised, anonymous, self-reported questionnaires. This report focuses on hygiene related behaviours such as tooth brushing, washing hands after using the toilet, washing hands before eating and washing hands with soap. The weighted prevalence and 95% confidential intervals (CIs) for the hygiene practices, and overall and regional estimates were calculated with random-effects meta-analysis.

**Results:**

A total of 181 848 young adolescents from 75 LIMCs with available hygiene data were analysed. The overall prevalence for never washing hands were 7.4% (95% confidence interval (CI) = 4.4-10.3) for before eating, 5.9% (95% CI = 3.8-7.9) for after using the toilet, and 9.0% (95% CI = 6.2-11.8) for with soap. For tooth brushing, the overall prevalence estimates were 8.6% (95% CI = 5.5-11.7) for never brushing teeth, 80.9% (95% CI = 74.7-87.1) for 1-3 times per day, and 9.7% (95% CI = 5.8-13.6) for >3 times per day. However, the prevalence of different hygiene practices varied significantly among countries and regions (all *P* values <0.001). Poor hygiene status, with a prevalence >20%, was still observed in several LMICs (eg, 12 countries for never washing hands and 4 for never brushing teeth).

**Conclusions:**

The hygiene practices of young adolescents aged 12-15 years were generally frequent, but poor status was still observed in several LMICs. These findings emphases the need for hygiene and health education targeting young adolescents in LMICs.

Non-fatal health problems with childhood onset profoundly affect long-term health trajectories, future health care needs, intellectual development and economic and productivity prospects [1]. In 2015, there were approximately 7.26 million deaths among children and adolescents globally, and high mortality mainly found in low- and middle-income countries (LMICs), especially in South Asia, Western sub-Saharan Africa, and Eastern sub-Saharan Africa [1]. Hygiene practices such as hand washing and tooth brushing in LMICs have received comparatively little attention, despite the fact that inadequate sanitation and poor personal hygiene conditions in these countries profoundly contribute to the spread and incidence of diseases (especially gastrointestinal and respiratory illnesses) [2-5]. Study by Allison et al [5] found that improvements in hand hygiene resulted in reductions in gastrointestinal illness of 31% (95% confidence intervals (CI) = 19%, 42%) and reductions in respiratory illness of 21% (95% CI = 5%, 34%).

As a cost-effective hygienic habit, hand hygiene is the primary measure to reduce childhood diarrhoea and respiratory infections, which are the leading causes of infection-related death among children and adolescents, with age-standardised mortality rates of 31.1 and 22.4 per 100 000 global population [1]. Person to person contact or by ingestion of contaminated food and water in an unhygienic environment are mostly transmitted pathways for these diseases [4,6]. Hand washing has been proven to reduce the risk of infections associated with childhood diarrhoea and respiratory diseases by 29%-31% and 16%-24%, respectively [2,3]. However, in many resource-poor countries, developing a habit of hand washing may require infrastructural, cultural and behavioural changes, which take time to develop, as well as substantial resources [7,8].

Oral hygiene is also a low-cost but effective hygiene practice that can decrease the incidence of oral diseases, such as periodontal disease and dental caries [9,10]. Tooth brushing with fluoride-containing toothpaste has been suggested as an effective way to prevent dental caries, and reduce caries risk by 24% in permanent teeth [11,12]. The Global Burden of Disease Study 2016 estimated that oral diseases affected half of the global population (3.58 billion people), and dental caries is the most common oral disease among children which affects 60%-90% of children worldwide [13]. With increasing urbanisation and changes in living conditions, the prevalence of oral diseases has increased notably in several high-income countries, whereas in LMICs, the persistence of the disease burden is likely to be due to inadequate exposure to fluoride and poor access to primary oral health care services [14].

Understanding the distribution of hygiene practices among adolescents in different LMICs is of utmost importance for health and other youth-centric services (eg,, education), evidence-based planning, priority setting and disease prevention and intervention efforts. This study aimed to assess the pattern of hand washing and tooth brushing among adolescents aged 12-15 years in LMICs using the latest data from the Global School-based Student Health Surveys (GSHS).

## METHODS

### Data sources

We used the most recent GSHS data (2003-2015) publicly available on the websites of the WHO (http://www.who.int/ncds/surveillance/gshs/en/) and the US Centers for Disease Control and Prevention (CDC) (https://www.cdc.gov/gshs/index.htm). Detailed methods and the main findings of the GSHS are described on both websites, as well as in previous studies [15,16]. The GSHS is designed to help countries measure and assess behavioural risk factors and protective factors among young people. The GSHS uses the same two-stage random cluster sampling of schools and classes to select eligible participants in all countries, which provides a sample representative of the young population in each country. For global comparisons, we used hygiene module data collected from young adolescents aged 12-15 years using self-administered and well-validated questionnaire. If a country had done more than one GSHS between 2003 and 2015, we used data from the most recent survey.

In each participating country, the GSHS survey has been approved by both a national government administration (most often the Ministry of Health or Education) and an institutional review board or ethics committee. Student participants indicate their consent to participate by voluntarily completing an anonymous survey form.

### Outcomes

The outcomes in our study are frequencies of young adolescents’ hygiene practices of hand washing (after using the toilet, before eating and with soap) and tooth brushing.

The frequency of hand washing was assessed among young adolescents using the following three questions: ‘During the past 30 days, how often did you wash your hands before eating?’; ‘During the past 30 days, how often did you wash your hands after using the toilet or latrine?’; and ‘During the past 30 days, how often did you use soap when washing your hands?’. The possible answers were ‘never’, ‘rarely’, ‘sometimes’, ‘most of the time’, or ‘always’.

Tooth brushing frequency was assessed with the question: ‘During the past 30 days, how many times per day did you usually clean or brush your teeth?’. The possible answers were ‘I did not clean or brush my teeth during the past 30 days’, ‘Less than 1 time per day’, ‘1 time per day’, ‘2 times per day’, ‘3 times per day’, or ‘4 or more times per day’.

For the questions about washing hands before eating, after using the toilet or with soap, the responses ‘sometimes’, ‘most of the time’ and ‘always’ were coded as frequent hand washing; other responses (‘never’ or ‘rarely’) were coded as never washing hands. For tooth brushing, responses were coded as never brushing teeth (for ‘did not brush’ or ‘less than 1 time per day’), 1-3 times per day, and >3 times per day.

### Statistical analysis

Estimates of the prevalence of different variables were based on individual data from each survey. To take account of the complex sampling design used for the GSHS, we calculated prevalence estimates and 95% confidence intervals (95% CIs) using the SURVEYMEANS procedure in SAS version 9.4 (SAS Institute, Cary, NC). Pooled regional and overall estimates with 95% CIs was calculated using meta-analysis with random-effects models by STATA version 11.0 (Stata Corporation, TX, USA). Heterogeneity was assessed using the I^2^ statistic. Subgroup analyses were stratified by sex, age (12-13 years vs 14-15 years) and body mass index (BMI; underweight, normal weight, overweight or obese). Age- and sex-specific BMI percentiles were calculated according to the US CDC guidelines using growth reference data from 2000 [17]. For classification of BMI categories, the cut-off values used were <5% for underweight, 5% to 85% for normal weight, 85% to 95% for overweight and >95% for obese. The differences between two prevalence estimates were compared using the χ^2^ test of heterogeneity. Survey-weighted logistic regression models were used to analyze the trends in prevalence over time with adjustments for age, sex. Statistical significance was set as a *P*-value <0.05 in a two-sided test.

The study was conducted according to STROBE checklists (www.strobe-statement.org/index.php?id=strobe-home) guidelines (Table S1 in the **Online Supplementary Document**).

## RESULTS

### Population characteristics

**Figure 1:**
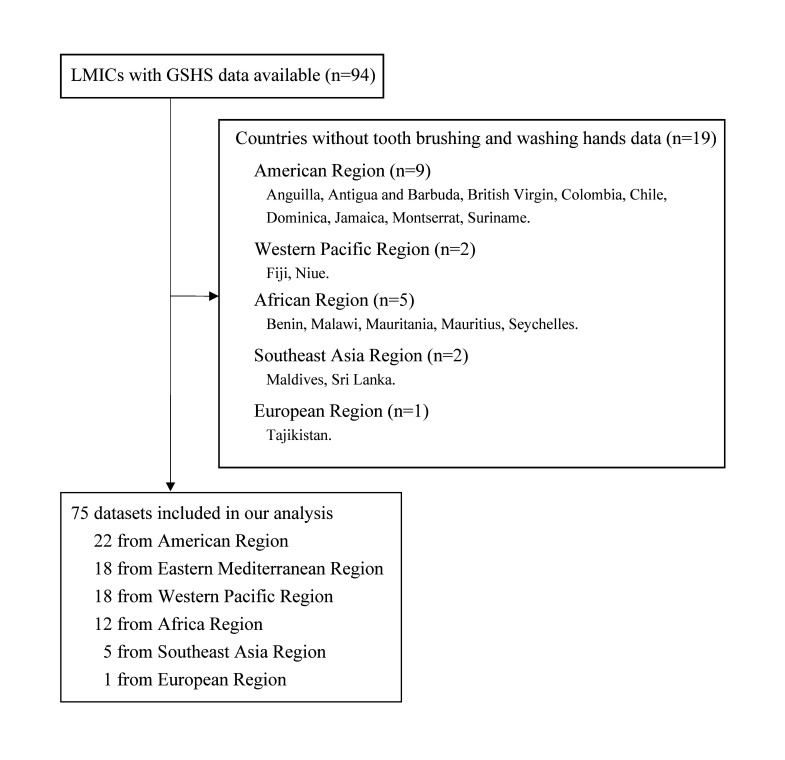
Flow of participants through the study.

Until now, 94 countries had conducted at least one GSHS. Nineteen countries were not included in our analysis due to a lack of data from the hygiene practices module (**Figure 1**). GSHS data from 75 countries in the 6 WHO regions were included: 12 from Africa, 1 from Europe, 22 from America, 18 from the eastern Mediterranean, 5 from Southeast Asia, and 18 from the western Pacific, corresponding to a total of 181 848 young adolescents (**Table 1**). Almost all of the young adolescents surveyed responded to the hygiene practice questions regarding hand washing and tooth brushing, with an overall response rate of 98.7% (range: 95.5% to 99.9%). The median sample size for each survey was 1816.

**Table 1:** Survey characteristics of the Global School-based Student Health Surveys, 2003-2015

	Survey (year)	n/N*	Response rate (%)†	Boys (%)
**Africa Region**				
Algeria	2011	3436/3471	99.0	45.6
Botswana	2005	1369/1397	98.0	42.0
Ghana	2007	4200/4248	98.9	48.7
Kenya	2003	2863/2908	98.5	45.9
Mozambique	2015	636/650	97.8	49.4
Namibia	2013	1883/1928	97.7	41.4
Senegal	2005	2613/2654	98.5	54.5
Swaziland	2013	1297/1314	98.7	38.1
Tanzania	2014	2543/2570	98.9	44.2
Uganda	2003	1816/1890	96.1	46.9
Zambia	2004	1299/1334	97.4	43.2
Zimbabwe	2003	3867/3883	99.6	40.5
**European Region:**				
Macedonia	2007	1528/1545	98.9	49.0
**American Region:**				
Argentina	2012	21177/21363	99.1	46.3
Barbados	2011	1485/1500	99.0	46.3
Belize	2011	1561/1590	98.2	46.7
Bolivia	2012	2752/2786	98.8	48.9
Cayman	2007	1135/1144	99.2	48.5
Bahamas	2013	1265/1303	97.1	45.8
Costa Rica	2009	2235/2259	98.9	47.3
Curaçao	2015	1472/1487	99.0	46.6
Ecuador	2007	4438/4515	98.3	47.7
El Salvador	2013	1595/1607	99.3	52.0
Grenada	2008	1255/1298	96.7	42.6
Guatemala	2015	3483/3560	97.8	47.5
Guyana	2010	1949/1969	99.0	44.1
Honduras	2012	1455/1482	98.2	47.0
Peru	2010	2328/2357	98.8	48.1
Saint Kitts and Nevis	2011	1446/1470	98.4	43.2
Saint Lucia	2007	1058/1070	98.9	41.7
Saint Vincent and the Grenadines	2007	1154/1182	97.6	45.5
Trinidad and Tobago	2011	2289/2347	97.5	54.3
Uruguay	2012	2810/2857	98.4	47.0
Venezuela	2003	3919/3922	99.9	44.0
**Eastern Mediterranean Region**				
Afghanistan	2014	1372/1412	97.2	36.2
Djibouti	2007	947/953	99.4	57.8
Egypt	2011	2241/2300	97.4	45.1
Iraq	2012	1488/1518	98.0	55.0
Jordan	2007	1598/1641	97.4	55.9
Kuwait	2015	1969/2014	97.8	44.8
Lebanon	2011	1945/1973	98.6	46.6
Libya	2007	1831/1862	98.3	41.8
Morocco	2010	2338/2385	98.0	49.6
Oman	2015	1611/1668	96.6	43.6
Pakistan	2011	4916/4975	98.8	74.7
Qatar	2011	1630/1707	95.5	43.9
Sudan	2012	1339/1378	97.2	35.6
Syrian Arab Republic	2010	2862/2901	98.7	40.0
Tunisia	2008	2502/2538	98.6	47.3
United Arab Emirates	2010	2268/2288	99.1	38.7
UNRWA	2010	9356/9395	99.6	44.9
Yemen	2008	852/874	97.5	57.9
**Southeast Asia Region**				
Bangladesh	2014	2711/2748	98.7	38.2
India	2007	7215/7310	98.7	54.4
Indonesia	2015	8717/8788	99.2	46.1
Thailand	2015	4088/4120	99.2	46.6
Timor-Leste	2015	1599/1613	99.1	39.8
**Western Pacific Region**				
Brunei Darussalam	2014	1809/1818	99.5	46.5
Cambodia	2013	1799/1812	99.3	43.5
China	2003	8328/8423	98.9	48.3
Cook	2015	361/364	99.2	47.5
Kiribati	2011	1321/1333	99.1	41.6
Laos	2015	1628/1639	99.3	41.5
Malaysia	2012	16189/16248	99.6	50.9
Mongolia	2013	3672/3699	99.3	47.3
Nauru	2011	349/352	99.1	42.6
Philippines	2015	6087/6155	98.9	43.4
Samoa	2011	2091/2169	96.4	38.7
Solomon	2011	901/919	98.0	48.6
Tokelau	2014	83/85	97.6	52.9
Tonga	2010	1892/1934	97.8	44.6
Tuvalu	2013	662/673	98.4	48.4
Vanuatu	2011	833/847	98.3	41.0
Vietnam	2013	1733/1740	99.6	46.4
Wallis and Futuna	2015	712/713	99.9	48.5
**All:**				
Total	-	181848/184265	98.7	46.9

*n refers to number of participants included in our analysis and N refers to the number of participants included in the GSHS.

†Response rate is for the first three health habit questions. Data are for participants aged 12-15 y.

### Hand hygiene among young adolescents in LMICs

**Figure 2:**
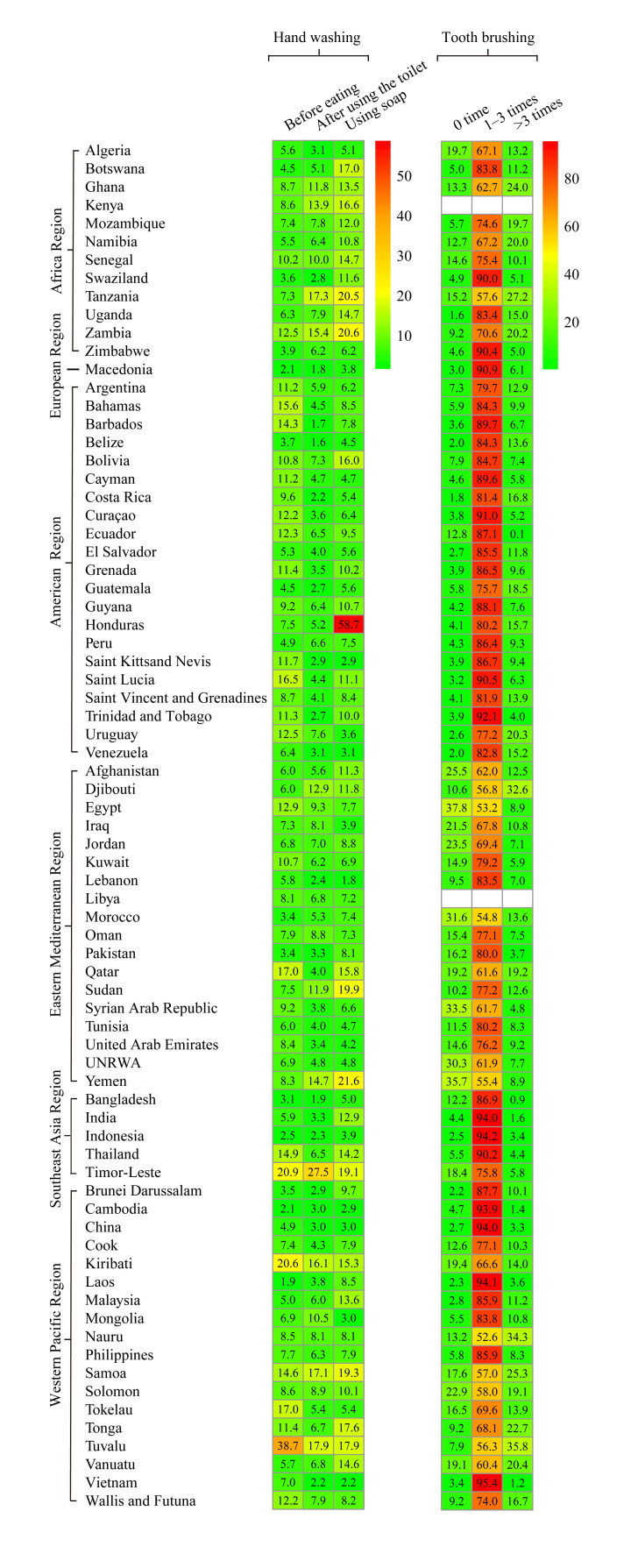
Prevalence of hand washing and tooth brushing in adolescents aged 12-15 years among 75 low-income and middle-income countries, 2003-2015.

**Figure 3:**
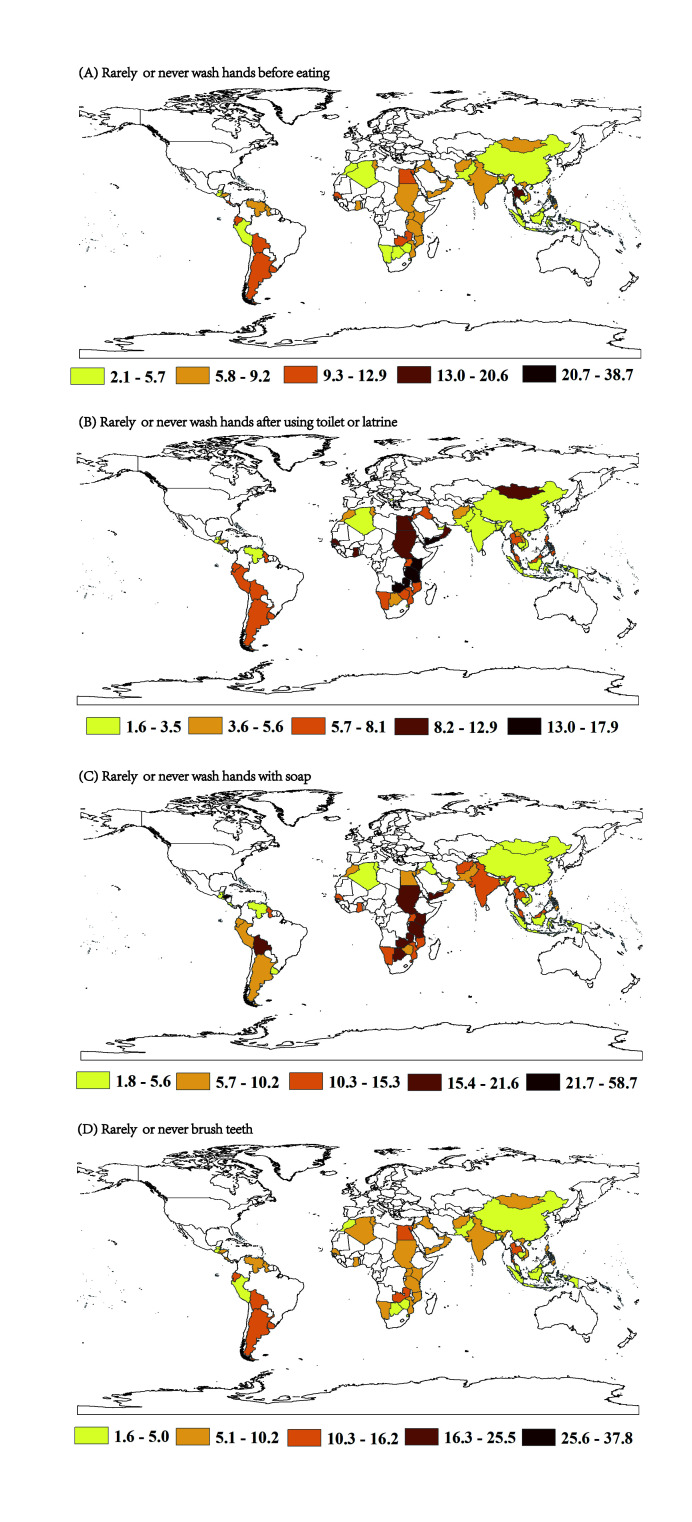
Prevalence of hand washing and tooth brushing in adolescents aged 12-15 years by countries, 2003-2015. **Panel A.** Rarely or never wash hands before eating. **Panel B.** Rarely or never wash hands after using toilet or latrine. **Panel C.** Rarely or never wash hands with soap. **Panel D.** Rarely or never brush teeth.

Overall prevalence was 7.4% (95% CI = 4.4-10.3) for never washing hands before eating, 5.9% (95% CI = 3.8-7.9) for never washing hands after using the toilet, and 9.0% (95% CI = 6.2-11.8) for never washing hands with soap (**Figure 2**; Table S2-4 in the **Online Supplementary Document**). The prevalence significantly varied among regions (all *P*-values for heterogeneity <0.001). For all types of hand washing behaviours, the European region (which included only one country, Macedonia) had the lowest prevalence of never washing hands, with prevalence of 2.1% for never washing hands both before eating and after using the toilet, and 3.8% for never washing hands with soap. The region with the highest prevalence of never washing hands before eating was America (10%, 95% CI = 8.3%-11.6%), and Africa had the highest prevalence of never washing hands after using the toilet (8.7%, 95% CI = 6.5%-10.9%) and never washing hands with soap (13.5%, 95% CI = 10.2%-16.8%). As shown in **Figure 3**, the countries with the highest and lowest prevalence of never washing hands before eating were Tuvalu (38.7%) and Laos (1.9%). Timor-Leste and Belize had the highest and lowest prevalence for never washing hands after using the toilet (27.5% and 1.6%, respectively) and Honduras and Lebanon had the highest and lowest prevalence for never washing hands with soap (58.7% and 1.8%, respectively).

### Oral hygiene among young adolescents in LMICs

For tooth brushing, the overall prevalence estimates were 8.6% (95% CI = 5.5-11.7) for never brushing teeth, 80.9% (95% CI = 74.7-87.1) for 1-3 times per day, and 9.7% (95% CI = 5.8-13.6) for >3 times per day. Significant differences in tooth brushing were found among the six WHO regions (all *P*-values <.001). The European region had the highest prevalence of daily tooth brushing (90.9%), and the Eastern Mediterranean region had the lowest (68.2%). At the country level, the highest and the lowest prevalence for daily tooth brushing were reported by students from Vietnam (95.4%) and Nauru (52.6%). In nine LMICs, mostly in the Eastern Mediterranean region, over 20% of students reported brushing their teeth less than once per day.

### Stratified analyses

**Figure 4:**
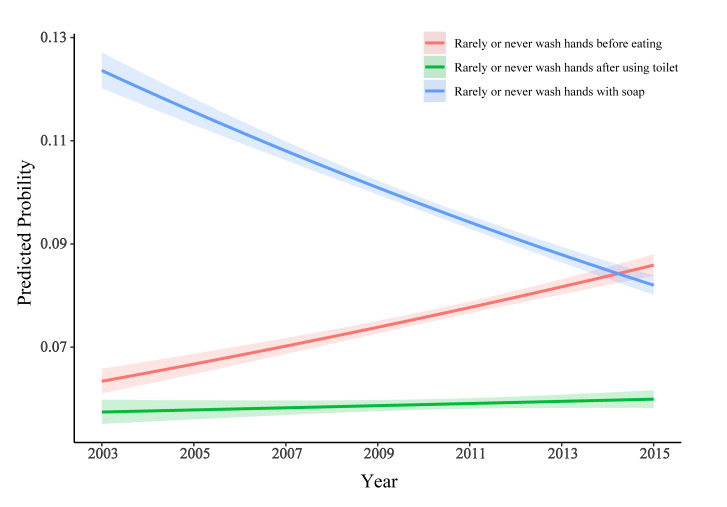
Trends of prevalence of hand washing in adolescents aged 12-15 years from 2003 to 2015.

Stratified analyses indicated that the prevalence of both never washing hands and never brushing teeth did not differ among gender, age and BMI strata (*P* range from 0.053 to 1.000; see Table S2-7 in the **Online Supplementary Document**). As shown in **Figure 4**, logistic analyses across time frames indicated significant trends of increasing prevalence for never washing hands before eating (*P* trends <0.001), and decreasing prevalence for never washing hands using soap (*P* trends <0.001).

## DISCUSSION

Although the overall prevalence of washing hands before eating, after using the toilet and with soap at least once per day, as well as daily tooth brushing, was generally high among LMICs, irrespective of age, gender or BMI (all prevalence >90%), hygiene practices were still poor in several LMICs. For example, 38.7% of students in Tuvalu never or rarely washed their hands before eating, 27.5% of students in Timor-Leste never or rarely washed their hands after using the toilet, 58.7% of students in Honduras never or rarely washed their hands with soap, and 37.8% of students in Egypt brushed their teeth less than once per day. From 2003 to 2015, washing hands with soap showed a significant increasing trend, whereas washing hands before eating showed a decreasing trend.

Currently, studies of hygiene practices among young adolescents mostly focus on oral hygiene [18-20] and at the country level [19,21-23], and the global extent and prevalence of hygiene practices (especially hand hygiene) among adolescents is poorly described. In 2015, McKittrick et al [21] reported that the prevalence of infrequent tooth brushing and hand washing among 33 174 students aged 13-15 years in 15 Latin American and Caribbean countries that participated in the GSHS ranged from 2% to 9%. A study that focused on Iran found that 67.21% of children and adolescents reported daily tooth brushing, and prevalence for washing hands before eating, after using the toilet and with soap ranged from 50.32% to 85.61% [22]. Toothbrushing frequency is similarly high among young adolescents in LIMCs and high-income countries [20], however, a meta-analysis including 42 studies suggested that frequency of handwashing with soap was about 30% higher in high-income countries comparing to LIMCs [24]. Similar to a previous study [20], our study found that estimates differed greatly among countries. The prevalence of hygiene practices varies worldwide, depending on many variables including economic status, urbanisation and parents’ education levels. Therefore, it is of utmost importance to develop health and other youth-centric services, as well as disease prevention and intervention programmes, that are tailored to different LMICs.

Despite the fact that the overall prevalence of hand washing was overall generally high, several LMICs (eg, Tuvalu, Timor-Leste, and Kiribati) showed a high prevalence of infrequent hand washing. In those LMICs, dirty latrines, a lack of toilet paper, overcrowding and the availability and accessibility of water and sanitation facilities in schools are all challenges faced by school staff trying to teach fundamental health behaviours to children [8]. Moreover, hand washing may require infrastructural, cultural and behavioural changes, which take time to develop and require substantial resources [25,26]. Children and adolescents are at risk of multiple infectious diseases when basic hygiene and hand washing habits are inadequate [4,6,27]. For example, *Shigella*, one of the common pathogens associated with childhood diarrhoea, led to 569 737 deaths of children and adolescents worldwide in 2015 [1,27,28], and there is no vaccine to prevent it [28]. However, the spread of shigellosis from an infected person to others can be stopped by frequent and careful hand washing with soap [27]. A recent meta-analysis [29] of nine community-based trials in LMICs (15 303 participants) found that promoting hand washing prevented 36% of diarrhoea cases. Frequent and careful hand washing is important among all age groups, and supervised handwashing of all children and adolescents should be followed in day care centers, schools and homes, especially in those LMICs with the highest prevalence of infrequent hand washing.

Dental caries is increasing in developing countries, and if untreated it can affect children’s quality of life [22]. In 2015, Kassebaum et al. [30] reported that around 621 million children suffered from untreated caries in deciduous teeth. Caries can alter children’s eating and sleeping habits, dietary intake and metabolic processes, and might affect school attendance, growth and weight gain [31]. Twice-daily tooth brushing with fluoride-containing toothpaste should be encouraged. Long-term exposure to an optimal level (1000 to 1500 ppm) of fluoride results in a substantially lower incidence and prevalence of tooth decay across all ages [11]. An increased frequency of daily tooth brushing was also associated with a decreased risk of tooth plaque, gingivitis and caries [32]. Thus, tooth brushing is an effective way to prevent oral diseases.

In this study, the minority (<10%) of participating students reported never brushing their teeth, which is consistent with previous studies of adolescent oral hygiene practices in LMICs [20,21]. However, a serious oral hygiene problem was also observed, less than 70% of participating students reported brushing their teeth more than once a day, which could reduce its ability to prevent oral diseases. Moreover, in eight LMICs in the Eastern Mediterranean region, over 20% of students reported brushing their teeth less than once per day. This high prevalence of infrequent brushing might be explained by the use of chewing sticks in Arab cultures, leading to a misinterpretation of the question about ‘brushing or cleaning’ teeth [20]. Based on 32 countries, Maes et al. [18] found that poor family affluence was clearly related with a low prevalence of tooth brushing. Children and adolescents in LMICs, compared to those in high-income countries, may have limited access to a variety of options for oral health promotion (eg, community water fluoridation, routine dental sealants) [33,34]. It has been documented that children and adolescents who have early-established oral health practices are more likely than others to maintain these healthy behaviours in adulthood [35,36], minimising their risks of reduced quality of life through pain and tooth loss [37], and reducing the burden of chronic diseases. Therefore, it is especially important that children and adolescents in LMICs develop good oral hygiene practices to prevent oral diseases early in life.

Global reductions in disease burden, improvements in living conditions, dietary transition and lifestyle changes make the sustainable development targets related to health in LMICs increasingly complex. The world has a larger cohort of adolescents and young people today (just under 2 billion, aged 10-24 years) than ever before, of whom 88% live in low-income and middle-income countries [38]. It is clear that improving adolescent health at the hygiene level is an essential and cost-effective investment worldwide. However, the state of knowledge of adolescent health outside high-income countries is restricted, and the information needed to develop effective interventions is commonly unavailable [39]. Currently, school oral health interventions are mostly implemented in primary schools, which is in line with the Health Promoting School concept [25]. As opportunities for school-based oral health interventions can be limited in LMICs, the establishment of prevention-oriented community health programmes is also important. For hand hygiene, the WHO suggests that everyone over 6 months of age washes their hands frequently and practices good personal hygiene during food handling and preparation activities, and notes that persons with diarrhoea, especially children, should wash their hands after using the toilet [27]. Toothbrushing is considered a prerequisite for maintaining good oral health, but some study also suggested that excessive hygiene might be harmful. For example, toothbrushing also has the potential to have an impact on tooth wear, particularly with regard to dental erosion [40]. In addition, our findings highlight the importance of understanding sustainable development goals (SDG) related to malaria, access to safe water, sanitation and hygiene.

The main strength of our study is its large and nationally representative sample of adolescents, with assessment of hygiene patterns in most countries using standardised and well-validated questionnaires [41,42]. However, several limitations should also be considered. First, the GSHS is a self-report survey administered in school settings across countries, which can be subject to recall bias and problems of understanding of the questions. Additionally, different cultural factors in LMICs can results in different patterns of hygienic practices, which can in turn affect self-reporting about prevalence of hygienic practices, a further potential bias in data across countries. In Arab cultures, ‘tooth brushing or cleaning’ may introduce ambiguity about chewing sticks being a form of tooth cleaning [20]. Second, we observed substantial heterogeneity in the prevalence of hygienic practices across regions, which were not fully explained by major study characteristics. Therefore, overall and regional estimates must be interpreted cautiously. Third, estimates are representative at the country level, but we lack additional variables to perform subanalyses by setting (urban vs rural), social economic status or health literacy education. Fourth, GSHS data was collected between a fairly long period of time (2003-2015) and direct comparison between countries should be made with caution. However, most of the surveys (54 of 75) in our study were conducted between a narrow time interval (2009-15).

## CONCLUSION

The findings of this population-based study suggest that although hygiene practices are generally high in most LMICs, they remain poor in several LMICs. Increasing trends of poor hygiene practices was also observed, which emphasises the need for hygiene and health education targeting young adolescents in LMICs.

## Additional material

Online Supplementary Document

## References

[1] Kassebaum N Kyu HH Zoeckler L Olsen HE Thomas K Pinho C Child and Adolescent Health From 1990 to 2015: Findings From the Global Burden of Diseases, Injuries, and Risk Factors 2015 Study. JAMA Pediatr. 2017;171:573-92. 10.1001/jamapediatrics.2017.0250 28384795 PMC5540012

[2] Rabie T Curtis V Handwashing and risk of respiratory infections: a quantitative systematic review. Trop Med Int Health. 2006;11:258-67. 10.1111/j.1365-3156.2006.01568.x 16553905 PMC7169664

[3] Ejemot RI Ehiri JE Meremikwu MM Critchley JA Hand washing for preventing diarrhoea. Cochrane Database Syst Rev. 2008;CD004265. 18254044 10.1002/14651858.CD004265.pub2

[4] Curtis VA Danquah LO Aunger RV Planned, motivated and habitual hygiene behaviour: an eleven country review. Health Educ Res. 2009;24:655-73. 10.1093/her/cyp002 19286894 PMC2706491

[5] Aiello AE Coulborn RM Perez V Larson EL Effect of hand hygiene on infectious disease risk in the community setting: a meta-analysis. Am J Public Health. 2008;98:1372-81. 10.2105/AJPH.2007.124610 18556606 PMC2446461

[6] Black RE Lopez de Romana G Brown KH Bravo N Bazalar OG Kanashiro HC Incidence and etiology of infantile diarrhea and major routes of transmission in Huascar, Peru. Am J Epidemiol. 1989;129:785-99. 10.1093/oxfordjournals.aje.a115193 2646919

[7] Yeager BA Huttly SR Bartolini R Rojas M Lanata CF Defecation practices of young children in a Peruvian shanty town. Soc Sci Med. 1999;49:531-41. 10.1016/S0277-9536(99)00119-7 10414812

[8] Luby S The role of handwashing in improving hygiene and health in low-income countries. Am J Infect Control. 2001;29:239-40. 10.1067/mic.2001.115678 11486262

[9] Petersen PE Bourgeois D Ogawa H Estupinan-Day S Ndiaye C The global burden of oral diseases and risks to oral health. Bull World Health Organ. 2005;83:661-9. 16211157 PMC2626328

[10] Zaborskis A Milciuviene S Narbutaite J Bendoraitiene E Kavaliauskiene A Caries experience and oral health behaviour among 11 -13-year-olds: an ecological study of data from 27 European countries, Israel, Canada and USA. Community Dent Health. 2010;27:102-8. 20648887

[11] O’Mullane DM Baez RJ Jones S Lennon MA Petersen PE Rugg-Gunn AJ Fluoride and Oral Health. Community Dent Health. 2016;33:69-99. 27352462

[12] Petersen PE Ogawa H Prevention of dental caries through the use of fluoride–the WHO approach. Community Dent Health. 2016;33:66-8. 27352461

[13] Petersen PE The World Oral Health Report 2003: continuous improvement of oral health in the 21st century–the approach of the WHO Global Oral Health Programme. Community Dent Oral Epidemiol. 2003;31 Suppl 1:3-23. 10.1046/j..2003.com122.x 15015736

[14] World Health Organization. Oral-health. Available: https://www.who.int/news-room/fact-sheets/detail/oral-health2018. Accessed: 1 March 2019.

[15] Reza Ziaei SD, Soares J, Baybordi E, Zeinalzade AH, Rahimi VA, Mohammadi R. Reliability and Validity of the Persian Version of Global School-based Student Health Survey Adapted for Iranian School Students. Journal of Clinical Research & Governance. 2014:3.

[16] Hallifax RJ Goldacre R Landray MJ Rahman NM Goldacre MJ Trends in the Incidence and Recurrence of Inpatient-Treated Spontaneous Pneumothorax, 1968-2016. JAMA. 2018;320:1471-80. 10.1001/jama.2018.14299 30304427 PMC6233798

[17] Kuczmarski RJ, Ogden CL, Guo SS, Grummer-Strawn LM, Flegal KM, Mei Z, et al. CDC Growth Charts for the United States: methods and development. Vital and health statistics Series 11. Data from the National Health Survey. 2000. 12043359

[18] Maes L Vereecken C Vanobbergen J Honkala S Tooth brushing and social characteristics of families in 32 countries. Int Dent J. 2006;56:159-67. 10.1111/j.1875-595X.2006.tb00089.x 16826883

[19] Siziya S Muula AS Rudatsikira E Self-reported poor oral hygiene among in-school adolescents in Zambia. BMC Res Notes. 2011;4:255. 10.1186/1756-0500-4-255 21781301 PMC3156756

[20] McKittrick TR Jacobsen KH Oral hygiene practices among middle-school students in 44 low- and middle-income countries. Int Dent J. 2014;64:164-70. 10.1111/idj.12094 24571228 PMC9376393

[21] McKittrick TR Jacobsen KH Oral Hygiene and Handwashing Practices among Middle School Students in 15 Latin American and Caribbean Countries. West Indian Med J. 2015;64:266-8. 26426181 10.7727/wimj.2014.128 PMC4763903

[22] Qorbani M Kelishadi R Djalalinia S Motlagh ME Kasaeian A Ardalan G Regional disparity in hygienic behaviors of Iranian children and adolescents: The CASPIAN-IV study. Med J Islam Repub Iran. 2016;30:431. 28210596 PMC5307629

[23] Pasewaldt SE, Baller SL, Blackstone SR, Bryan Malenke L. Impact of a Hand Hygiene Curriculum and Group Handwashing Station at Two Primary Schools in East Africa. Int Q Community Health Educ. 2018. 10.1177/0272684X18819968 30577725

[24] Freeman MC Stocks ME Cumming O Jeandron A Higgins JP Wolf J Hygiene and health: systematic review of handwashing practices worldwide and update of health effects. Trop Med Int Health. 2014;19:906-16. 10.1111/tmi.12339 24889816

[25] Jurgensen N Petersen PE Promoting oral health of children through schools–results from a WHO global survey 2012. Community Dent Health. 2013;30:204-18. 24575523

[26] Petersen PE Hunsrisakhun J Thearmontree A Pithpornchaiyakul S Hintao J Jurgensen N School-based intervention for improving the oral health of children in southern Thailand. Community Dent Health. 2015;32:44-50. 26263592

[27] World Health Organization. Children and Food Safety. Available: https://www.who.int/ceh/capacity/food.pdf2009. Accessed: 1 March 2019.

[28] Baker S The HC. Recent insights into Shigella. Curr Opin Infect Dis. 2018;31:449-54. 10.1097/QCO.0000000000000475 30048255 PMC6143181

[29] Ejemot-Nwadiaro RI Ehiri JE Arikpo D Meremikwu MM Critchley JA Hand washing promotion for preventing diarrhoea. Cochrane Database Syst Rev. 2015;CD004265. 26346329 10.1002/14651858.CD004265.pub3 PMC4563982

[30] Kassebaum NJ Bernabe E Dahiya M Bhandari B Murray CJ Marcenes W Global burden of untreated caries: a systematic review and metaregression. J Dent Res. 2015;94:650-8. 10.1177/0022034515573272 25740856

[31] Sheiham A Oral health, general health and quality of life. Bull World Health Organ. 2005;83:644. 16211151 PMC2626333

[32] Ainamo J Parviainen K Occurrence of plaque, gingivitis and caries as related to self reported frequency of toothbrushing in fluoride areas in Finland. Community Dent Oral Epidemiol. 1979;7:142-6. 10.1111/j.1600-0528.1979.tb01202.x 287583

[33] Jones S Burt BA Petersen PE Lennon MA The effective use of fluorides in public health. Bull World Health Organ. 2005;83:670-6. 16211158 PMC2626340

[34] Gooch BF Truman BI Griffin SO Kohn WG Sulemana I Gift HC A comparison of selected evidence reviews and recommendations on interventions to prevent dental caries, oral and pharyngeal cancers, and sports-related craniofacial injuries. Am J Prev Med. 2002;23:55-80. 10.1016/S0749-3797(02)00450-6 12091094

[35] Kuusela S Honkala E Rimpela A Toothbrushing frequency between the ages of 12 and 18 years–longitudinal prospective studies of Finnish adolescents. Community Dent Health. 1996;13:34-9. 8634895

[36] Kwan SY Petersen PE Pine CM Borutta A Health-promoting schools: an opportunity for oral health promotion. Bull World Health Organ. 2005;83:677-85. 16211159 PMC2626337

[37] Petersen PE Kwan S Equity, social determinants and public health programmes–the case of oral health. Community Dent Oral Epidemiol. 2011;39:481-7. 10.1111/j.1600-0528.2011.00623.x 21623864

[38] United Nations Population Fund. The state of the world population 2018. Available: https://www.unfpa.org/sites/default/files/pub-pdf/UNFPA_PUB_2018_EN_SWP.pdf. Accessed: 1 March 2019.

[39] Patton GC Coffey C Cappa C Currie D Riley L Gore F Health of the world’s adolescents: a synthesis of internationally comparable data. Lancet. 2012;379:1665-75. 10.1016/S0140-6736(12)60203-7 22538181

[40] Wiegand A Schlueter N The role of oral hygiene: does toothbrushing harm? Monogr Oral Sci. 2014;25:215-9. 10.1159/000360379 24993269

[41] Franchini R Petri A Migliario M Rimondini L Poor oral hygiene and gingivitis are associated with obesity and overweight status in paediatric subjects. J Clin Periodontol. 2011;38:1021-8. 10.1111/j.1600-051X.2011.01770.x 21793868

[42] Beck NI Arif I Paumier MF Jacobsen KH Adolescent injuries in Argentina, Bolivia, Chile, and Uruguay: Results from the 2012-2013 Global School-based Student Health Survey (GSHS). Injury. 2016;47:2642-9. 10.1016/j.injury.2016.10.002 27745690

