# Supplementary material for: Hygiene practices among young adolescents aged 12-15 years in low- and middle-income countries: a population-based study
Source: J Glob Health. 2020 Dec 6;10(2):020436. doi: 10.7189/jogh.10.020436 (PMC7719273; doi:10.7189/jogh.10.020436)
Supplement: Online Supplementary Document [file jogh-10-020436-s001.pdf]

Table S1 STROBE Statement—Checklist of items that should be included in reports of *cross-sectional studies*

	Item No.	Recommendation	Page No.
**Title and abstract**	1	(*a*) Indicate the study's design with a commonly used term in the title or the abstract	3
		(*b*) Provide in the abstract an informative and balanced summary of what was done and what was found	3
**Introduction**			
Background/rationale	2	Explain the scientific background and rationale for the investigation being reported	4
Objectives	3	State specific objectives, including any prespecified hypotheses	5
**Methods**			
Study design	4	Present key elements of study design early in the paper	5
Setting	5	Describe the setting, locations, and relevant dates, including periods of recruitment, exposure, follow-up, and data collection	5-6
Participants	6	(*a*) Give the eligibility criteria, and the sources and methods of selection of participants	6
Variables	7	Clearly define all outcomes, exposures, predictors, potential confounders, and effect modifiers. Give diagnostic criteria, if applicable	6-7
Data sources/ measurement	8*	For each variable of interest, give sources of data and details of methods of assessment (measurement). Describe comparability of assessment methods if there is more than one group	6-7
Bias	9	Describe any efforts to address potential sources of bias	13
NA	10	Explain how the study size was arrived at	5-6
Quantitative variables	11	Explain how quantitative variables were handled in the analyses. If applicable, describe which groupings were chosen and why	6-7
Statistical methods	12	(*a*) Describe all statistical methods, including those used to control for confounding	7
		(*b*) Describe any methods used to examine subgroups and interactions	7
		(*c*) Explain how missing data were addressed	NA
		(*d*) If applicable, describe analytical methods taking account of sampling strategy	7
		(*e*) Describe any sensitivity analyses	NA
**Results**			
Participants	13*	(a) Report numbers of individuals at each stage of study—eg numbers potentially eligible, examined for eligibility, confirmed	7

		eligible, included in the study, completing follow-up, and analysed	
		(b) Give reasons for non-participation at each stage	NA
		(c) Consider use of a flow diagram	Fig. 1
Descriptive data	14*	(a) Give characteristics of study participants (eg demographic, clinical, social) and information on exposures and potential confounders	7-8
		(b) Indicate number of participants with missing data for each variable of interest	NA
Outcome data	15*	Report numbers of outcome events or summary measures	8-9
Main results	16	(*a*) Give unadjusted estimates and, if applicable, confounder-adjusted estimates and their precision (eg, 95% confidence interval). Make clear which confounders were adjusted for and why they were included	8-9
		(*b*) Report category boundaries when continuous variables were categorized	NA
		(*c*) If relevant, consider translating estimates of relative risk into absolute risk for a meaningful time period	NA
Other analyses	17	Report other analyses done—eg analyses of subgroups and interactions, and sensitivity analyses	9
**Discussion**			
Key results	18	Summarise key results with reference to study objectives	9-10
Limitations	19	Discuss limitations of the study, taking into account sources of potential bias or imprecision. Discuss both direction and magnitude of any potential bias	13
Interpretation	20	Give a cautious overall interpretation of results considering objectives, limitations, multiplicity of analyses, results from similar studies, and other relevant evidence	10-12
Generalisability	21	Discuss the generalisability (external validity) of the study results	12-13
**Other information**			
Funding	22	Give the source of funding and the role of the funders for the present study and, if applicable, for the original study on which the present article is based	14

NA, not applicable.

*Give information separately for exposed and unexposed groups.

Note: An Explanation and Elaboration article discusses each checklist item and gives methodological background and published examples of transparent reporting. The STROBE checklist is best used in conjunction with this article (freely available on the Web sites of PLoS Medicine at http://www.plosmedicine.org/, Annals of Internal Medicine at http://www.annals.org/, and Epidemiology at http://www.epidem.com/). Information on the STROBE Initiative is available at www.strobe-statement.org.

Table S2 Prevalence of young adolescents who rarely or never wash hands before eating

	Total, % (95%CI)	Boys, % (95%CI)	Girls, % (95%CI)	12–13 years, % (95%CI)	14–15 years, % (95%CI)
**Africa Region**					
Algeria	5.6 (4.8, 6.4)	7.1 (5.7, 8.5)	4.4 (3.5, 5.3)	4.9 (3.8, 6.0)	6.2 (5.1, 7.3)
Botswana	4.5 (3.0, 6.0)	7.1 (4.6, 9.5)	2.3 (0.8, 3.9)	3.3 (1.1, 5.4)	4.7 (3.0, 6.4)
Ghana	8.7 (7.3, 10.1)	7.9 (6.6, 9.3)	9.2 (7.4, 11.1)	8.7 (6.4, 10.9)	8.7 (7.2, 10.1)
Kenya	8.6 (6.7, 10.4)	9.9 (7.4, 12.4)	7.3 (5.5, 9.2)	10.5 (7.7, 13.3)	7.7 (5.4, 10.0)
Mozambique	7.4 (4.1, 10.7)	5.9 (2.6, 9.2)	8.4 (4.4, 12.5)	12.5 (10.9, 14.0)	5.6 (2.6, 8.5)
Namibia	5.5 (4.3, 6.7)	5.4 (3.6, 7.3)	5.5 (4.0, 7.0)	4.4 (2.7, 6.0)	5.9 (4.3, 7.5)
Senegal	10.2 (2.3, 18.2)	9.7 (3.2, 16.1)	11.4 (0.6, 22.1)	10.8 (0.0, 22.4)	9.9 (4.0, 15.9)
Swaziland	3.6 (1.9, 5.3)	3.5 (1.6, 5.4)	3.7 (1.8, 5.5)	3.0 (1.1, 4.8)	3.8 (1.9, 5.6)
Tanzania	7.3 (5.9, 8.6)	8.1 (6.1, 10.1)	6.3 (4.9, 7.7)	7.5 (5.4, 9.6)	7.1 (5.7, 8.5)
Uganda	6.3 (4.8, 7.9)	6.6 (4.2, 9.0)	6.2 (4.5, 7.9)	7.0 (4.5, 9.6)	6.2 (4.4, 7.9)
Zambia	12.5 (9.9, 15.1)	15.3 (11.4, 19.1)	9.7 (6.9, 12.5)	15.0 (12.7, 17.3)	11.2 (7.8, 14.6)
Zimbabwe	3.9 (3.0, 4.7)	4.0 (3.1, 5.0)	3.7 (2.5, 4.9)	4.7 (3.0, 6.3)	3.7 (2.7, 4.7)
**Pooled estimates**	**6.6 (5.4, 7.8)**	**7.2 (5.7, 8.6)**	**5.9 (4.7, 7.1)**	**7.5 (5.2, 9.7)**	**6.4 (5.2, 7.5)**
**I^2 (%)**	**87.4**	**84.7**	**83.2**	**93.3**	**80.5**
**European Region**					
Macedonia	2.1 (1.3, 2.9)	1.9 (0.4, 3.3)	2.4 (0.7, 4.1)	2.5 (1.7, 3.3)	1.8 (0.7, 3.0)
**Pooled estimates**	**–**	**–**	**–**	**–**	**–**
**I^2 (%)**	**–**	**–**	**–**	**–**	**–**
**America Region**					
Argentina	11.2 (10.0, 12.4)	11.4 (9.8, 13.0)	11.1 (9.9, 12.2)	8.2 (7.0, 9.3)	12.6 (11.1, 14.2)
Bahamas	15.6 (13.2, 18.0)	15.3 (12.9, 17.6)	16.0 (12.2, 19.8)	15.8 (13.6, 18.1)	15.4 (11.7, 19.0)
Barbados	14.3 (11.6, 16.9)	12.0 (8.7, 15.3)	16.5 (13.3, 19.7)	12.5 (9.5, 15.4)	14.9 (11.8, 18.0)
Belize	3.7 (3.0, 4.4)	3.3 (2.1, 4.6)	3.9 (3.0, 4.7)	3.0 (1.6, 4.4)	4.3 (3.4, 5.1)
Bolivia	10.8 (9.2, 12.3)	11.4 (9.7, 13.0)	10.3 (8.1, 12.5)	10.3 (8.1, 12.5)	11.0 (9.0, 12.9)
Cayman	11.2 (9.3, 13.1)	10.0 (7.3, 12.6)	12.5 (9.7, 15.4)	8.2 (5.8, 10.5)	13.4 (10.6, 16.1)
Costa Rica	9.6 (8.1, 11.1)	9.5 (8.0, 11.0)	9.6 (7.5, 11.7)	8.7 (5.7, 11.7)	10.1 (8.3, 11.9)
Curaçao	12.2 (10.1, 14.2)	10.4 (7.6, 13.2)	13.8 (10.8, 16.8)	12.2 (8.6, 15.8)	12.1 (9.9, 14.3)
Ecuador	12.3 (10.4, 14.2)	12.5 (10.9, 14.1)	11.3 (9.7, 13)	11.4 (9.3, 13.6)	13.3 (11, 15.7)
El Salvador	5.3 (3.9, 6.7)	4.8 (2.8, 6.7)	5.5 (4.3, 6.7)	3.6 (1.9, 5.2)	6.1 (4.5, 7.7)
Grenada	11.4 (9.3, 13.5)	10.4 (7.5, 13.3)	12.2 (9.5, 15.0)	10.5 (8.4, 12.5)	12.1 (9.1, 15.1)
Guatemala	4.5 (2.4, 6.6)	4.5 (2.5, 6.4)	4.2 (2.2, 6.1)	3.6 (1.4, 5.8)	5.0 (2.6, 7.3)
Guyana	9.2 (7.0, 11.3)	8.7 (5.7, 11.7)	9.6 (7.2, 12.0)	8.5 (4.4, 12.7)	9.4 (7.2, 11.6)
Honduras	7.5 (6.3, 8.7)	7.0 (4.8, 9.3)	7.7 (5.7, 9.7)	7.2 (5.8, 8.6)	7.7 (6.2, 9.2)
Peru	4.9 (3.7, 6.1)	4.9 (3.3, 6.6)	4.9 (3.3, 6.5)	4.7 (2.6, 6.8)	5.0 (3.8, 6.2)
Saint Kitts and Nevis	11.7 (10.0, 13.5)	11.5 (8.9, 14.2)	11.9 (9.6, 14.2)	9.7 (6.6, 12.7)	12.3 (10.3, 14.4)
Saint Lucia	16.5 (13.7, 19.2)	14.8 (11.2, 18.4)	17.8 (13.6, 22.0)	13.4 (10.1, 16.6)	18.6 (15.6, 21.6)

Saint Vincent and the Grenadines	8.7 (6.4, 11.1)	8.5 (5.7, 11.4)	8.4 (5.4, 11.5)	9.4 (5.3, 13.6)	8.0 (5.8, 10.2)
Trinidad and Tobago	11.3 (9.6, 13.0)	12.1 (9.2, 15.0)	10.3 (7.5, 13.1)	8.6 (6.0, 11.3)	13.5 (11.4, 15.6)
Uruguay	12.5 (10.6, 14.5)	12.7 (10.4, 14.9)	12.5 (10.3, 14.8)	9.6 (6.8, 12.4)	13.6 (11.6, 15.6)
Venezuela	6.4 (5.3, 7.5)	6.5 (4.8, 8.2)	6.3 (5.1, 7.5)	5.8 (4.8, 6.9)	7.3 (5.3, 9.3)
**Pooled estimates**	**10.0 (8.3, 11.6)**	**9.5 (7.9, 11.1)**	**10.1 (8.5, 11.7)**	**8.7 (7.2, 10.1)**	**10.6 (8.9, 12.4)**
**I^2 (%)**	**95.6**	**91.9**	**93.8**	**90.4**	**94.5**
**Eastern Mediterranean Region**					
Afghanistan	6.0 (4.0, 8.0)	6.4 (3.7, 9.0)	4.8 (2.4, 7.1)	8.6 (4.2, 13.0)	5.1 (3.2, 6.9)
Djibouti	6.0 (4.4, 7.6)	4.5 (2.6, 6.4)	8.1 (4.8, 11.4)	5.4 (2.3, 8.5)	6.1 (4.2, 7.9)
Egypt	12.9 (10.1, 15.6)	13.5 (9.2, 17.8)	12.2 (9.5, 14.9)	12.3 (9.6, 15.0)	13.5 (9.0, 18.0)
Iraq	7.3 (5.3, 9.4)	7.6 (5.0, 10.2)	7.1 (4.2, 9.9)	6.4 (3.8, 8.9)	7.9 (5.6, 10.2)
Jordan	6.8 (5.2, 8.3)	8.5 (7.0, 10.0)	5.1 (3.5, 6.8)	4.3 (0.8, 7.8)	7.1 (5.6, 8.6)
Kuwait	10.7 (8.8, 12.7)	10.1 (7.1, 13.2)	11.0 (8.7, 13.2)	11.5 (7.9, 15.1)	10.4 (8.1, 12.8)
Lebanon	5.8 (4.9, 6.6)	6.6 (4.9, 8.3)	5.0 (3.9, 6.1)	3.5 (1.8, 5.3)	7.4 (5.9, 8.8)
Libya	8.1 (6.0, 10.2)	10.6 (7.6, 13.6)	5.6 (3.8, 7.4)	7.0 (4.9, 9.0)	9.2 (6.2, 12.2)
Morocco	3.4 (2.2, 4.6)	3.7 (2.5, 4.8)	3.1 (1.6, 4.6)	3.0 (2.0, 4.1)	3.7 (2.2, 5.2)
Oman	7.9 (5.7, 10.0)	10.4 (8.3, 12.4)	5.5 (3.0, 8.0)	7.5 (2.6, 12.4)	8.0 (6.3, 9.6)
Pakistan	3.4 (2.6, 4.3)	4.5 (3.6, 5.4)	1.9 (1.6, 2.1)	2.9 (1.1, 4.8)	3.6 (2.8, 4.4)
Qatar	17.0 (13.4, 20.6)	19.5 (14.3, 24.6)	14.4 (9.2, 19.5)	14.1 (11.5, 16.6)	20.3 (13.2, 27.3)
Sudan	7.5 (5.8, 9.3)	8.7 (7.2, 10.3)	6.4 (3.4, 9.3)	5.9 (0.0, 12.0)	8.1 (6.1, 10.1)
Syrian Arab Republic	9.2 (7.2, 11.2)	10.5 (8.3, 12.7)	7.9 (6.2, 9.6)	9.3 (6.5, 12.1)	9.2 (7.0, 11.3)
Tunisia	6.0 (5.1, 6.9)	6.7 (5.2, 8.2)	5.2 (4.0, 6.4)	4.9 (3.8, 5.9)	6.9 (5.3, 8.5)
United Arab Emirates	8.4 (6.8, 10.1)	9.5 (6.6, 12.4)	7.5 (6.2, 8.9)	8.9 (6.1, 11.6)	8.2 (6.5, 10.0)
UNRWA	6.9 (5.6, 8.1)	8.5 (7.4, 9.7)	5.1 (4.0, 6.2)	6.7 (4.6, 8.8)	6.9 (5.9, 8.0)
Yemen	8.3 (4.7, 11.9)	9.0 (4.1, 13.9)	7.0 (1.2, 12.8)	7.7 (2.4, 13.1)	8.7 (4.2, 13.2)
**Pooled estimates**	**7.6 (6.4, 8.7)**	**8.3 (7.0, 9.7)**	**6.6 (5.1, 8.1)**	**7.1 (5.6, 8.6)**	**7.6 (6.5, 8.8)**
**I^2 (%)**	**89.9**	**88.5**	**95.1**	**87.4**	**86.3**
**Southeast Asia Region**					
Bangladesh	3.1 (1.4, 4.8)	3.0 (0.3, 5.8)	3.2 (1.8, 4.7)	5.5 (3.5, 7.4)	2.1 (1.0, 3.1)
India	5.9 (4.8, 7.0)	6.6 (5.4, 7.9)	5.0 (3.7, 6.2)	6.0 (4.3, 7.7)	5.9 (4.6, 7.1)
Indonesia	2.5 (2.0, 3.0)	3.2 (2.4, 4.0)	1.8 (1.2, 2.3)	2.6 (2.0, 3.2)	2.3 (1.7, 3.0)
Thailand	14.9 (12.2, 17.6)	14.4 (12.0, 16.7)	15.5 (11.7, 19.3)	14.3 (11.8, 16.8)	15.4 (11.7, 19.1)
Timor-Leste	20.9 (18.6, 23.2)	24.3 (21.2, 27.4)	17.2 (14.1, 20.3)	24.5 (20.6, 28.4)	19.7 (17.3, 22.1)
**Pooled estimates**	**9.3 (4.2, 14.5)**	**10.2 (4.6, 15.8)**	**8.1 (4.2, 12.0)**	**10.3 (4.9, 15.7)**	**8.8 (4.3, 13.3)**
**I^2 (%)**	**98.7**	**98.3**	**97.3**	**98.0**	**98.4**
**Western Pacific Region**					

Brunei Darussalam	3.5 (2.6, 4.4)	3.7 (2.5, 5.0)	3.3 (2.3, 4.4)	4.7 (2.3, 7.2)	3.0 (2.2, 3.7)
Cambodia	2.1 (1.3, 2.9)	2.1 (1.1, 3.2)	2.0 (1.3, 2.7)	2.2 (1.0, 3.4)	2.1 (1.1, 3.1)
China	4.9 (4.2, 5.6)	5.7 (4.7, 6.7)	4.0 (3.2, 4.8)	5.4 (4.4, 6.5)	4.5 (3.6, 5.3)
Cook	7.4 (3.7, 11.1)	8.4 (2.1, 14.7)	6.1 (2.0, 10.3)	9.8 (1.3, 18.2)	6.9 (3.7, 10.1)
Kiribati	20.6 (15.7, 25.5)	24.9 (18.2, 31.5)	16.9 (12.6, 21.3)	15.4 (10.3, 20.4)	22.7 (17.1, 28.4)
Laos	1.9 (0.8, 3.0)	2.0 (0.5, 3.4)	1.8 (0.8, 2.8)	0.4 (0.0, 1.4)	2.0 (0.8, 3.3)
Malaysia	5.0 (4.4, 5.6)	6.1 (5.4, 6.8)	3.9 (3.1, 4.7)	4.9 (3.9, 5.9)	5.0 (4.4, 5.6)
Mongolia	6.9 (5.8, 8.0)	7.4 (6.2, 8.6)	6.3 (4.6, 8.0)	6.7 (5.6, 7.8)	7.1 (5.3, 8.8)
Nauru	8.5 (5.5, 11.6)	13.0 (7.6, 18.4)	4.4 (1.3, 7.5)	4.9 (1.4, 8.4)	11.7 (7.0, 16.5)
Philippines	7.7 (4.2, 11.2)	7.7 (4.6, 10.8)	7.6 (3.3, 12.0)	6.6 (2.6, 10.5)	8.3 (3.6, 13.0)
Samoa	14.6 (12.7, 16.4)	17.9 (15.8, 20.0)	11.1 (8.8, 13.4)	16.6 (13.0, 20.2)	13.9 (11.8, 15.9)
Solomon	8.6 (7.0, 10.3)	9.8 (6.3, 13.4)	6.4 (4.0, 8.9)	5.2 (2.1, 8.3)	9.9 (7.3, 12.4)
Tokelau	17.0 (8.7, 25.3)	14.2 (3.7, 24.8)	21.8 (7.2, 36.3)	17.3 (5.3, 29.2)	16.6 (4.7, 28.6)
Tonga	11.4 (9.5, 13.3)	11.8 (8.7, 14.9)	10.8 (9.0, 12.6)	12.8 (9.2, 16.4)	10.9 (8.9, 12.8)
Tuvalu	38.7 (35.0, 42.4)	45.2 (39.7, 50.7)	32.6 (27.6, 37.7)	35.5 (30.6, 40.3)	42.9 (37.1, 48.7)
Vanuatu	5.7 (4.4, 6.9)	6.8 (4.2, 9.4)	4.6 (3.2, 6.1)	5.9 (2.9, 8.9)	5.4 (3.7, 7.1)
Vietnam	7.0 (5.1, 8.9)	6.6 (4.9, 8.3)	7.4 (4.6, 10.2)	–	6.9 (5.1, 8.7)
Wallis and Futuna	12.2 (9.6, 14.8)	12.4 (9.0, 15.8)	11.8 (8.7, 14.9)	12.1 (7.1, 17.1)	12.2 (9.1, 15.4)
**Pooled estimates**	**9.6 (7.6, 11.6)**	**10.5 (8.3, 12.8)**	**7.8 (6.1, 9.5)**	**9.0 (6.7, 11.3)**	**9.4 (7.5, 11.3)**
**I^2 (%)**	**97.5**	**96.6**	**95.1**	**96.2**	**96.2**
**Total**					
**Pooled estimates**	**7.4 (4.4, 10.3)**	**7.7 (5.0, 10.5)**	**6.7 (4.6, 8.8)**	**7.3 (4.4, 10.2)**	**7.3 (4.6, 10.1)**
**I^2 (%)**	**96.2**	**93.0**	**89.0**	**94.4**	**94.9**

Table S3 Prevalence of young adolescents who rarely or never wash hands after using the toilet or latrine

	Total, % (95%CI)	Boys, % (95%CI)	Girls, % (95%CI)	12–13 years, % (95%CI)	14–15 years, % (95%CI)
**Africa Region**					
Algeria	3.1 (2.4, 3.8)	3.4 (2.4, 4.4)	2.6 (1.8, 3.5)	2.9 (1.4, 4.3)	3.3 (2.3, 4.4)
Botswana	5.1 (4.1, 6.2)	6.9 (5.4, 8.4)	3.7 (2.1, 5.3)	3.8 (1.6, 6.1)	5.4 (4.1, 6.6)
Ghana	11.8 (10.1, 13.5)	12.4 (10.2, 14.5)	11.2 (9.0, 13.4)	12.5 (9.7, 15.3)	11.5 (10.0, 12.9)
Kenya	13.9 (11.7, 16.1)	15.5 (12.7, 18.3)	15.5 (12.7, 18.3)	15.3 (12.5, 18.1)	13.3 (10.7, 15.8)
Mozambique	7.8 (4.0, 11.6)	6.7 (5.2, 8.1)	8.4 (3.4, 13.5)	11.3 (2.6, 19.9)	6.6 (3.2, 10.0)
Namibia	6.4 (4.9, 7.9)	8.4 (6.4, 10.4)	4.9 (3.2, 6.5)	7.6 (5.5, 9.6)	5.9 (4.0, 7.9)
Senegal	10.0 (2.2, 17.8)	9.4 (2.1, 16.7)	11.0 (1.1, 21.0)	11.5 (0.0, 23.2)	9.1 (3.4, 14.8)
Swaziland	2.8 (2.2, 3.5)	3.4 (2.1, 4.7)	2.5 (1.8, 3.3)	2.1 (1.1, 3.0)	3.1 (2.3, 3.9)
Tanzania	17.3 (13.9, 20.7)	17.6 (13.3, 22.0)	16.7 (13.1, 20.4)	17.4 (13.1, 21.6)	17.3 (14.0, 20.6)
Uganda	7.9 (5.6, 10.3)	8.4 (5.2, 11.6)	7.2 (4.4, 10.0)	6.2 (3.8, 8.6)	8.3 (5.8, 10.9)
Zambia	15.4 (12.6, 18.2)	16.3 (11.8, 20.7)	13.6 (10.4, 16.7)	17.4 (14.7, 20.0)	14.4 (11.1, 17.7)
Zimbabwe	6.2 (4.9, 7.5)	7.6 (5.8, 9.5)	4.8 (3.5, 6.1)	5.8 (4.2, 7.3)	6.3 (4.9, 7.7)
**Pooled estimates**	**8.7 (6.5, 10.9)**	**9.3 (7.0, 11.6)**	**8.1 (5.8, 10.3)**	**9.1 (6.1, 12.2)**	**8.5 (6.2, 10.8)**
**I^2 (%)**	**96.5**	**93.8**	**95.1**	**95.6**	**95.4**
**European Region**					
Macedonia	1.8 (0.9, 2.8)	1.8 (0.9, 2.8)	2.2 (0.8, 3.6)	2.2 (0.8, 3.6)	2.1 (1.1, 3.0)
**Pooled estimates**	**–**	**–**	**–**	**–**	**–**
**I^2 (%)**	**–**	**–**	**–**	**–**	**–**
**America Region**					
Argentina	5.9 (4.9, 6.9)	7.1 (5.5, 8.6)	4.7 (3.8, 5.7)	4.5 (3.4, 5.7)	6.5 (5.4, 7.6)
Bahamas	4.5 (3.2, 5.7)	4.2 (2.3, 6.1)	4.8 (3.5, 6.1)	3.5 (2.1, 5.0)	5.5 (3.2, 7.8)
Barbados	1.7 (1.1, 2.4)	2.0 (1.0, 2.9)	1.5 (0.6, 2.3)	0.7 (0.0, 1.4)	2.1 (1.3, 2.9)
Belize	1.6 (1.1, 2.2)	2.6 (1.5, 3.7)	0.7 (0.0, 1.5)	1.1 (0.3, 1.8)	2.1 (1.2, 3.0)
Bolivia	7.3 (5.9, 8.6)	7.5 (5.4, 9.5)	7.0 (5.5, 8.5)	9.8 (7.7, 12.0)	6.3 (4.9, 7.7)
Cayman	4.7 (3.4, 6.0)	5.3 (3.3, 7.3)	4.0 (2.4, 5.7)	4.3 (2.5, 6.2)	4.9 (3.2, 6.7)
Costa Rica	2.2 (1.5, 2.8)	2.5 (1.4, 3.5)	1.9 (1.1, 2.6)	1.9 (0.7, 3.0)	2.3 (1.5, 3.1)
Curaçao	3.6 (2.6, 4.5)	4.1 (2.6, 5.6)	3.0 (1.9, 4.2)	2.7 (1.4, 3.9)	4.0 (2.8, 5.1)
Ecuador	6.5 (5.1, 8.0)	7.0 (5.9, 8.0)	5.3 (3.9, 6.7)	6.4 (4.9, 8.0)	6.6 (4.0, 9.3)
El Salvador	4.0 (2.3, 5.8)	4.2 (1.6, 6.9)	3.3 (1.8, 4.8)	3.1 (1.1, 5.1)	4.4 (2.6, 6.3)
Grenada	3.5 (2.7, 4.3)	4.3 (2.9, 5.8)	2.9 (1.8, 4.1)	3.6 (1.9, 5.2)	3.5 (2.4, 4.5)
Guatemala	2.7 (1.1, 4.2)	3.1 (1.3, 4.8)	2.0 (1.1, 2.9)	3.3 (1.9, 4.7)	2.4 (0.5, 4.3)
Guyana	6.4 (4.4, 8.3)	6.9 (4.5, 9.3)	5.6 (3.3, 7.8)	6.6 (3.1, 10.1)	6.2 (4.4, 8.1)
Honduras	5.2 (3.7, 6.7)	4.6 (2.5, 6.6)	5.6 (3.5, 7.6)	6.1 (4.0, 8.2)	6.1 (4.0, 8.2)
Peru	6.6 (5.4, 7.8)	6.4 (4.6, 8.2)	6.8 (5.2, 8.3)	6.8 (4.5, 9.2)	6.5 (5.2, 7.8)
Saint Kitts and Nevis	2.9 (2.0, 3.9)	4.3 (2.6, 6.0)	1.6 (0.7, 2.4)	1.9 (0.5, 3.3)	3.2 (2.1, 4.4)
Saint Lucia	4.4 (3.2, 5.5)	5.1 (3.0, 7.3)	3.7 (2.4, 5.1)	4.3 (2.8, 5.8)	4.4 (2.7, 6.1)

Saint Vincent and the Grenadines	4.1 (2.8, 5.4)	3.9 (2.1, 5.8)	3.7 (1.8, 5.5)	3.6 (2.1, 5.1)	4.6 (2.9, 6.4)
Trinidad and Tobago	2.7 (1.9, 3.5)	2.8 (2.0, 3.6)	2.4 (1.4, 3.5)	1.6 (0.6, 2.6)	3.6 (2.1, 5.1)
Uruguay	7.6 (5.8, 9.3)	7.6 (5.8, 9.3)	6.3 (4.6, 8.0)	5.6 (3.9, 7.3)	7.3 (5.7, 9.0)
Venezuela	3.1 (2.6, 3.7)	3.9 (2.8, 4.9)	2.4 (1.5, 3.3)	3.2 (2.5, 3.8)	3.1 (2.1, 4.0)
**Pooled estimates**	**4.2 (3.5, 5.0)**	**4.7 (3.8, 5.5)**	**3.7 (2.9, 4.4)**	**3.8 (3.0, 4.7)**	**4.4 (3.7, 5.2)**
**I^2 (%)**	**91.5**	**84.6**	**89.1**	**88.9**	**85.7**
**Eastern Mediterranean Region**					
Afghanistan	5.6 (3.0, 8.2)	5.8 (2.3, 9.2)	4.6 (1.2, 8.0)	8.6 (2.6, 14.6)	4.5 (2.1, 7.0)
Djibouti	12.9 (11.3, 14.6)	13.5 (11.4, 15.7)	11.8 (8.9, 14.7)	15.3 (11.1, 19.5)	12.5 (10.7, 14.2)
Egypt	9.3 (6.4, 12.3)	11.3 (6.4, 16.2)	7.5 (4.3, 10.6)	9.4 (6.6, 12.3)	9.2 (4.7, 13.8)
Iraq	8.1 (6.0, 10.2)	9.9 (7.3, 12.5)	6.0 (4.0, 8.1)	8.2 (4.8, 11.6)	8.1 (5.5, 10.7)
Jordan	7.0 (4.4, 9.6)	8.8 (7.0, 10.6)	5.2 (2.9, 7.5)	7.8 (2.3, 13.2)	6.9 (4.4, 9.4)
Kuwait	6.2 (4.5, 7.9)	7.6 (5.2, 9.9)	4.8 (3.2, 6.4)	5.4 (2.8, 8.1)	6.5 (4.6, 8.5)
Lebanon	2.4 (1.1, 3.6)	3.1 (1.0, 5.2)	1.7 (0.7, 2.7)	1.9 (0.6, 3.1)	2.7 (0.9, 4.5)
Libya	6.8 (5.2, 8.4)	8.6 (6.1, 11.0)	5.1 (3.5, 6.6)	6.8 (5.3, 8.2)	6.8 (4.6, 9.1)
Morocco	5.3 (4.5, 6.1)	5.6 (3.7, 7.5)	5.6 (3.7, 7.5)	5.6 (3.7, 7.5)	5.6 (3.7, 7.5)
Oman	8.8 (6.9, 10.7)	8.8 (6.9, 10.7)	8.8 (6.9, 10.7)	8.8 (6.9, 10.7)	8.8 (6.9, 10.7)
Pakistan	3.3 (2.3, 4.3)	3.3 (2.3, 4.3)	3.3 (2.3, 4.3)	3.3 (2.3, 4.3)	3.3 (2.3, 4.3)
Qatar	4.0 (3.1, 4.9)	4.5 (3.3, 5.7)	3.6 (2.5, 4.7)	3.5 (2.3, 4.7)	4.4 (2.9, 5.9)
Sudan	11.9 (9.8, 14.1)	11.9 (9.8, 14.1)	11.9 (9.8, 14.1)	11.9 (9.8, 14.1)	11.9 (9.8, 14.1)
Syrian Arab Republic	3.8 (2.1, 5.5)	4.2 (2.9, 5.4)	3.4 (1.3, 5.5)	4.5 (2.3, 6.7)	3.2 (1.7, 4.7)
Tunisia	4.0 (3.1, 4.9)	4.5 (3.3, 5.7)	3.6 (2.5, 4.7)	3.5 (2.3, 4.7)	4.4 (2.9, 5.9)
United Arab Emirates	3.4 (2.4, 4.4)	4.3 (3.2, 5.5)	2.8 (2.0, 3.6)	3.3 (1.9, 4.7)	3.5 (2.4, 4.5)
UNRWA	4.8 (3.9, 5.7)	6.7 (5.6, 7.7)	3.0 (2.4, 3.6)	5.5 (4.2, 6.8)	4.4 (3.6, 5.2)
Yemen	14.7 (11.4, 17.9)	15.0 (10.8, 19.2)	12.6 (6.6, 18.6)	14.1 (7.1, 21.0)	15.1 (11.0, 19.1)
**Pooled estimates**	**6.6 (5.3, 7.8)**	**7.3 (6.0, 8.7)**	**5.4 (4.3, 6.4)**	**6.4 (5.1, 7.8)**	**6.5 (5.2, 7.9)**
**I^2 (%)**	**93.3**	**91.3**	**89.9**	**89.3**	**91.4**
**Southeast Asia Region**					
Bangladesh	1.9 (0.2, 3.6)	2.2 (0.0, 5.0)	1.5 (0.4, 2.7)	3.7 (2.3, 5.1)	1.2 (0.7, 1.7)
India	3.3 (2.6, 4.0)	3.3 (2.4, 4.1)	3.4 (2.5, 4.2)	3.5 (2.4, 4.5)	3.2 (2.5, 4.0)
Indonesia	2.3 (1.8, 2.8)	3.2 (2.3, 4.2)	1.4 (1.0, 1.8)	2.3 (1.7, 3.0)	2.2 (1.7, 2.8)
Thailand	6.5 (5.2, 7.8)	8.2 (5.9, 10.5)	4.8 (2.9, 6.8)	7.4 (5.6, 9.3)	5.8 (4.4, 7.2)
Timor-Leste	27.5 (24.1, 30.9)	29.8 (25.7, 34.0)	24.9 (20.1, 29.8)	30.3 (24.7, 35.9)	26.6 (23.5, 29.7)
**Pooled estimates**	**7.7 (4.3, 11.1)**	**8.8 (4.4, 13.2)**	**5.5 (3.0, 8.0)**	**7.7 (4.5, 10.9)**	**7.1 (4.1, 10.1)**
**I^2 (%)**	**98.3**	**97.6**	**96.5**	**96.6**	**98.6**
**Western Pacific Region**					

Brunei Darussalam	2.9 (1.7, 4.1)	3.6 (1.6, 5.5)	2.2 (1.2, 3.2)	3.6 (0.8, 6.4)	2.6 (1.6, 3.6)
Cambodia	3.0 (2.0, 4.1)	3.6 (2.2, 5.0)	2.5 (1.3, 3.7)	2.3 (0.3, 4.3)	3.3 (2.1, 4.5)
China	3.0 (2.4, 3.5)	3.7 (3.0, 4.4)	2.2 (1.7, 2.8)	3.0 (2.4, 3.6)	3.0 (2.3, 3.7)
Cook	4.3 (1.6, 7.0)	5.8 (0.6, 11.1)	2.5 (0.7, 4.2)	6.1 (0.4, 11.8)	3.9 (1.5, 6.4)
Kiribati	16.1 (12.7, 19.4)	19.6 (15.0, 24.1)	13.0 (10.1, 16.0)	14.1 (9.8, 18.3)	16.9 (13.1, 20.6)
Laos	3.8 (2.1, 5.6)	4.3 (2.2, 6.4)	3.4 (1.4, 5.4)	3.6 (0.0, 8.1)	3.9 (2.1, 5.6)
Malaysia	6.0 (5.3, 6.7)	7.1 (6.1, 8.0)	4.9 (4.1, 5.6)	6.5 (5.4, 7.6)	5.7 (4.9, 6.5)
Mongolia	10.5 (8.8, 12.2)	11.8 (9.6, 14.0)	9.1 (7.2, 11.0)	10.1 (8.1, 12.1)	10.8 (8.5, 13.1)
Nauru	8.1 (5.1, 11.1)	12.6 (7.2, 18.0)	3.3 (0.9, 5.8)	6.9 (2.7, 11.1)	9.2 (5.0, 13.4)
Philippines	6.3 (3.3, 9.3)	7.0 (4.3, 9.6)	5.6 (2.1, 9.2)	4.3 (1.4, 7.2)	7.4 (3.4, 11.5)
Samoa	17.1 (14.6, 19.6)	18.8 (15.8, 21.8)	14.8 (11.5, 18.2)	18.1 (13.6, 22.6)	16.7 (14.0, 19.4)
Solomon	8.9 (6.5, 11.4)	9.5 (6.5, 12.5)	8.5 (5.8, 11.2)	5.7 (2.9, 8.5)	10.1 (6.9, 13.4)
Tokelau	5.4 (0.8, 10.0)	5.5 (0.0, 11.9)	5.7 (0.0, 12.8)	4.3 (0.0, 9.9)	6.7 (0.0, 14.6)
Tonga	6.7 (5.3, 8.1)	6.7 (4.6, 8.7)	6.7 (5.0, 8.4)	6.4 (3.7, 9.0)	6.8 (5.2, 8.4)
Tuvalu	17.9 (14.9, 20.9)	25.2 (20.3, 30.0)	10.7 (7.3, 14.1)	17.2 (13.3, 21.0)	18.9 (14.2, 23.6)
Vanuatu	6.8 (4.1, 9.5)	8.5 (3.8, 13.3)	5.2 (2.8, 7.5)	8.3 (4.8, 11.7)	5.2 (2.6, 7.8)
Vietnam	2.2 (1.5, 2.9)	2.8 (1.6, 4.0)	1.6 (0.8, 2.5)	6.4 (0.0, 19.7)	2.1 (1.4, 2.8)
Wallis and Futuna	7.9 (5.8, 10.0)	7.9 (4.6, 11.3)	7.8 (5.0, 10.6)	10.1 (6.0, 14.1)	6.5 (4.3, 8.6)
**Pooled estimates**	**7.4 (5.8, 9.0)**	**8.7 (6.8, 10.7)**	**5.8 (4.4, 7.1)**	**7.4 (5.6, 9.3)**	**7.3 (5.7, 8.9)**
**I^2 (%)**	**95.9**	**94.2**	**92.9**	**90.7**	**94.4**
**Total**					
**Pooled estimates**	**5.9 (3.8, 7.9)**	**6.5 (4.1, 9.0)**	**5.0 (3.6, 6.3)**	**5.8 (3.9, 7.7)**	**5.8 (3.9, 7.7)**
**I^2 (%)**	**93.1**	**94.1**	**84.1**	**88.2**	**91.5**

Table S4 Prevalence of young adolescents who rarely or never wash hands using soap

	Total, % (95%CI)	Boys, % (95%CI)	Girls, % (95%CI)	12–13 years, % (95%CI)	14–15 years, % (95%CI)
**Africa Region**					
Algeria	5.1 (4.3, 5.8)	6.1 (4.9, 7.3)	4.1 (3, 5.2)	4.8 (3.6, 6.1)	5.3 (4.2, 6.4)
Botswana	17.0 (13.3, 20.8)	17.9 (13.7, 22.2)	16.1 (12, 20.2)	17.6 (11.3, 23.9)	16.9 (13.1, 20.7)
Ghana	13.5 (11.7, 15.4)	13.8 (11.9, 15.8)	13.1 (10.7, 15.4)	12.7 (10.4, 15.1)	13.9 (12, 15.9)
Kenya	16.6 (13.8, 19.5)	17.5 (14.8, 20.1)	15.8 (11.6, 20)	16.8 (14, 19.5)	16.6 (13.2, 19.9)
Mozambique	12.0 (5.7, 18.3)	7.7 (5.1, 10.4)	15.9 (5.6, 26.3)	16.6 (3.3, 30.0)	10.3 (4.1, 16.4)
Namibia	10.8 (8.5, 13.0)	12.7 (9.6, 15.7)	9.0 (6.9, 11.1)	9.6 (6.3, 12.8)	11.3 (8.6, 13.9)
Senegal	14.7 (7.6, 21.8)	13.7 (7.4, 20.0)	16.2 (7.0, 25.4)	16.1 (5.1, 27.0)	13.9 (8.9, 18.9)
Swaziland	11.6 (9.7, 13.5)	13.5 (10.9, 16.2)	10.4 (8.5, 12.3)	11.2 (7.4, 15.0)	11.8 (10.0, 13.6)
Tanzania	20.5 (17.7, 23.3)	22.1 (18.4, 25.9)	18.8 (15.3, 22.2)	20.2 (16.4, 23.9)	20.8 (18.0, 23.5)
Uganda	14.7 (11.8, 17.5)	14.7 (10.6, 18.7)	14.6 (11.3, 17.9)	14.4 (11.2, 17.5)	14.7 (11.7, 17.7)
Zambia	20.6 (17.7, 23.4)	19.0 (15.7, 22.3)	21.7 (18.2, 25.2)	22.6 (16.9, 28.3)	19.5 (16.4, 22.6)
Zimbabwe	6.2 (4.9, 7.5)	7.6 (5.8, 9.5)	4.8 (3.5, 6.1)	5.8 (4.2, 7.3)	6.3 (4.9, 7.7)
**Pooled estimates**	**13.5 (10.2, 16.8)**	**13.7 (10.6, 16.8)**	**13.0 (9.7, 16.3)**	**13.5 (10.0, 17.0)**	**13.4 (10.2, 16.6)**
**I^2 (%)**	**96.8**	**94.2**	**95.5**	**93.9**	**95.6**
**European Region**					
Macedonia	3.8 (2.4, 5.2)	4.2 (2.0, 6.4)	3.3 (1.6, 5.1)	3.5 (1.3, 5.7)	3.9 (2.4, 5.4)
**Pooled estimates**	**–**	**–**	**–**	**–**	**–**
**I^2 (%)**	**–**	**–**	**–**	**–**	**–**
**America Region**					
Argentina	6.2 (5.4, 7.1)	8.5 (6.9, 10.1)	4.1 (3.6, 4.7)	4.3 (3.3, 5.3)	7.2 (6.2, 8.1)
Bahamas	8.5 (6.9, 10.2)	8.9 (6.0, 11.8)	8.2 (6.5, 10.0)	8.6 (6.6, 10.6)	8.5 (5.9, 11.0)
Barbados	7.8 (6.1, 9.5)	4.2 (2.0, 6.4)	3.3 (1.6, 5.1)	3.5 (1.3, 5.7)	3.9 (2.4, 5.4)
Belize	4.5 (3.3, 5.7)	4.5 (2.4, 6.6)	4.6 (2.5, 6.6)	4.3 (2.6, 5.9)	4.8 (3.5, 6.0)
Bolivia	16.0 (13.6, 18.4)	18.3 (15.3, 21.3)	13.7 (11.2, 16.2)	14.8 (11.3, 18.3)	16.5 (14.1, 18.9)
Cayman	4.7 (3.4, 6.0)	5.3 (3.3, 7.3)	4.0 (2.4, 5.7)	4.3 (2.5, 6.2)	4.9 (3.2, 6.7)
Costa Rica	5.4 (4.3, 6.5)	5.7 (3.5, 7.9)	5.1 (4.0, 6.3)	4.5 (2.5, 6.5)	5.9 (4.7, 7.0)
Curaçao	6.4 (4.8, 8.0)	6.7 (4.6, 8.9)	6.2 (4.1, 8.2)	7.0 (4.5, 9.5)	6.1 (4.6, 7.6)
Ecuador	9.5 (8, 11)	9.7 (8.9, 10.6)	8.5 (7.3, 9.7)	9.1 (8, 10.1)	10.1 (7.4, 12.7)
El Salvador	5.6 (4.1, 7.1)	5.4 (3.2, 7.6)	5.2 (3.3, 7.1)	4.8 (2.3, 7.3)	5.9 (4.2, 7.6)
Grenada	10.2 (8.2, 12.2)	9.3 (6.1, 12.5)	10.9 (8.4, 13.4)	9.8 (6.8, 12.9)	10.5 (8.2, 12.8)
Guatemala	5.6 (3.9, 7.3)	6.8 (4.8, 8.8)	4.5 (2.1, 6.8)	6.8 (4.6, 8.9)	5.0 (2.9, 7.1)
Guyana	10.7 (8.1, 13.2)	11.7 (7.8, 15.7)	9.6 (6.9, 12.3)	9.0 (5.8, 12.1)	11.3 (8.5, 14.1)
Honduras	58.7 (54.6, 62.7)	56.0 (51.6, 60.4)	60.9 (56.0, 65.7)	53.7 (47.4, 59.9)	63.2 (58.1, 68.3)
Peru	7.5 (5.8, 9.2)	8.7 (6.5, 10.8)	6.4 (4.4, 8.5)	8.8 (5.5, 12.1)	7.1 (5.5, 8.6)
Saint Kitts and Nevis	2.9 (2.0, 3.9)	4.3 (2.6, 6.0)	1.6 (0.7, 2.4)	1.9 (0.5, 3.3)	3.2 (2.1, 4.4)
Saint Lucia	11.1 (8.9, 13.3)	13.0 (9.0, 16.9)	9.6 (7.0, 12.2)	8.6 (5.7, 11.6)	12.8 (10.2, 15.4)

Saint Vincent and the Grenadines	8.4 (6.4, 10.3)	8.0 (4.8, 11.3)	8.7 (6.3, 11.2)	9.7 (6.4, 12.9)	7.0 (5.0, 8.9)
Trinidad and Tobago	10.0 (8.9, 11.0)	11.1 (8.8, 13.3)	8.7 (6.3, 11.1)	7.6 (5.1, 10.1)	11.9 (9.9, 13.9)
Uruguay	3.6 (2.5, 4.7)	4.5 (3.0, 6.0)	2.8 (1.6, 4.0)	7.6 (5.1, 10.1)	11.9 (9.9, 13.9)
Venezuela	3.1 (2.6, 3.7)	3.9 (2.8, 4.9)	2.4 (1.5, 3.3)	3.2 (2.5, 3.8)	3.1 (2.1, 4.0)
**Pooled estimates**	**9.5 (7.4, 11.5)**	**9.9 (7.5, 12.4)**	**8.5 (6.5, 10.5)**	**8.3 (6.5, 10.1)**	**10.0 (7.8, 12.2)**
**I^2 (%)**	**98.1**	**97.0**	**97.5**	**95.4**	**97.4**
**Eastern Mediterranean Region**					
Afghanistan	11.3 (6.7, 15.9)	15.0 (9.1, 21.0)	7.2 (5.9, 8.5)	9.7 (5.6, 13.8)	11.9 (6.0, 17.8)
Djibouti	11.8 (9.1, 14.5)	11.9 (7.8, 16.0)	11.8 (9.1, 14.5)	9.2 (6.1, 12.4)	12.3 (9.4, 15.2)
Egypt	7.7 (5.0, 10.3)	10.3 (5.9, 14.7)	5.1 (3.3, 6.9)	7.7 (5.5, 10.0)	7.6 (3.5, 11.6)
Iraq	3.9 (2.7, 5.2)	4.0 (2.5, 5.5)	3.9 (2.8, 4.9)	3.6 (1.7, 5.5)	4.1 (2.8, 5.4)
Jordan	8.8 (6.6, 10.9)	10.7 (8.8, 12.7)	6.9 (4.3, 9.5)	12.3 (5.1, 19.4)	8.3 (6.5, 10.0)
Kuwait	6.9 (4.7, 9.1)	7.9 (4.8, 11.0)	5.6 (4.4, 6.7)	4.9 (2.5, 7.3)	7.7 (5.0, 10.4)
Lebanon	1.8 (1.1, 2.5)	2.8 (1.2, 4.3)	0.9 (0.2, 1.6)	2.2 (0.9, 3.4)	1.4 (0.7, 2.1)
Libya	7.2 (5.7, 8.8)	9.4 (7.0, 11.8)	5.2 (3.4, 7.0)	7.3 (5.1, 9.5)	7.2 (5.4, 9.1)
Morocco	7.4 (5.9, 8.9)	8.8 (6.6, 11.0)	5.8 (4.5, 7.1)	7.3 (5.4, 9.3)	7.5 (5.8, 9.1)
Oman	7.3 (5.3, 9.4)	9.2 (6.3, 12.1)	5.2 (3.7, 6.8)	6.8 (3.7, 9.9)	7.5 (5.6, 9.4)
Pakistan	8.1 (6.0, 10.1)	10.9 (8.3, 13.5)	3.6 (3.0, 4.2)	8.4 (5.9, 10.9)	8.0 (5.7, 10.3)
Qatar	15.8 (12.1, 19.5)	19.1 (14.3, 24.0)	12.9 (6.8, 18.9)	14.4 (11.3, 17.5)	17.4 (10.3, 24.5)
Sudan	19.9 (15.2, 24.7)	21.9 (17.7, 26.1)	17.8 (11.0, 24.6)	18.6 (8.7, 28.6)	20.3 (15.9, 24.7)
Syrian Arab Republic	6.6 (4.7, 8.5)	8.5 (6.9, 10.2)	4.6 (3.1, 6.1)	6.7 (4.6, 8.7)	6.5 (4.2, 8.9)
Tunisia	4.7 (3.5, 5.9)	5.7 (4, 7.4)	3.7 (2.5, 4.9)	4.4 (2.7, 6)	5.0 (3.7, 6.2)
United Arab Emirates	4.2 (3.0, 5.4)	5.4 (4.0, 6.8)	3.3 (2.1, 4.5)	4.0 (2.8, 5.2)	4.2 (2.8, 5.6)
UNRWA	4.8 (3.9, 5.7)	6.7 (5.6, 7.7)	3.0 (2.4, 3.6)	5.5 (4.2, 6.8)	4.4 (3.6, 5.2)
Yemen	21.6 (16.2, 26.9)	24.7 (19.3, 30.1)	15.5 (5.8, 25.2)	17.6 (8.3, 26.9)	24.1 (17.3, 30.9)
**Pooled estimates**	**8.2 (6.6, 9.7)**	**10.0 (8.2, 11.9)**	**5.4 (4.3, 6.4)**	**7.1 (5.7, 8.5)**	**8.1 (6.5, 9.8)**
**I^2 (%)**	**93.9**	**92.0**	**91.4**	**84.9**	**93.7**
**Southeast Asia Region**					
Bangladesh	5.0 (2.1, 7.9)	6.2 (1.4, 11.0)	3.0 (0.9, 5.0)	4.7 (3.0, 6.4)	5.1 (1.4, 8.9)
India	12.9 (11.2, 14.6)	13.5 (11.7, 15.4)	12.0 (9.8, 14.2)	12.2 (9.9, 14.6)	13.3 (11.2, 15.4)
Indonesia	3.9 (3.2, 4.7)	5.7 (4.6, 6.7)	2.2 (1.6, 2.8)	3.9 (3.2, 4.6)	3.9 (3.0, 4.8)
Thailand	14.2 (11.8, 16.7)	16.2 (12.8, 19.6)	12.4 (10.0, 14.7)	13.1 (10.5, 15.8)	15.1 (12.5, 17.7)
Timor-Leste	19.1 (16.6, 21.6)	22.3 (18.0, 26.6)	15.6 (12.7, 18.4)	21.2 (16.2, 26.2)	18.4 (15.8, 21.0)
**Pooled estimates**	**11.0 (4.9, 17.1)**	**12.7 (6.9, 18.5)**	**9.0 (3.4, 14.5)**	**10.6 (6.0, 15.2)**	**11.2 (4.7, 17.6)**
**I^2 (%)**	**98.3**	**96.5**	**98.0**	**96.7**	**97.9**
**Western Pacific Region**					

Brunei Darussalam	9.7 (8.0, 11.4)	11.7 (8.6, 14.7)	7.9 (6.1, 9.6)	10.1 (7.2, 13.0)	9.6 (7.6, 11.5)
Cambodia	2.9 (2.1, 3.6)	3.4 (1.9, 4.9)	2.3 (0.8, 3.8)	2.6 (0.8, 4.5)	3.0 (2.2, 3.7)
China	3.0 (2.4, 3.5)	3.7 (3.0, 4.4)	2.2 (1.7, 2.8)	3.0 (2.4, 3.6)	3.0 (2.3, 3.7)
Cook	7.9 (4.7, 11.0)	10.3 (6.0, 14.6)	5.7 (1.6, 9.8)	7.1 (0.0, 14.4)	8.1 (4.5, 11.7)
Kiribati	15.3 (12.8, 17.8)	18.0 (14.5, 21.5)	13.0 (9.8, 16.3)	14.0 (10.2, 17.9)	15.9 (13.4, 18.3)
Laos	8.5 (6.3, 10.7)	8.2 (5.6, 10.9)	8.6 (5.8, 11.5)	9.6 (3.1, 16.0)	8.4 (6.3, 10.4)
Malaysia	13.6 (12.6, 14.6)	16.1 (14.8, 17.4)	11.2 (10.1, 12.3)	13.7 (12.4, 15.1)	13.7 (12.4, 15.1)
Mongolia	3.0 (2.3, 3.8)	3.6 (2.6, 4.5)	2.5 (1.7, 3.3)	3.0 (1.8, 4.1)	3.1 (2.1, 4.0)
Nauru	8.1 (5.1, 11.1)	12.6 (7.2, 18.0)	3.3 (0.9, 5.8)	6.9 (2.7, 11.1)	9.2 (5.0, 13.4)
Philippines	7.9 (4.8, 11.0)	8.4 (5.6, 11.1)	7.5 (3.4, 11.5)	6.8 (2.7, 10.9)	8.5 (4.6, 12.4)
Samoa	19.3 (16.8, 21.8)	22.1 (17.6, 26.5)	16.2 (12.8, 19.6)	22.0 (17.5, 26.6)	18.4 (16.1, 20.7)
Solomon	10.1 (6.0, 14.3)	11.4 (6.3, 16.5)	8.8 (4.1, 13.5)	8.8 (4.6, 12.9)	10.6 (6.2, 15.1)
Tokelau	5.4 (0.8, 10.0)	5.5 (0.0, 11.9)	5.7 (0.0, 12.8)	4.3 (0.0, 9.9)	6.7 (0.0, 14.6)
Tonga	17.6 (15.6, 19.6)	16.3 (13.7, 18.8)	18.9 (15.9, 21.8)	21.1 (17.3, 24.9)	16.2 (13.7, 18.7)
Tuvalu	17.9 (14.9, 20.9)	25.2 (20.3, 30.0)	10.7 (7.3, 14.1)	17.2 (13.3, 21.0)	18.9 (14.2, 23.6)
Vanuatu	14.6 (10.2, 18.9)	17.5 (13.1, 21.9)	12.1 (7.1, 17.1)	13.6 (7.1, 20.2)	15.6 (10.4, 20.8)
Vietnam	2.2 (1.5, 2.9)	2.8 (1.6, 4.0)	1.6 (0.8, 2.5)	6.4 (0.0, 19.7)	2.1 (1.4, 2.8)
Wallis and Futuna	8.2 (6.2, 10.2)	9.1 (5.5, 12.7)	7.2 (4.0, 10.3)	8.7 (6.1, 11.3)	7.8 (5.1, 10.5)
**Pooled estimates**	**9.7 (7.3, 12.0)**	**11.2 (8.5, 14.0)**	**7.9 (5.8, 10.0)**	**9.9 (7.1, 12.7)**	**9.8 (7.4, 12.1)**
**I^2 (%)**	**98.2**	**97.2**	**96.6**	**96.1**	**97.5**
**Total**					
**Pooled estimates**	**9.0 (6.2, 11.8)**	**10.0 (7.2, 12.8)**	**7.5 (5.2, 9.8)**	**8.5 (6.1, 10.8)**	**9.1 (6.3, 12.0)**
**I^2 (%)**	**89.7**	**84.5**	**86.7**	**83.1**	**89.0**

Table S5 Prevalence of daily tooth brushing among young adolescents

	Total, % (95%CI)			Boys, % (95%CI)			Girls, % (95%CI)			12–13 years, % (95%CI)			14–15 years, % (95%CI)		
	0	1–3 times	>3 times	0	1–3 times	>3 times	0	1–3 times	>3 times	0	1–3 times	>3 times	0	1–3 times	>3 times
**Africa Region**															
Algeria	19.7 (17.1, 22.3)	67.1 (64.5, 69.8)	13.2 (11.2, 15.1)	23.4 (20.3, 26.5)	63.4 (59.9, 66.8)	13.2 (10.3, 16.2)	16.7 (13.8, 19.5)	70.5 (67.5, 73.5)	12.8 (11.1, 14.6)	18.4 (15.2, 21.6)	68.0 (64.6, 71.4)	13.6 (10.7, 16.5)	20.8 (17.7, 23.9)	66.4 (63.6, 69.3)	12.8 (11.1, 14.5)
Botswana	5.0 (3.4, 6.6)	83.8 (80.7, 87.0)	11.2 (8.6, 13.8)	7.2 (5.2, 9.2)	81.2 (77.2, 85.2)	11.6 (7.8, 15.3)	2.9 (1.0, 4.8)	86.2 (82.3, 90.2)	10.8 (7.7, 14.0)	2.7 (0.1, 5.3)	83.9 (76.1, 91.8)	13.3 (6.6, 20.1)	5.4 (3.6, 7.2)	83.8 (80.9, 86.7)	10.8 (8.3, 13.4)
Ghana	13.3 (11.3, 15.4)	62.7 (59.6, 65.9)	24.0 (21.8, 26.1)	13.3 (11.0, 15.7)	62.7 (59.3, 66.1)	24.0 (21.6, 26.4)	12.9 (10.6, 15.1)	63.1 (59.1, 67.1)	24.0 (21.1, 26.9)	12.4 (9.5, 15.3)	59.8 (55.2, 64.4)	27.8 (23.8, 31.8)	13.8 (11.6, 15.9)	64.2 (61.1, 67.4)	22.0 (20.2, 23.8)
Kenya	–	–	–	–	–	–	–	–	–	–	–	–	–	–	–
Mozambique	5.7 (3, 8.4)	74.6 (65.9, 83.4)	19.7 (12.5, 26.9)	6.2 (2.0, 10.5)	74.7 (65.8, 83.6)	19.1 (11.5, 26.7)	5.5 (2.1, 8.9)	73.8 (63.5, 84.2)	20.7 (11.8, 29.5)	8.1 (3.1, 13.1)	64.0 (50.0, 78.0)	27.9 (15.8, 40.0)	4.8 (2.4, 7.2)	78.5 (72.1, 85.0)	16.7 (11.7, 21.7)
Namibia	12.7 (9.9, 15.6)	67.2 (62.9, 71.6)	20.0 (16.4, 23.7)	13.4 (10.2, 16.6)	67.7 (63.1, 72.2)	18.9 (15.0, 22.9)	12.0 (9.0, 14.9)	67.3 (62.5, 72.1)	20.7 (16.6, 24.8)	14.3 (9.1, 19.6)	63.6 (57.0, 70.3)	22.0 (16.7, 27.3)	12.1 (9.7, 14.5)	68.7 (63.9, 73.4)	19.2 (15.5, 23.0)
Senegal	14.6 (11.0, 18.2)	75.4 (70.8, 80.0)	10.1 (7.4, 12.7)	13.6 (9.8, 17.4)	76.8 (71.9, 81.7)	9.6 (7.2, 12.0)	15.5 (11.1, 19.9)	74.1 (69.4, 78.7)	10.4 (7.1, 13.8)	14.4 (9.8, 18.9)	73.9 (68.4, 79.4)	11.7 (6.8, 16.6)	14.7 (10.8, 18.6)	76.2 (71.5, 81.0)	9.1 (7.4, 10.7)
Swaziland	4.9 (3.1, 6.7)	90.0 (88.0, 92.0)	5.1 (3.8, 6.3)	7.3 (4.3, 10.2)	88.7 (85.2, 92.2)	4.1 (2.8, 5.4)	3.4 (1.6, 5.2)	90.9 (88.7, 93.2)	5.6 (3.7, 7.5)	5.4 (2.7, 8.0)	88.3 (85.0, 91.5)	6.4 (4.8, 8.0)	4.8 (3.0, 6.5)	90.6 (88.7, 92.4)	4.7 (3.0, 6.3)
Tanzania	15.2 (12.2, 18.3)	57.6 (52.2, 63.0)	27.2 (22.8, 31.6)	16.4 (12.5, 20.2)	60.0 (53.7, 66.3)	23.6 (18.8, 28.4)	13.8 (10.9, 16.8)	55.7 (50.5, 61.0)	30.4 (26.1, 34.8)	18.1 (14.3, 22.0)	50.7 (43.6, 57.7)	31.2 (25.1, 37.2)	12.8 (10.2, 15.4)	63.3 (57.6, 69.0)	23.9 (19.3, 28.4)
Uganda	1.6 (1.0, 2.2)	83.4 (81.5, 85.3)	15.0 (12.9, 17.1)	1.9 (0.9, 2.8)	86.9 (84.8, 89.1)	11.2 (8.7, 13.7)	1.4 (0.6, 2.3)	80.1 (77.4, 82.7)	18.5 (15.7, 21.3)	1.4 (0.4, 2.4)	84.0 (80.1, 87.9)	14.6 (10.9, 18.3)	1.7 (1.1, 2.2)	83.3 (81.1, 85.5)	15.1 (12.7, 17.4)
Zambia	9.2 (6.0, 12.5)	70.6 (66.1, 75.2)	20.2 (17.3, 23.1)	8.9 (4.5, 13.3)	71.8 (66.0, 77.5)	19.3 (14.9, 23.8)	9.5 (5.4, 13.7)	70.1 (64.7, 75.5)	20.4 (16.5, 24.3)	12.0 (7.9, 16.2)	68.5 (62.3, 74.8)	19.5 (15.4, 23.5)	7.7 (4.7, 10.8)	71.7 (67.4, 76.1)	20.5 (17.2, 23.9)
Zimbabwe	4.6 (3.6, 5.7)	90.4 (88.6, 92.2)	5.0 (4.0, 6.0)	6.0 (4.4, 7.6)	89.4 (87.3, 91.6)	4.6 (3.6, 5.6)	3.4 (2.4, 4.3)	91.3 (89.3, 93.3)	5.3 (3.8, 6.8)	5.2 (2.7, 7.6)	90.0 (86.8, 93.3)	4.8 (2.9, 6.6)	4.5 (3.6, 5.5)	90.5 (88.7, 92.2)	5.0 (4, 6)
**Pooled estimates**	**9.6 (6.3,**	**74.9**	**15.3**	**10.6 (6.8,**	**75 (68.0,**	**14.2 (9.9,**	**8.6 (5.6,**	**75.0**	**16.1 (11.5,**	**10.1 (6.1,**	**72.5**	**17.0**	**9.2 (6.2,**	**76.2**	**14.4**

	**12.8)**	**(68.2, 81.6)**	**(10.9, 19.8)**	**14.5)**	**81.9)**	**18.6)**	**11.6)**	**(67.9, 82.0)**	**20.7)**	**14.1)**	**(64.8, 80.3)**	**(12.0, 22.0)**	**12.2)**	**(69.8, 82.6)**	**(10.3, 18.5)**
**I^2 (%)**	**97.6**	**98.2**	**98.0**	**96.6**	**97.5**	**97.3**	**96.4**	**97.8**	**96.8**	**95.7**	**96.5**	**95.7**	**97.4**	**98.1**	**97.7**
**European Region**															
Macedonia	3.0 (1.8, 4.2)	90.9 (89.4, 92.3)	6.1 (4.2, 8.0)	4.2 (2.3, 6.2)	90.0 (87.5, 92.6)	5.7 (3.5, 7.9)	1.6 (0.5, 2.8)	92.1 (90.1, 94.1)	6.2 (3.9, 8.5)	3.7 (1.5, 6.0)	90.1 (88.5, 91.7)	6.2 (4.1, 8.3)	2.6 (1.4, 3.7)	91.3 (89.2, 93.4)	6.1 (3.6, 8.6)
**Pooled estimates**	–	–	–	–	–	–	–	–	–	–	–	–	–	–	–
**I^2 (%)**	–	–	–	–	–	–	–	–	–	–	–	–	–	–	–
**America Region**															
Argentina	7.3 (6.7, 8.0)	79.7 (78.3, 81.1)	12.9 (11.6, 14.3)	10.3 (8.9, 11.7)	80.6 (78.8, 82.4)	9.1 (7.9, 10.2)	4.6 (3.9, 5.2)	79.0 (77.0, 81.0)	16.4 (14.4, 18.4)	7.3 (5.9, 8.7)	79.9 (78.0, 81.8)	12.8 (10.8, 14.8)	7.4 (6.5, 8.2)	79.7 (78.0, 81.4)	13.0 (11.6, 14.4)
Bahamas	5.9 (4.4, 7.3)	84.3 (80.7, 87.8)	9.9 (7.3, 12.4)	8.1 (6.2, 10.0)	82.8 (78.3, 87.3)	9.1 (5.7, 12.5)	3.9 (2.3, 5.5)	85.5 (81.8, 89.1)	10.6 (7.8, 13.5)	6.1 (4.5, 7.8)	83.2 (78.6, 87.7)	10.7 (7.0, 14.4)	5.6 (3.6, 7.6)	85.5 (81.0, 89.9)	9.0 (5.8, 12.1)
Barbados	3.6 (2.7, 4.6)	89.7 (88.0, 91.3)	6.7 (5.0, 8.4)	4.4 (2.9, 5.9)	88.5 (85.8, 91.1)	7.1 (4.7, 9.6)	2.8 (1.5, 4.1)	90.9 (88.2, 93.6)	6.3 (3.7, 8.9)	3.4 (1.2, 5.5)	87.1 (83.6, 90.6)	9.5 (4.9, 14.1)	3.7 (2.5, 4.9)	90.5 (88.5, 92.5)	5.8 (4.0, 7.7)
Belize	2.0 (1.2, 2.8)	84.3 (81.0, 87.7)	13.6 (10.6, 16.7)	2.5 (1.3, 3.7)	85.5 (81.3, 89.7)	12 .0 (8.2, 15.8)	1.6 (0.5, 2.6)	83.2 (79.2, 87.2)	15.2 (11.8, 18.6)	1.9 (0.5, 3.2)	81.3 (75.7, 86.9)	16.9 (11.8, 21.9)	2.2 (1.5, 2.9)	87.1 (83.9, 90.2)	10.7 (7.8, 13.7)
Bolivia	7.9 (6.6, 9.2)	84.7 (83.3, 86.1)	7.4 (6.0, 8.8)	9.7 (7.8, 11.7)	83.4 (81.2, 85.6)	6.9 (5.3, 8.4)	6.1 (4.7, 7.6)	86.3 (84.3, 88.3)	7.6 (5.8, 9.4)	9.2 (7.4, 10.9)	82.7 (80.7, 84.7)	8.1 (6.7, 9.5)	7.5 (6.1, 8.9)	85.4 (83.5, 87.3)	7.1 (5.3, 9.0)
Cayman	4.6 (3.4, 5.9)	89.6 (87.8, 91.4)	5.8 (4.4, 7.1)	5.9 (3.9, 7.9)	88.5 (85.9, 91.2)	5.5 (3.7, 7.4)	3.3 (1.8, 4.7)	90.7 (88.3, 93.0)	6.1 (4.2, 8.0)	5.4 (3.4, 7.4)	87.5 (84.7, 90.4)	7.0 (4.9, 9.2)	4 .0 (2.4, 5.7)	91.0 (88.7, 93.3)	4.9 (3.2, 6.6)
Costa Rica	1.8 (1.2, 2.3)	81.4 (79.4, 83.4)	16.8 (15.1, 18.5)	2.4 (1.3, 3.5)	83.9 (81.1, 86.7)	13.7 (11.6, 15.8)	1.2 (0.4, 1.9)	78.9 (76.5, 81.3)	19.9 (17.9, 22.0)	1.6 (0.4, 2.8)	81.0 (78.3, 83.7)	17.4 (15.1, 19.7)	1.9 (1.2, 2.5)	81.7 (79.0, 84.3)	16.5 (14.3, 18.7)
Curaçao	3.8 (2.9, 4.6)	91.0 (89.5, 92.5)	5.2 (3.9, 6.5)	4.5 (2.8, 6.3)	90.7 (88.3, 93.1)	4.7 (3.3, 6.2)	2.8 (1.8, 3.8)	91.5 (89.4, 93.6)	5.7 (3.7, 7.7)	3.8 (2.2, 5.4)	92 (89.1, 94.9)	4.2 (2.2, 6.2)	3.8 (2.7, 4.9)	90.6 (88.8, 92.4)	5.6 (4.0, 7.3)
Ecuador	12.8 (11.0, 14.7)	87.1 (85.3, 88.9)	0.1 (0.0, 0.1)	14.9 (12.7, 17.1)	85.0 (82.9, 87.2)	0.1 (0.0, 0.2)	10.1 (8.5, 11.8)	89.7 (88.1, 91.4)	0.1 (0.0, 0.3)	13.5 (11.5, 15.5)	86.4 (84.4, 88.3)	0.2 (0.0, 0.3)	12.0 (9.9, 14.2)	88.0 (85.8, 90.1)	–
El Salvador	2.7 (1.8, 3.7)	85.5 (84.0, 86.9)	11.8 (10.2, 13.4)	2.9 (1.8, 3.9)	87.7 (85.4, 90.1)	9.4 (7.1, 11.6)	1.9 (0.9, 2.9)	83.8 (81.5, 86.2)	14.2 (11.6, 16.8)	3.4 (1.7, 5.2)	86.3 (84.4, 88.3)	10.2 (7.5, 13.0)	2.4 (1.4, 3.5)	85.1 (83.1, 87.1)	12.5 (10.4, 14.5)

Grenada	3.9 (2.4, 5.3)	86.5 (83.5, 89.4)	9.6 (7.1, 12.2)	5.7 (3.2, 8.3)	84.0 (79.6, 88.4)	10.3 (6.4, 14.1)	2.4 (1.0, 3.7)	88.4 (85.3, 91.6)	9.2 (6.1, 12.3)	2.8 (1.4, 4.1)	88.1 (85.1, 91.0)	9.2 (6.3, 12.1)	4.7 (2.7, 6.7)	85.4 (81.0, 89.8)	10 (6.6, 13.3)
Guatemala	5.8 (3.5, 8.0)	75.7 (73.3, 78.1)	18.5 (14.8, 22.2)	6.8 (2.9, 10.7)	77.8 (74.7, 81.0)	15.4 (12.2, 18.6)	4.8 (3.2, 6.5)	73.2 (69.4, 77.1)	22.0 (17.7, 26.2)	4.2 (3.1, 5.3)	79.2 (75.7, 82.7)	16.6 (13.3, 19.9)	6.6 (3.5, 9.7)	73.9 (69.9, 77.9)	19.5 (13.8, 25.3)
Guyana	4.2 (3.0, 5.5)	88.1 (85.7, 90.6)	7.6 (5.8, 9.4)	5.2 (3.3, 7.0)	86.7 (83.7, 89.8)	8.1 (6.1, 10.1)	3.3 (2.2, 4.5)	89.9 (87.7, 92.2)	6.7 (4.6, 8.9)	3.2 (1.6, 4.9)	90.3 (87.5, 93.0)	6.5 (4.6, 8.4)	4.7 (3.3, 6.0)	87.3 (84.5, 90.1)	8.0 (5.8, 10.3)
Honduras	4.1 (3.0, 5.3)	80.2 (77.9, 82.5)	15.7 (13.4, 17.9)	4.7 (3.1, 6.4)	82.9 (80.2, 85.7)	12.4 (10.0, 14.7)	3.4 (2.0, 4.8)	78.1 (74.2, 81.9)	18.5 (14.9, 22.1)	5.3 (3.9, 6.7)	78.6 (74.7, 82.6)	16.1 (12.5, 19.6)	3.1 (1.9, 4.3)	81.6 (79.1, 84.2)	15.3 (12.7, 17.9)
Peru	4.3 (3.3, 5.3)	86.4 (84.7, 88.1)	9.3 (7.8, 10.9)	4.7 (3.0, 6.5)	86.9 (84.5, 89.4)	8.3 (6.0, 10.7)	3.8 (2.5, 5.1)	86.0 (83.3, 88.7)	10.2 (7.9, 12.5)	3.1 (1.6, 4.5)	87.6 (84.4, 90.8)	9.3 (6.2, 12.5)	4.7 (3.5, 5.8)	86.0 (83.9, 88.0)	9.4 (7.7, 11.0)
Saint Kitts and Nevis	3.9 (2.8, 5.0)	86.7 (84.9, 88.5)	9.4 (7.9, 11.0)	5.0 (3.2, 6.7)	85.1 (82.2, 88.0)	9.9 (7.5, 12.3)	2.9 (1.7, 4.1)	88.3 (86.0, 90.5)	8.9 (6.9, 10.8)	3.4 (1.4, 5.5)	85.6 (81.9, 89.2)	11.0 (7.8, 14.2)	4.0 (2.8, 5.3)	87.0 (84.9, 89.1)	9.0 (7.2, 10.7)
Saint Lucia	3.2 (2.2, 4.2)	90.5 (88.5, 92.5)	6.3 (4.2, 8.4)	4.7 (2.9, 6.6)	88.8 (86.0, 91.5)	6.5 (4.3, 8.8)	2.1 (1.1, 3.0)	91.8 (88.7, 94.9)	6.1 (3.2, 9.1)	2.4 (1.2, 3.5)	89.0 (87.0, 91.0)	8.7 (6.6, 10.8)	3.8 (2.4, 5.2)	91.6 (89.6, 93.5)	4.6 (3.0, 6.2)
Saint Vincent and Grenadines	4.1 (2.7, 5.6)	81.9 (78.5, 85.4)	13.9 (11.1, 16.8)	5.1 (2.7, 7.5)	83.0 (79.2, 86.8)	11.9 (9.0, 14.9)	3.2 (1.8, 4.5)	81.2 (77.5, 84.8)	15.6 (11.7, 19.5)	3.9 (2.7, 5.2)	82.9 (78.1, 87.7)	13.2 (8.9, 17.4)	4.4 (2.5, 6.3)	80.9 (77.4, 84.4)	14.7 (11.6, 17.9)
Trinidad and Tobago	3.9 (2.5, 5.2)	92.1 (90.2, 94.0)	4.0 (2.8, 5.2)	3.9 (2.6, 5.3)	92.1 (89.9, 94.3)	4.0 (2.3, 5.6)	3.7 (2.0, 5.4)	92.2 (89.7, 94.6)	4.1 (2.2, 6.0)	2.9 (1.7, 4.1)	92.3 (90.1, 94.5)	4.8 (3.3, 6.3)	4.7 (3.1, 6.3)	91.9 (89.7, 94.2)	3.4 (1.8, 5.0)
Uruguay	2.6 (1.9, 3.2)	77.2 (75.4, 79.0)	20.3 (18.5, 22.0)	3.7 (2.4, 5.0)	80.5 (78.0, 83.0)	15.8 (13.7, 18.0)	1.6 (0.8, 2.3)	74.4 (71.9, 76.8)	24.1 (21.5, 26.7)	2.7 (1.6, 3.8)	78.1 (74.8, 81.4)	19.2 (16.1, 22.4)	2.5 (1.8, 3.2)	76.9 (74.9, 78.8)	20.6 (18.8, 22.5)
Venezuela	2.0 (1.6, 2.5)	82.8 (80.4, 85.1)	15.2 (13.0, 17.4)	2.1 (1.4, 2.9)	83.3 (79.5, 87.0)	14.6 (11.2, 18.1)	2.0 (1.4, 2.6)	82.3 (80.3, 84.4)	15.7 (13.7, 17.6)	2.4 (1.8, 3.1)	81.3 (77.8, 84.8)	16.3 (13.3, 19.3)	1.4 (0.9, 1.9)	85.2 (82.7, 87.6)	13.5 (11.0, 16.0)
**Pooled estimates**	**4.4 (3.5, 5.3)**	**85.1 (83.1, 87.0)**	**10.4 (7.2, 13.6)**	**5.5 (4.3, 6.7)**	**85.2 (83.6, 86.8)**	**9.2 (6.4, 12.0)**	**3.3 (2.6, 4.0)**	**85.1 (82.7, 87.5)**	**11.5 (7.8, 15.2)**	**4.3 (3.3, 5.3)**	**85.4 (81.6, 89.2)**	**10.8 (7.6, 13.9)**	**4.4 (3.5, 5.3)**	**85.7 (81.4, 89.9)**	**10.5 (8.3, 12.7)**
**I^2 (%)**	**95.0**	**95.4**	**99.3**	**92.7**	**86.9**	**98.6**	**89.0**	**94.7**	**99.0**	**90.9**	**91.1**	**98.5**	**93.5**	**96.0**	**95.5**
**Eastern Mediterranean Region**															

Afghanistan	25.5 (19.5, 31.6)	62.0 (58.3, 65.7)	12.5 (8.2, 16.7)	31.5 (23.6, 39.5)	59.7 (54.0, 65.5)	8.7 (2.8, 14.7)	18.0 (12.8, 23.2)	64.8 (62.1, 67.6)	17.1 (12.0, 22.3)	23.0 (15.4, 30.7)	65.0 (57.6, 72.5)	11.9 (6.8, 17.0)	26.5 (19.7, 33.2)	60.9 (55.3, 66.5)	12.7 (7.8, 17.5)
Djibouti	10.6 (7.9, 13.2)	56.8 (53.4, 60.2)	32.6 (29.5, 35.7)	11.0 (7.5, 14.6)	56.8 (52.5, 61.1)	32.2 (28.2, 36.1)	9.9 (5.6, 14.2)	56.7 (50.3, 63.2)	33.4 (27.9, 38.8)	12.6 (6.4, 18.8)	63.6 (50.6, 76.5)	23.9 (15.7, 32.0)	10.2 (7.7, 12.7)	55.5 (51.4, 59.6)	34.3 (30.4, 38.3)
Egypt	37.8 (32.9, 42.8)	53.2 (47.9, 58.6)	8.9 (6.4, 11.5)	40.3 (33.6, 47.0)	49.9 (43.4, 56.4)	9.8 (7.0, 12.7)	35.7 (28.9, 42.4)	56.3 (49.6, 63.0)	8.0 (5.1, 11.0)	38.8 (33.1, 44.5)	52.6 (46.9, 58.4)	8.6 (5.7, 11.4)	36.8 (27.9, 45.7)	53.9 (45.3, 62.4)	9.3 (4.4, 14.3)
Iraq	21.5 (18.4, 24.5)	67.8 (63.9, 71.7)	10.8 (8.5, 13.0)	26.2 (22.4, 30.0)	63.0 (57.7, 68.3)	10.8 (7.5, 14.1)	15.8 (13.2, 18.5)	73.4 (69.7, 77.1)	10.7 (8.0, 13.5)	20.0 (16.1, 24.0)	65.9 (60.5, 71.3)	14.1 (10.2, 18.0)	22.3 (18.8, 25.8)	68.9 (64.0, 73.7)	8.8 (6.7, 11.0)
Jordan	23.5 (19.7, 27.3)	69.4 (65.8, 72.9)	7.1 (5.9, 8.4)	28.8 (25.9, 31.6)	64.9 (61.7, 68.2)	6.3 (4.8, 7.8)	18.5 (14.4, 22.6)	73.5 (68.9, 78.2)	7.9 (6.4, 9.5)	18.5 (11.9, 25.0)	76.7 (69.9, 83.5)	4.8 (1.6, 8.0)	24.2 (20.3, 28.0)	68.4 (64.7, 72.1)	7.5 (6.1, 8.8)
Kuwait	14.9 (10.2, 19.6)	79.2 (74.1, 84.3)	5.9 (4.3, 7.5)	20.0 (14.9, 25.0)	74.7 (68.8, 80.7)	5.3 (3.4, 7.3)	9.5 (7.8, 11.1)	83.9 (80.8, 87.1)	6.6 (4.3, 8.9)	14.6 (7.8, 21.3)	80.3 (73.0, 87.7)	5.1 (3.2, 7.0)	15 (10.3, 19.7)	78.8 (73.4, 84.1)	6.2 (4.2, 8.2)
Lebanon	9.5 (7.8, 11.2)	83.5 (80.1, 86.9)	7.0 (4.4, 9.6)	12.3 (9.2, 15.4)	80.5 (77.8, 83.3)	7.1 (4.0, 10.3)	7 .0 (3.7, 10.3)	86.1 (80.7, 91.5)	6.9 (4.5, 9.3)	8.5 (6.3, 10.7)	85.0 (81.6, 88.4)	6.5 (3.7, 9.3)	10.3 (8.1, 12.5)	82.4 (78.2, 86.6)	7.4 (4.6, 10.1)
Libya	–	–	–	–	–	–	–	–	–	–	–	–	–	–	–
Morocco	31.6 (26.6, 36.5)	54.8 (50.6, 59.1)	13.6 (11.4, 15.8)	35.6 (30.2, 41.0)	52.0 (46.7, 57.3)	12.4 (9.8, 15.1)	26.8 (22.2, 31.4)	58.3 (54.6, 62.1)	14.9 (11.8, 18.0)	30.0 (24.9, 35.1)	55.0 (49.9, 60.1)	15.0 (11.4, 18.6)	32.7 (27.5, 38.0)	54.7 (49.8, 59.6)	12.6 (10.0, 15.1)
Oman	15.4 (11.1, 19.6)	77.1 (72.7, 81.5)	7.5 (6.3, 8.8)	22.6 (18.1, 27.0)	70.9 (66.2, 75.5)	6.6 (5.4, 7.8)	8.7 (5.4, 11.9)	82.8 (79.1, 86.4)	8.6 (6.8, 10.4)	12.0 (6.7, 17.3)	78.5 (72.4, 84.5)	9.5 (6.7, 12.4)	16.4 (11.9, 20.8)	76.7 (71.9, 81.5)	6.9 (5.7, 8.2)
Pakistan	16.2 (12.4, 20.1)	80.0 (76.4, 83.6)	3.7 (2.0, 5.5)	22.3 (17.6, 27.0)	73.7 (69.3, 78.0)	4.1 (2.0, 6.1)	7.0 (5.0, 9.1)	89.7 (88.0, 91.3)	3.3 (2.9, 3.7)	13.6 (8.7, 18.5)	82.5 (77.0, 88.1)	3.9 (0.9, 6.8)	17.0 (12.9, 21.2)	79.2 (75.5, 83.0)	3.7 (2.2, 5.3)
Qatar	19.2 (15.2, 23.3)	61.6 (58.1, 65.1)	19.2 (16.4, 22.0)	26.0 (19.3, 32.6)	59.6 (54.2, 64.9)	14.5 (11.4, 17.6)	12.9 (7.5, 18.3)	63.4 (58.4, 68.5)	23.6 (18.9, 28.4)	15.6 (12.8, 18.4)	61.8 (58.3, 65.4)	22.5 (18.6, 26.5)	23.2 (15.8, 30.6)	61.3 (54.9, 67.7)	15.5 (11.8, 19.3)
Sudan	10.2 (7.1, 13.4)	77.2 (73.4, 81.0)	12.6 (9.8, 15.4)	13.2 (8.7, 17.7)	76.6 (71.9, 81.4)	10.2 (7.4, 12.9)	6.9 (4.2, 9.6)	78.0 (71.0, 85.0)	15.1 (9.1, 21.2)	6.8 (0.9, 12.7)	79.6 (70.5, 88.7)	13.6 (8.1, 19.1)	11.2 (7.8, 14.7)	76.5 (73.1, 79.9)	12.3 (9.5, 15.0)
Syrian Arab Republic	33.5 (30.5, 36.6)	61.7 (58.5, 64.9)	4.8 (3.8, 5.7)	38.2 (35.5, 40.9)	57.7 (54.5, 61.0)	4.1 (3.0, 5.1)	28.6 (24.9, 32.4)	65.9 (62.4, 69.4)	5.5 (4.5, 6.5)	33.5 (29.8, 37.3)	61.1 (57.7, 64.6)	5.3 (3.8, 6.9)	33.5 (30.1, 36.9)	62.2 (58.4, 65.9)	4.3 (3.0, 5.6)

Tunisia	11.5 (9.7, 13.3)	80.2 (77.6, 82.8)	8.3 (6.6, 10.0)	15.4 (12.4, 18.3)	77.1 (73.7, 80.5)	7.6 (5.6, 9.5)	7.7 (5.8, 9.5)	83.1 (79.9, 86.2)	9.2 (6.8, 11.7)	9.3 (6.7, 12.0)	82.7 (79.0, 86.3)	8.0 (5.4, 10.6)	13.2 (11.6, 14.8)	78.2 (75.9, 80.5)	8.6 (7.0, 10.2)
United Arab Emirates	14.6 (10.9, 18.4)	76.2 (72.1, 80.3)	9.2 (7.4, 11.0)	23.4 (20.2, 26.5)	67.7 (62.4, 73.1)	8.9 (5.8, 12.0)	8.7 (6.6, 10.8)	81.9 (78.8, 84.9)	9.4 (7.8, 11.0)	13.4 (9.4, 17.5)	76.9 (71.8, 82.1)	9.6 (7.8, 11.4)	15.2 (10.9, 19.4)	75.9 (71.3, 80.4)	9 .0 (6.9, 11.0)
UNRWA	30.3 (28.2, 32.4)	61.9 (60.0, 63.9)	7.7 (6.7, 8.8)	37.8 (36.5, 39.2)	55.8 (54.2, 57.4)	6.3 (5.5, 7.2)	23.2 (21.8, 24.7)	67.8 (66.0, 69.5)	9.0 (8.2, 9.8)	29.0 (26.3, 31.6)	61.8 (59.1, 64.4)	9.2 (7.7, 10.8)	31.2 (28.9, 33.4)	62.0 (59.7, 64.3)	6.8 (5.8, 7.8)
Yemen	35.7 (26.1, 45.3)	55.4 (46.5, 64.3)	8.9 (6.4, 11.5)	39.5 (27.4, 51.6)	50.0 (39.8, 60.1)	10.5 (7.4, 13.6)	29.0 (14.0, 43.9)	64.7 (50.0, 79.3)	6.4 (1.7, 11.0)	33.5 (19.7, 47.4)	58.7 (46.7, 70.6)	7.8 (3.6, 12.0)	37.0 (28.7, 45.4)	53.3 (44.9, 61.7)	9.7 (5.4, 13.9)
**Pooled estimates**	**21.0 (16.5, 25.6)**	**68.2 (63.5, 73.0)**	**10.4 (8.2, 12.6)**	**25.9 (20.6, 31.2)**	**64.3 (59.3, 69.4)**	**9.4 (7.5, 11.4)**	**15.5 (11.7, 19.3)**	**72.6 (67.1, 78.1)**	**10.8 (8.7, 12.9)**	**19.3 (14.6, 24.0)**	**70.0 (64.5, 75.4)**	**9.9 (7.9, 11.9)**	**21.8 (17.4, 26.1)**	**67.8 (63.3, 72.4)**	**10.0 (8.0, 12.0)**
**I^2 (%)**	**97.1**	**96.6**	**96.3**	**97.4**	**96.4**	**93.8**	**96.7**	**97.8**	**96.7**	**95.5**	**95.1**	**88.2**	**96.4**	**95.3**	**94.4**
**Southeast Asia Region**															
Bangladesh	12.2 (10.3, 14.1)	86.9 (84.8, 88.9)	0.9 (0.3, 1.5)	14.6 (12.4, 16.8)	84.2 (81.9, 86.6)	1.2 (0.7, 1.7)	8.2 (5.7, 10.7)	91.3 (88.9, 93.8)	0.4 (0.1, 0.8)	10.6 (8.2, 12.9)	88.0 (85.4, 90.6)	1.4 (0.7, 2.1)	12.9 (10.2, 15.6)	86.4 (83.5, 89.3)	0.7 (0.0, 1.5)
India	4.4 (3.5, 5.3)	94.0 (92.9, 95.1)	1.6 (1.2, 2.0)	4.8 (3.8, 5.8)	93.7 (92.3, 95.1)	1.5 (0.9, 2.1)	3.9 (2.8, 4.9)	94.4 (93.1, 95.7)	1.7 (1.1, 2.3)	4.0 (3.1, 5.0)	93.9 (92.6, 95.2)	2.1 (1.4, 2.8)	4.6 (3.5, 5.8)	94 (92.6, 95.5)	1.3 (0.9, 1.8)
Indonesia	2.5 (1.9, 3.0)	94.2 (93.2, 95.1)	3.4 (2.7, 4.0)	3.7 (2.9, 4.4)	93.0 (91.7, 94.4)	3.3 (2.4, 4.1)	1.3 (0.8, 1.8)	95.3 (94.3, 96.2)	3.5 (2.7, 4.3)	2.5 (1.7, 3.2)	93.7 (92.5, 94.9)	3.8 (3.1, 4.6)	2.4 (1.8, 3.1)	94.7 (93.7, 95.7)	2.9 (2.3, 3.5)
Thailand	5.5 (4.3, 6.7)	90.2 (88.3, 92.0)	4.4 (3.1, 5.6)	8.6 (6.3, 11.0)	86.4 (83.8, 89.0)	5.0 (3.4, 6.5)	2.4 (1.4, 3.4)	93.8 (92.0, 95.6)	3.8 (2.6, 4.9)	5.9 (4.3, 7.5)	89.0 (86.5, 91.5)	5.1 (3.1, 7.0)	5.2 (3.6, 6.7)	91.1 (89.0, 93.1)	3.8 (2.6, 5.0)
Timor-Leste	18.4 (15.2, 21.6)	75.8 (72.6, 78.9)	5.8 (4.3, 7.4)	19.7 (16.1, 23.4)	75.9 (72, 79.8)	4.4 (2.8, 6.0)	15.8 (12.9, 18.7)	77.0 (73.3, 80.6)	7.3 (5.3, 9.2)	22.5 (19.4, 25.7)	70.0 (66.6, 73.4)	7.5 (5.1, 9.8)	17.0 (13.0, 21.0)	77.7 (73.5, 81.9)	5.3 (3.9, 6.7)
**Pooled estimates**	**8.3 (4.9, 11.7)**	**88.4 (84.1, 92.8)**	**3.1 (1.7, 4.4)**	**10.0 (5.9, 14)**	**86.9 (81.9, 91.8)**	**2.9 (1.7, 4.2)**	**5.9 (3.1, 8.8)**	**90.8 (87.1, 94.4)**	**3.2 (1.4, 4.9)**	**8.8 (4.8, 12.9)**	**7.1 (81.3, 92.9)**	**3.6 (2.1, 5.1)**	**7.9 (4.6, 11.3)**	**89.2 (85.4, 93.1)**	**2.7 (1.4, 4.0)**
**I^2 (%)**	**97.8**	**97.5**	**94.4**	**97.4**	**96.8**	**91.0**	**96.9**	**95.8**	**96.4**	**97.8**	**97.9**	**91.3**	**96.3**	**95.4**	**93.3**
**Western Pacific Region**															
Brunei	2.2 (1.5,	87.7	10.1 (8.6,	2.7 (1.7,	88.0	9.3 (7.0,	1.6 (0.9,	87.6	10.7 (8.7,	2.3 (1.2,	86.0	11.7 (8.4,	2.1 (1.3,	88.5	9.4 (7.6,

Darussalam	2.8)	(86.1, 89.4)	11.6)	3.7)	(85.3, 90.6)	11.6)	2.4)	(85.5, 89.7)	12.8)	3.5)	(82.5, 89.5)	15.0)	2.9)	(86.5, 90.5)	11.1)
Cambodia	4.7 (3.4, 6.1)	93.9 (92.5, 95.3)	1.4 (0.8, 2.0)	5.3 (3.8, 6.8)	93.8 (92.1, 95.4)	0.9 (0.0, 1.9)	4.1 (1.9, 6.2)	94.1 (92.0, 96.3)	1.8 (1.0, 2.6)	6.1 (3.8, 8.5)	92.9 (90.9, 95.0)	0.9 (0.0, 1.8)	4.2 (2.6, 5.8)	94.2 (92.4, 96.1)	1.6 (0.8, 2.3)
China	2.7 (2.1, 3.3)	94.0 (93.3, 94.7)	3.3 (2.7, 3.9)	3.4 (2.5, 4.3)	93.0 (92.2, 93.9)	3.5 (2.8, 4.3)	1.8 (1.2, 2.5)	95.1 (94.0, 96.1)	3.1 (2.2, 4.0)	2.8 (1.9, 3.8)	93.0 (91.6, 94.4)	4.1 (3.0, 5.3)	2.6 (1.8, 3.3)	94.8 (94.1, 95.5)	2.6 (2.2, 3.1)
Cook	12.6 (8.4, 16.7)	77.1 (73.5, 80.7)	10.3 (8.2, 12.5)	14.9 (8.5, 21.3)	75.8 (69.5, 82.0)	9.3 (4.9, 13.7)	10.0 (5.5, 14.6)	79.0 (73.7, 84.2)	11.0 (7.1, 14.9)	5.7 (0.0, 13.0)	86.8 (79.1, 94.5)	7.5 (4.7, 10.2)	14.1 (9.0, 19.2)	74.9 (70.3, 79.6)	11.0 (8.2, 13.8)
Kiribati	19.4 (16.0, 22.8)	66.6 (61.9, 71.3)	14.0 (11.3, 16.6)	24.8 (19.5, 30.1)	59.1 (52.6, 65.7)	16.1 (12.1, 20.0)	14.9 (12.1, 17.7)	72.8 (68.5, 77.1)	12.2 (9.4, 15.1)	21.6 (17.2, 26.0)	60.8 (55.3, 66.3)	17.6 (13.3, 22.0)	18.5 (14.7, 22.4)	69.0 (63.4, 74.5)	12.5 (9.8, 15.2)
Laos	2.3 (1.1, 3.5)	94.1 (92.8, 95.4)	3.6 (2.5, 4.7)	1.8 (0.4, 3.1)	93.6 (91.4, 95.7)	4.7 (2.9, 6.4)	2.8 (1.3, 4.3)	94.5 (93.0, 96.1)	2.7 (1.4, 4.1)	2.9 (0.3, 5.6)	92.4 (87.1, 97.7)	4.7 (0.6, 8.8)	2.2 (1.0, 3.4)	94.3 (93.0, 95.6)	3.5 (2.1, 4.8)
Malaysia	2.8 (2.4, 3.3)	85.9 (84.8, 87.1)	11.2 (10.3, 12.2)	3.9 (3.3, 4.5)	85.0 (83.6, 86.4)	11.1 (10.0, 12.3)	1.8 (1.3, 2.4)	86.9 (85.3, 88.4)	11.3 (9.9, 12.7)	3.4 (2.6, 4.1)	83.0 (81.2, 84.9)	13.6 (11.9, 15.3)	2.6 (2.0, 3.1)	87.4 (86.2, 88.7)	10.0 (9.0, 11.0)
Mongolia	5.5 (4.5, 6.5)	83.8 (82.2, 85.4)	10.8 (9.3, 12.2)	7.0 (5.5, 8.5)	81.3 (79.4, 83.3)	11.7 (9.8, 13.6)	4.0 (3.0, 5.0)	86.2 (84.1, 88.3)	9.8 (8.1, 11.5)	5.9 (4.5, 7.4)	80.7 (78.2, 83.3)	13.3 (11.1, 15.6)	5.1 (4.0, 6.2)	86.2 (84.3, 88.1)	8.7 (7.1, 10.3)
Nauru	13.2 (9.4, 16.9)	52.6 (47.1, 58.0)	34.3 (29.1, 39.5)	17.7 (11.4, 24.1)	46.1 (37.7, 54.4)	36.2 (28.2, 44.3)	9.3 (5.1, 13.5)	57.6 (50.4, 64.7)	33.2 (26.3, 40.0)	12.9 (7.4, 18.4)	49.2 (40.8, 57.6)	37.9 (29.7, 46.0)	13.4 (8.3, 18.4)	55.5 (48.3, 62.6)	31.2 (24.5, 37.9)
Philippines	5.8 (3.2, 8.3)	85.9 (83.6, 88.2)	8.3 (7.1, 9.5)	6.8 (4.4, 9.2)	84.7 (82.1, 87.2)	8.5 (6.9, 10.2)	4.7 (1.7, 7.8)	87.1 (84.4, 89.9)	8.1 (6.7, 9.6)	4.8 (2.3, 7.4)	86.9 (83.8, 90.0)	8.3 (6.5, 10.1)	6.3 (2.9, 9.7)	85.4 (82.6, 88.1)	8.4 (7.0, 9.7)
Samoa	17.6 (14.1, 21.2)	57.0 (52.9, 61.1)	25.3 (22.0, 28.6)	22.1 (17.1, 27.0)	54.4 (48.8, 60.1)	23.5 (20.0, 27.0)	13.5 (9.5, 17.4)	59.5 (55.9, 63.1)	27.0 (23.3, 30.7)	21.4 (16.1, 26.7)	51.8 (46.0, 57.7)	26.7 (22.9, 30.6)	16.4 (12.1, 20.7)	58.7 (54.0, 63.5)	24.8 (21.1, 28.5)
Solomon	22.9 (18.4, 27.4)	58.0 (51.0, 64.9)	19.1 (12.8, 25.3)	26.6 (22.2, 31.0)	57.5 (51.7, 63.3)	15.9 (11.2, 20.7)	19.1 (13.8, 24.3)	57.8 (46.9, 68.7)	23.1 (12.6, 33.7)	23.0 (16.0, 29.9)	58.8 (46.3, 71.3)	18.2 (6.2, 30.2)	22.9 (18.2, 27.6)	57.7 (52.0, 63.4)	19.4 (14.1, 24.6)
Tokelau	16.5 (8.0, 25.1)	69.6 (58.9, 80.3)	13.9 (5.8, 22.0)	20.9 (7.5, 34.2)	71.8 (57.4, 86.2)	7.3 (0.0, 14.6)	11.5 (1.4, 21.5)	65.5 (48.3, 82.8)	23.0 (7.0, 38.9)	18.2 (5.4, 31.0)	72.1 (57.1, 87.2)	9.7 (0.0, 19.8)	14.5 (3.1, 25.8)	66.4 (50.7, 82.1)	19.2 (5.7, 32.6)
Tonga	9.2 (7.5, 10.9)	68.1 (64.7,	22.7 (19.9,	11.5 (8.6, 14.4)	65.5 (60.5,	23.0 (18.8,	6.8 (5.3, 8.4)	70.7 (66.5,	22.5 (18.8,	10.1 (7.5, 12.8)	64.1 (58.0,	25.8 (20.4,	8.8 (7.0, 10.7)	69.6 (66.3,	21.6 (18.6,

		71.4)	25.6)		70.5)	27.3)		74.9)	26.2)		70.1)	31.2)		72.8)	24.5)
Tuvalu	7.9 (5.8, 9.9)	56.3 (52.5, 60.1)	35.8 (32.1, 39.4)	11.8 (8.4, 15.3)	52.0 (46.5, 57.5)	36.2 (30.8, 41.5)	4.2 (2.0, 6.5)	60.2 (55.0, 65.5)	35.5 (30.4, 40.7)	8.8 (6.0, 11.6)	49.8 (44.8, 54.9)	41.4 (36.4, 46.4)	6.7 (3.7, 9.7)	64.8 (59.2, 70.4)	28.5 (23.2, 33.7)
Vanuatu	19.1 (15.1, 23.2)	60.4 (52.8, 68.1)	20.4 (15.3, 25.6)	20.6 (12.8, 28.5)	59.6 (50.4, 68.8)	19.8 (13.1, 26.5)	17.5 (13.5, 21.4)	60.9 (53.8, 68.0)	21.6 (13.7, 29.6)	21.7 (15.0, 28.3)	53.8 (42.5, 65.2)	24.5 (17.2, 31.8)	16.4 (11.2, 21.5)	67.6 (59.1, 76.1)	16.0 (9.2, 22.8)
Vietnam	3.4 (2.4, 4.4)	95.4 (94.4, 96.5)	1.2 (0.8, 1.5)	4.8 (3.0, 6.5)	94.4 (92.6, 96.2)	0.8 (0.1, 1.6)	2.2 (1.3, 3.2)	96.3 (95.2, 97.5)	1.4 (1.0, 1.9)	–	–	–	3.4 (2.4, 4.3)	95.5 (94.4, 96.5)	1.2 (0.8, 1.5)
Wallis and Futuna	9.2 (7.0, 11.5)	74.0 (70.3, 77.8)	16.7 (13.2, 20.3)	13.0 (9.5, 16.4)	72.3 (67.1, 77.6)	14.7 (10.1, 19.3)	5.7 (2.8, 8.5)	76.0 (70.5, 81.5)	18.3 (13.5, 23.2)	10.4 (6.5, 14.4)	70.4 (66.3, 74.6)	19.1 (13.7, 24.6)	8.4 (5.7, 11.1)	76.4 (71.9, 81.0)	15.2 (11.4, 19.0)
**Pooled estimates**	**8.8 (7.2, 10.4)**	**76.2 (71.8, 80.5)**	**14.1 (11.3, 16.8)**	**10.6 (8.6, 12.6)**	**74.6 (69.9, 79.2)**	**13.3 (10.4, 16.3)**	**6.5 (5.0, 7.9)**	**78.1 (73.6, 82.6)**	**14.1 (11.2, 16.9)**	**9.3 (7.2, 11.3)**	**73.1 (67.1, 79.1)**	**16.4 (12.2, 20.6)**	**8.0(6.5, 9.6)**	**78.0 (74.1, 82.0)**	**12.4 (10.1, 14.8)**
**I^2 (%)**	**96.3**	**99.0**	**99.0**	**95.3**	**98.4**	**98.0**	**94.0**	**98.4**	**98.2**	**93.3**	**98.0**	**97.9**	**94.5**	**98.5**	**98.4**
**Total**															
**Pooled estimates**	**8.6 (5.5, 11.7)**	**80.9 (74.7, 87.1)**	**9.7 (5.8, 13.6)**	**10.6 (6.6, 14.5)**	**79.6 (73.1, 86.1)**	**8.9 (5.3, 12.5)**	**6.4 (3.9, 9.0)**	**82.7 (76.9, 88.5)**	**10.1 (6.1, 14.1)**	**8.8 (5.4, 12.3)**	**80.0 (72.8, 87.3)**	**10.2 (6.5, 14.0)**	**8.4 (5.3, 11.6)**	**81.5 (74.1, 88.9)**	**9.2 (5.4, 13.0)**
**I^2 (%)**	**94.6**	**96.1**	**94.1**	**93.8**	**95.3**	**93.5**	**93.6**	**94.0**	**93.0**	**91.9**	**94.2**	**92.7**	**95.0**	**95.6**	**94.7**

Table S6 Prevalence of washing hands across different BMI levels

	Never washing hands before eating				Never washing hands after using the toilet				Never washing hands with soap			
	Underweight	Normal	Overweight	Obese	Underweight	Normal	Overweight	Obese	Underweight	Normal	Overweight	Obese
**Africa Region**												
Algeria	7.5 (1.4, 13.5)	5.5 (4.5, 6.4)	5.7 (2.7, 8.8)	5.3 (1.2, 9.3)	3.0 (0.1, 5.9)	2.8 (1.9, 3.7)	3.6 (1.1, 6.0)	1.9 (0.0, 3.9)	3.8 (0.0, 7.8)	5.2 (4.3, 6.0)	5.3 (1.9, 8.7)	4.2 (1.1, 7.4)
Ghana	8.9 (1.3, 16.6)	8.3 (6.7, 9.9)	8.5 (4.5, 12.4)	9.9 (4.7, 15.0)	12.5 (6.3, 18.7)	11.7 (9.4, 14.0)	11.1 (6.6, 15.6)	10.4 (6.8, 14.0)	16.2 (10.2, 22.3)	12.5 (10.3, 14.7)	16.4 (12.6, 20.2)	15.4 (9.3, 21.6)
Kenya	–	6.4 (2.9, 9.9)	7.7 (6.6, 8.8)	7.9 (5.5, 10.4)	–	15.1 (11.2, 19.0)	22.3 (9.3, 35.3)	13.4 (9.3, 17.5)	18.6 (18.3, 18.9)	20.7 (15.3, 26.1)	31.7 (12.6, 50.8)	–
Namibia	1.3 (1.1, 1.6)	5.3 (3.8, 6.8)	7.0 (2.9, 11.1)	9.8 (1.4, 18.3)	8.8 (2.4, 15.1)	6.5 (4.9, 8.1)	4.4 (2.0, 6.7)	6.8 (2.9, 10.7)	10.2 (4.3, 16.1)	10.5 (7.8, 13.3)	8.8 (5.6, 11.9)	11.0 (4.3, 17.8)
Senegal	36.0 (17.2, 54.8)	17.6 (2.8, 32.4)	7.1 (0.0, 17.1)	6.4 (0.0, 17.4)	34.7 (15.8, 53.5)	14.6 (0.0, 31.5)	5.8 (0.0, 16.0)	6.4 (0.0, 17.4)	34.7 (15.8, 53.5)	19.4 (5.2, 33.7)	10.7 (0.8, 20.6)	9.6 (0.0, 20.9)
Swaziland	7.8 (3.6, 11.9)	3.2 (1.5, 4.8)	4.9 (0.6, 9.2)	3.3 (0.0, 7.0)	1.8 (0.0, 5.7)	2.9 (2.0, 3.8)	4.3 (1.1, 7.6)	–	8.7 (4.0, 13.3)	11.8 (9.5, 14.1)	12.6 (5.9, 19.4)	11.6 (3.2, 20.1)
Uganda	5.4 (2.9, 7.9)	6.3 (4.3, 8.2)	6.8 (2.9, 10.8)	8.6 (2.9, 14.2)	5.1 (2.6, 7.5)	7.8 (5.2, 10.4)	8.9 (4.0, 13.8)	8.1 (1.3, 14.9)	11.9 (3.8, 20.0)	14.3 (11.8, 16.7)	18.9 (10.9, 27.0)	14.1 (6.2, 22.1)
**Pooled estimates**	6.9 (2.8, 11.0)	5.9 (4.5, 7.3)	7.3 (6.4, 8.3)	6.8 (5.0, 8.7)	6.5 (2.8, 10.2)	7.6 (4.8, 10.4)	6.6 (3.9, 9.2)	7.8 (3.4, 12.2)	13.1 (7.0, 19.3)	12.6 (8.5, 16.7)	12.6 (8.1, 17.2)	10.6 (5.9, 15.2)
**I^2 (%)**	87.6	73.2	0.0	19.1	78.0	94.3	66.3	85.9	93.0	95.0	79.1	66.5
**European Region**												
Macedonia	3.2 (0.0, 8.2)	2.0 (1.1, 2.9)	0.8 (0.0, 2.6)	2.7 (0.0, 7.4)	1.8 (0.0, 5.6)	1.7 (0.7, 2.6)	2.4 (0.0, 5.7)	1.2 (0.0, 3.8)	9.7 (3.5, 16.0)	3.4 (1.7, 5.0)	3.9 (0.0, 8.3)	1.7 (0.0, 5.8)
**Pooled estimates**	–	–	–	–	–	–	–	–	–	–	–	–
**I^2 (%)**	–	–	–	–	–	–	–	–	–	–	–	–
**America Region**												
Argentina	10.0 (6.9, 13.0)	11.0 (9.4, 12.5)	9.9 (6.7, 13.1)	9.4 (6.6, 12.3)	8.6 (6.7, 10.5)	5.8 (4.6, 6.9)	5 .0(2.7, 7.3)	3.1 (1.8, 4.5)	10.3 (6.0, 14.6)	5.4 (4.4, 6.3)	5.7 (3.3, 8.1)	6.0(3.4, 8.5)
Barbados	15.9 (7.3, 24.5)	13.9 (11.4, 16.4)	18.1 (9.4, 26.9)	13.9 (4.3, 23.4)	6.0 (0.0, 12.0)	1.6 (0.8, 2.3)	3.1 (0.0, 6.8)	2.6 (2.0, 3.1)	11.1 (3.0, 19.2)	7.6 (5.5, 9.6)	9.6 (3.2, 16.0)	7.8 (0.5, 15.1)
Belize	–	4.0 (3.3, 4.8)	2.5 (0.0, 5.3)	2.0 (1.5, 2.6)	1.6 (0.0, 4.9)	1.8 (1.2, 2.4)	0.8 (0.0, 2.5)	–	3.6 (0.0, 8.6)	4.7 (3.1, 6.4)	4.6 (0.2, 9.1)	2.4 (0.0, 6.7)
Bolivia	12.9 (8.4, 17.3)	10.7 (9.1, 12.2)	8.6 (5.2, 11.9)	14.3 (10.0, 18.6)	6.5 (3.3, 9.7)	7.5 (5.9, 9.1)	4.8 (1.5, 8.0)	5.0(1.5, 8.6)	15.5 (11.7, 19.2)	16.6 (14.4, 18.8)	11.9 (7.8, 16.1)	18.2 (12.1, 24.3)
Cayman	15.4 (0.0,	9.3 (6.6,	20.6 (9.4,	20.8 (5.1,	8.9 (0.0,	4.5 (2.5,	4.6 (0.0,	7.1 (0.0,	21.7 (3.1,	4.6 (2.6,	8.6 (1.0,	9.8 (0.0,

	30.9)	12.1)	31.8)	36.6)	21.9)	6.5)	9.9)	17.2)	40.2)	6.6)	16.2)	21.0)
Bahamas	9.8 (1.5, 18.2)	15.0 (12.1, 17.9)	19.9 (12.3, 27.4)	15.0 (3.9, 26.1)	3.6 (0.0, 8.3)	4.9 (3.4, 6.3)	2.9 (0.0, 5.7)	1.2 (0.0, 3.8)	12.7 (2.2, 23.2)	8.9 (7.2, 10.7)	7.1 (2.4, 11.8)	4.1 (0.0, 10.6)
Costa Rica	7.5 (3.6, 11.4)	10.0 (8.3, 11.8)	6.7 (4.1, 9.3)	11.9 (4.2, 19.7)	1.8 (0.0, 4.4)	2.2 (1.4, 2.9)	2.3 (1.2, 3.4)	0.7 (0.0, 2.2)	2.9 (0.0, 7.2)	6.0(4.5, 7.4)	3.4 (1.1, 5.8)	4.5 (0.0, 9.0)
Curaçao	14.4 (6.9, 22.0)	10.6 (7.7, 13.5)	18.0 (8.7, 27.4)	15.8 (4.7, 26.9)	11.5 (0.0, 24.4)	2.4 (1.2, 3.7)	5.6 (0.1, 11.1)	–	2.2 (0.0, 6.7)	6.6 (4.4, 8.8)	6.7 (0.3, 13.0)	8.5 (0.8, 16.2)
Ecuador	12.6 (9.3, 15.9)	10.4 (8.0, 12.7)	10.9 (7.3, 14.4)	13.9 (9.2, 18.6)	1.2 (0.9, 1.5)	4.9 (3.6, 6.3)	3.3 (0.0, 6.7)	7.0(3.9, 10.0)	10.2 (5.5, 14.9)	7.9 (6.2, 9.5)	7.1 (3.6, 10.7)	9.5 (6.0, 13.1)
El Salvador	3.5 (0.4, 6.7)	5.0 (3.5, 6.4)	5.4 (1.5, 9.4)	8.3 (0.0, 17.4)	5.9 (0.0, 13.6)	3.5 (2.2, 4.9)	1.9 (0.0, 4.0)	4.6 (0.0, 10.2)	6.6 (1.8, 11.5)	5.2 (3.9, 6.6)	2.6 (0.0, 5.4)	10.1 (1.2, 19.1)
Guatemala	2.7 (0.0, 6.3)	4.3 (3.0, 5.6)	5.6 (2.2, 9.0)	3.1 (0.3, 6.0)	4.3 (0.0, 10.7)	2.6 (1.0, 4.1)	0.8 (0.2, 1.4)	2.6 (0.0, 5.9)	5.2 (0.0, 11.2)	5.8 (4.4, 7.3)	5.9 (1.7, 10.1)	1.2 (0.3, 2.1)
Guyana	3.3 (0.0, 6.6)	9.6 (7.2, 11.9)	8.6 (2.8, 14.3)	10.5 (4.0, 17.0)	6.5 (4.1, 9.0)	6.0 (4.3, 7.7)	6.9 (2.5, 11.4)	8.3 (4.0, 12.7)	5.2 (0.9, 9.6)	11.0 (7.9, 14.0)	12.7 (7.0, 18.4)	12.4 (5.9, 18.9)
Honduras	5.3 (0.0, 11.7)	7.7 (6.6, 8.8)	6.4 (3.1, 9.6)	7.4 (2.8, 12.0)	2.9 (0.0, 6.8)	5.5 (3.5, 7.5)	4.6 (1.9, 7.2)	2.7 (0.0, 7.1)	64.7 (54.2, 75.3)	58.7 (54.2, 63.2)	52.7 (41.4, 64.0)	62.5 (48.1, 76.9)
Peru	6.8 (2.7, 10.9)	4.8 (3.4, 6.3)	6.0 (2.6, 9.4)	3.1 (0.0, 6.3)	12.3 (7.9, 16.6)	6.3 (5.0, 7.6)	7.9 (2.8, 12.9)	3.5 (0.0, 7.3)	6.6 (3.0, 10.3)	7.7 (5.4, 9.9)	9.8 (5.0, 14.5)	6.4 (2.8, 10.0)
Saint Kitts and Nevis	11.5 (3.0, 20.0)	12.3 (10.2, 14.5)	12.6 (6.2, 19.0)	12.0 (3.8, 20.2)	2.1 (0.0, 6.3)	3.0 (1.8, 4.2)	2.1 (0.0, 4.6)	6.4 (0.1, 12.6)	7.0 (0.0, 15.0)	7.4 (5.7, 9.1)	6.8 (1.8, 11.8)	3.6 (0.0, 8.7)
Trinidad and Tobago	11.4 (4.0, 18.7)	11.2 (9.5, 12.8)	8.9 (4.7, 13.0)	12.4 (5.3, 19.5)	2.5 (1.1, 3.8)	2.6 (1.6, 3.7)	3.3 (0.3, 6.3)	–	10.1 (4.6, 15.6)	9.7 (8.2, 11.2)	8.2 (5.0, 11.5)	10.8 (2.8, 18.8)
Uruguay	7.8 (3.2, 12.4)	12.6 (10.4, 14.8)	16.2 (10.5, 21.8)	10.9 (4.9, 17.0)	4.4 (0.6, 8.2)	7.4 (5.6, 9.1)	5.2 (2.6, 7.7)	4.4 (0.4, 8.4)	2.1 (0.0, 4.6)	3.5 (2.3, 4.7)	5.1 (1.7, 8.4)	1.3 (0.0, 3.2)
Venezuela	1.1 (0.0, 3.6)	6.0 (3.6, 8.4)	2.9 (0.0, 5.9)	6.3 (0.1, 12.6)	–	2.8 (1.9, 3.6)	1.0 (0.0, 2.7)	2.8 (0.0, 7.5)	7.4 (0, 14.9)	3.6 (2.4, 4.9)	4.3 (0.0, 8.9)	3.0 (0.0, 7.2)
**Pooled estimates**	**8.0 (5.6, 10.5)**	**9.3 (7.6, 10.9)**	**9.0 (7.0, 11.0)**	**9.6 (6.8, 12.3)**	**4.6 (3.0, 6.2)**	**4.1 (3.2, 4.9)**	**3.0 (2.1, 3.9)**	**3.2 (2.2, 4.2)**	**10.1 (6.4, 13.7)**	**9.7 (7.2, 12.1)**	**8.1 (5.8, 10.4)**	**7.6 (5.1, 10.1)**
**I^2 (%)**	**81.4**	**94.4**	**76.1**	**87.2**	**86.7**	**90.0**	**68.3**	**59.4**	**90.4**	**97.6**	**82.5**	**88.7**
**Eastern Mediterranean Region**												
Afghanistan	12.8 (4.5, 21.1)	4.3 (2.4, 6.1)	10.0 (5.7, 14.4)	3.4 (0.0, 8.3)	1.1 (0.0, 3.8)	5.3 (2.6, 7.9)	6.4 (1.9, 11.0)	2.5 (0.0, 5.2)	11.8 (5.4, 18.1)	12.4 (7.0, 17.7)	10.5 (3.4, 17.6)	6.6 (0.0, 19.4)
Djibouti	8.3 (0.0, 20.9)	5.8 (4.2, 7.4)	4.4 (0.0, 9.1)	4.1 (0.0, 12.7)	17.7 (1.4, 34.0)	13.1 (10.7, 15.6)	12.0(7.3, 16.7)	10.4 (0.0, 21.7)	5.3 (0.0, 12.5)	12.1 (8.7, 15.4)	13.2 (5.0, 21.4)	8.3 (0.0, 19.4)
Egypt	11.4 (3.7, 19.0)	12.8 (9.6, 16.0)	11.1 (5.8, 16.3)	13.8 (4.4, 23.2)	11.0 (5.0, 17.1)	10.1 (6.5, 13.7)	9.7 (4.2, 15.2)	–	1.7 (0.0, 3.7)	8.1 (4.9, 11.3)	9.3 (4.5, 14.1)	4.3 (0.0, 9.0)
Iraq	13.2 (4.1,	6.7 (4.7,	11.2 (7.8,	4.4 (0.0,	8.8 (0.9,	8.0 (5.8,	6.9 (2.4,	8.3 (0.5,	3.3 (0.0, 8.2)	3.9 (2.8,	4.8 (0.6,	5.9 (0.0,

	22.4)	8.7)	14.6)	11.3)	16.8)	10.2)	11.4)	16.2)		5.1)	9.0)	12.9)
Jordan	2.4 (0.0, 8.4)	6.1 (4.1, 8.0)	11.6 (4.6, 18.6)	3.4 (0, 8.4)	5.0 (0.1, 9.8)	5.0 (2.2, 7.8)	16.2 (8.3, 24.1)	9.7 (0.0, 21.4)	5.0(0.0, 12.1)	9.6 (6.8, 12.3)	11.9 (5.2, 18.5)	7.6 (0.0, 15.6)
Kuwait	14 (7.9, 20.0)	9.7 (7.5, 11.9)	8.7 (4.0, 13.3)	7.2 (1.3, 13.1)	8.2 (0.4, 16.0)	5.4 (3.7, 7.1)	5.9 (3.6, 8.2)	6.8 (1.8, 11.9)	7.6 (1.9, 13.3)	6.3 (4.1, 8.6)	7.4 (4.8, 10.1)	6.4 (2.4, 10.5)
Lebanon	6.8 (0.0, 18.2)	4.9 (3.8, 6.1)	7.4 (2.9, 11.9)	4.0 (0.0, 11.3)	1.4 (1.0, 1.8)	2.4 (0.3, 4.4)	3.5 (0.0, 7.5)	1.1 (0.0, 3.3)	–	1.4 (0.5, 2.4)	3.4 (0.0, 6.8)	5.0 (0.0, 11.9)
Libya	10.0 (7.3, 12.7)	7.6 (5.1, 10.1)	7.7 (2.6, 12.8)	5.2 (0.2, 10.2)	8.0 (1.0, 14.9)	6.2 (4.4, 8.1)	8.6 (4.7, 12.4)	7.7 (2.9, 12.5)	7.4 (1.5, 13.4)	7.0 (5.4, 8.6)	8.7 (4.2, 13.2)	5.2 (0.0, 11.1)
Morocco	4.0 (0.0, 8.1)	3.5 (2.4, 4.6)	1.9 (0.0, 4.5)	6.0 (0.0, 12.7)	8.9 (4.0, 13.7)	5.0 (3.8, 6.2)	3.6 (1.3, 6.0)	9.8 (4.4, 15.2)	11.4 (6.2, 16.5)	7.4 (5.7, 9.0)	5.7 (2.1, 9.3)	6.6 (1.9, 11.3)
UNRWA	5.4 (2.8, 8.0)	6.5 (5.1, 7.9)	6.7 (4.4, 8.9)	4.9 (2.0, 7.8)	2.8 (1.0, 4.7)	4.9 (4.0, 5.9)	4.2 (2.2, 6.1)	2.8 (0.6, 4.9)	6.0 (3.2, 8.9)	6.0 (4.4, 7.6)	3.3 (1.5, 5.2)	1.5 (0.3, 2.8)
Oman	7.4 (0.0, 15.2)	8.1 (5.8, 10.4)	5.7 (1.5, 10.0)	6.6 (0.9, 12.2)	8.7 (7.4, 9.9)	9.4 (6.8, 11.9)	6.6 (2.5, 10.8)	3.0 (0.8, 5.3)	9.2 (6.7, 11.8)	7.1 (4.8, 9.5)	6.0(1.2, 10.8)	6.2 (0.5, 11.8)
Pakistan	2.8 (0.8, 4.9)	3.6 (2.6, 4.5)	3.8 (1.8, 5.9)	2.4 (0.7, 4.0)	3.9 (1.5, 6.2)	3.5 (2.3, 4.8)	3.1 (0.9, 5.3)	2.0 (0.2, 3.9)	8.3 (4.3, 12.4)	8.0 (6.0, 10.0)	10.3 (6.0, 14.5)	4.5 (1.6, 7.5)
Qatar	12.4 (0.0, 26.8)	17.4 (10.2, 24.5)	10.8 (6.3, 15.4)	24.4 (13.8, 35.0)	15.2 (5.3, 25.1)	14.9 (9.2, 20.6)	8.2 (5.4, 11.0)	7.0 (0.0, 14.8)	9.3 (0.0, 24.1)	16.8 (10.5, 23.1)	11.1 (4.6, 17.7)	7.6 (0.0, 16.1)
Sudan	9.4 (0.0, 18.8)	7.2 (4.9, 9.4)	5.0 (2.3, 7.7)	3.7 (0.0, 9.5)	11.4 (0.0, 26.7)	11.2 (8.6, 13.7)	9.9 (4.0, 15.8)	9.7 (3.4, 16.0)	27.6 (13.7, 41.5)	21.4 (16.7, 26.1)	12.4 (5.4, 19.5)	11.5 (4.4, 18.5)
Syrian Arab Republic	9.5 (1.5, 17.5)	8.0 (6.3, 9.8)	16.0 (9.6, 22.3)	14.7 (9.6, 19.8)	5.4 (0.0, 10.8)	3.4 (1.8, 5.0)	7.0 (3.2, 10.8)	2.1 (0.0, 4.6)	4.1 (0.0, 10.7)	6.4 (4.7, 8.0)	7.7 (2.0, 13.3)	7.6 (1.5, 13.6)
Tunisia	2.0 (0.0, 6.7)	5.6 (3.8, 7.5)	9.5 (0.9, 18.0)	7.9 (2.3, 13.5)	8.1 (4.5, 11.6)	4.1 (2.8, 5.4)	4.7 (0.0, 9.5)	7.0(0.7, 13.3)	3.6 (0.0, 7.6)	5.5 (3.4, 7.6)	4.9 (0.3, 9.5)	6.1 (0.4, 11.8)
United Arab Emirates	7.1 (3.4, 10.8)	8.4 (6.5, 10.4)	8.7 (5.6, 11.8)	4.0 (0.3, 7.7)	2.5 (0.2, 4.8)	3.0 (1.8, 4.1)	4.2 (1.7, 6.6)	2.0 (0.0, 4.1)	4.1 (0.8, 7.4)	4.0 (2.5, 5.5)	4.3 (1.8, 6.9)	5.7 (1.9, 9.4)
Yemen	–	7.8 (5.2, 10.4)	16.0 (0.0, 33.0)	11.0 (0.9, 21.1)	4.3 (0.0, 14.8)	14.1 (10.2, 18.0)	20.4 (5.0, 35.7)	9.2 (0.0, 19.8)	16.3 (1.0, 31.5)	21.8 (16.0, 27.5)	9.9 (4.1, 15.6)	17.7 (8.3, 27.1)
**Pooled estimates**	**7.0 (5.0, 9.0)**	**6.8 (5.8, 7.9)**	**7.8 (6.1, 9.5)**	**6.1 (4.2, 7.9)**	**5.9 (3.9, 7.9)**	**6.7 (5.4, 7.9)**	**6.4 (5.1, 7.7)**	**3.9 (2.6, 5.1)**	**6.7 (4.7, 8.6)**	**8.3 (6.6, 10.1)**	**7.1 (5.6, 8.5)**	**5.9 (4.2, 7.7)**
**I^2 (%)**	**63.1**	**84.1**	**70.0**	**61.6**	**90.0**	**88.9**	**58.5**	**51.8**	**68.6**	**92.3**	**52.8**	**53.7**
**Southeast Asia Region**												
Bangladesh	1.0 (0.0, 2.5)	3.3 (1.2, 5.4)	2.4 (0.2, 4.7)	2.7 (0.0, 6.3)	0.2 (0.0, 0.7)	2.3 (0.1, 4.5)	2.3 (0.3, 4.3)	–	4.2 (0.0, 12.2)	5.4 (2.2, 8.7)	5.3 (0.0, 11.4)	2.3 (0.0, 5.8)
India	9.6 (5.6, 13.7)	5.6 (4.6, 6.6)	7.1 (4.1, 10.0)	6.6 (3.1, 10.1)	4.3 (0.6, 8.0)	3.0(2.2, 3.8)	5.1 (3.2, 7.0)	2.4 (0.6, 4.3)	14.3 (7.6, 20.9)	13.3 (11.4, 15.3)	11.0 (6.9, 15.0)	12.3 (8.7, 15.9)
Indonesia	2.0 (0.5, 3.5)	2.4 (1.9, 2.8)	2.0 (0.9, 3.2)	2.7 (0.9, 4.4)	1.8 (0.2, 3.3)	2.2 (1.6, 2.7)	1.6 (0.6, 2.7)	1.7 (0.4, 3.1)	4.0 (1.7, 6.3)	3.8 (3.0, 4.5)	4.1 (2.4, 5.9)	1.6 (0.3, 3.0)

Thailand	14.9 (11.1, 18.8)	14.4 (11.5, 17.3)	12.7 (8.7, 16.7)	25.7 (16.1, 35.2)	6.3 (2.1, 10.5)	6.4 (5.1, 7.7)	4.7 (1.5, 8.0)	9.2 (5.1, 13.2)	13.8 (7.2, 20.4)	13.8 (11.5, 16.1)	14.3 (9.9, 18.8)	15.6 (7.5, 23.6)
Timor-Leste	18.5 (12.2, 24.8)	20.1 (17.2, 23.0)	22.4 (17.6, 27.3)	15.2 (4.5, 26.0)	31.9 (21.3, 42.6)	25.7 (22.3, 29.2)	28.3 (21.4, 35.2)	27.7 (12.7, 42.7)	17.9 (10.4, 25.4)	17.2 (14.5, 19.9)	18.1 (10.6, 25.7)	13.0 (5.6, 20.5)
**Pooled estimates**	**8.5 (3.6, 13.4)**	**8.9 (4.6, 13.3)**	**8.9 (3.5, 14.4)**	**8.1 (3.3, 13.0)**	**5.0 (1.6, 8.4)**	**7.4 (3.9, 10.9)**	**6.7 (2.8, 10.6)**	**5.4 (1.5, 9.2)**	**10.4 (4.5, 16.3)**	**10.7 (4.7, 16.6)**	**10.1 (4.9, 15.4)**	**8.1 (2.8, 13.4)**
**I^2 (%)**	**95.1**	**98.2**	**95.6**	**86.2**	**92.0**	**98.0**	**93.8**	**86.8**	**83.3**	**98.0**	**87.9**	**91.3**
**Western Pacific Region**												
Brunei Darussalam	1.5 (0.0, 4.4)	3.6 (2.6, 4.6)	5.2 (1.7, 8.6)	2.1 (0.0, 6.3)	1.0(0.0, 3.1)	3.0(1.7, 4.4)	2.4 (0.0, 4.8)	0.8 (0.0, 2.6)	8.2 (1.9, 14.5)	9.7 (7.9, 11.6)	14.2 (8.4, 20.0)	2.5 (0.0, 6.0)
Cambodia	–	2.0 (1.2, 2.8)	3.2 (1.1, 5.3)	–	4.4 (0.3, 8.4)	3.0 (2.1, 3.9)	4.1 (0.5, 7.7)	2.2 (0.0, 6.9)	0.5 (0.0, 1.7)	2.8 (2.1, 3.6)	0.9 (0.0, 2.3)	–
China	5.4 (2.9, 7.9)	4.4 (3.6, 5.2)	4.6 (2.6, 6.5)	3.3 (1.3, 5.3)	2.5 (0.3, 4.7)	2.6 (2.1, 3.2)	3.3 (1.3, 5.4)	4.5 (2.0, 7.0)	10.7 (6.3, 15.1)	10.9 (9.6, 12.1)	7.5 (5.5, 9.5)	11.6 (8.1, 15.1)
Cook	21.0 (14.1, 27.9)	4.9 (1.6, 8.1)	24.4 (10.5, 38.3)	–	15.0 (0.0, 38.7)	3.3 (0.3, 6.2)	3.2 (0.0, 12.2)	9.4 (0.0, 23.2)	14.8 (0.0, 45.3)	6.7 (3.2, 10.2)	15.6 (1.1, 30.2)	14.8 (0.0, 31.9)
Kiribati	11.8 (5.7, 17.8)	21.4 (15.5, 27.4)	18.1 (10.7, 25.5)	27.0 (17.1, 36.9)	22.4 (12.5, 32.2)	15.7 (12.4, 19.0)	16.2 (8.7, 23.7)	23.7 (12.9, 34.4)	13.2 (2.1, 24.2)	15.1 (12.4, 17.8)	18.4 (11.4, 25.4)	20.6 (11.5, 29.8)
Laos	1.2 (0.0, 4.0)	2.0 (0.8, 3.3)	1.9 (0.0, 4.1)	–	5.1 (0.6, 9.6)	3.7 (1.9, 5.6)	5.4 (0.0, 10.8)	1.2 (0.0, 3.7)	8.9 (0.5, 17.3)	8.5 (5.7, 11.2)	9.0 (5.2, 12.7)	6.8 (2.2, 11.3)
Malaysia	7.0 (3.8, 10.3)	4.8 (4.2, 5.5)	5.6 (4.3, 6.9)	4.0 (2.4, 5.6)	9.6 (5.8, 13.4)	6.0 (5.3, 6.6)	3.8 (2.6, 4.9)	5.5 (3.8, 7.3)	15.0 (10.9, 19.1)	13.6 (12.5, 14.7)	12.7 (10.6, 14.9)	12.6 (9.7, 15.4)
Mongolia	3.8 (0.2, 7.4)	7 (6, 8)	5.6 (2.8, 8.5)	10.1 (5.3, 14.9)	13.0 (7.0, 19.0)	10.5 (8.8, 12.3)	8.8 (4.6, 12.9)	8.8 (4.5, 13.1)	3.6 (0.1, 7.0)	2.9 (2.1, 3.8)	3.9 (1.5, 6.4)	2.1 (0.0, 4.2)
Philippines	2.0 (0.2, 3.7)	4.1 (3.2, 5.0)	6.7 (4.4, 9.1)	6.0 (3.6, 8.5)	1.5 (0.1, 2.9)	3.6 (2.8, 4.3)	3.4 (2.0, 4.8)	3.1 (1.0, 5.2)	5.1 (2.5, 7.8)	5.1 (4.4, 5.8)	4.5 (2.6, 6.5)	4.1 (1.7, 6.6)
Samoa	8.3 (0.0, 17.4)	12.7 (10.1, 15.3)	16.2 (9.4, 22.9)	10.1 (4.5, 15.6)	15.0 (1.7, 28.3)	15.8 (13.3, 18.3)	15.6 (9.2, 21.9)	8.6 (0.1, 17.1)	15.8 (6.8, 24.9)	17.6 (14.2, 21.0)	16.3 (9.0, 23.5)	12.7 (3.6, 21.7)
Solomon	7.3 (0.0, 17.0)	8.5 (6.5, 10.6)	10.8 (4.6, 16.9)	5.9 (0.0, 11.9)	8.4 (0.0, 18.6)	9.4 (6.8, 12.0)	6.1 (2.6, 9.7)	4.1 (0.4, 7.9)	12.8 (5.6, 19.9)	10 (5.3, 14.8)	13.5 (5.8, 21.1)	5.2 (0.0, 13.8)
Tonga	8 (1.5, 14.0)	11.7 (9.7, 13.7)	10.0 (5.3, 14.7)	7.8 (2.7, 12.8)	2.0 (0.0, 4.8)	7.3 (5.7, 8.8)	4.8 (2.2, 7.3)	7.4 (1.0, 13.8)	19.3 (13.2, 25.3)	18.2 (15.9, 20.5)	15.5 (10.5, 20.5)	9.8 (4.0, 15.7)
Tuvalu	54.2 (29.0, 79.3)	37.1 (32.1, 42.1)	35.0 (20.0, 49.9)	43.2 (23.2, 63.2)	36.7 (11.9, 61.5)	17.1 (13.1, 21.2)	15.2 (4.3, 26.1)	10.9 (0.0, 23.4)	4.7 (0.0, 14.5)	16.2 (12.4, 20.0)	21.2 (8.1, 34.4)	21.3 (5.0, 37.6)
Vanuatu	10.8 (0.0, 29.7)	5.1 (3.3, 6.9)	10.9 (0.0, 23.4)	1.5 (1.2, 1.7)	7.1 (0.0, 27.5)	7.9 (4.1, 11.6)	6.9 (0.0, 15.5)	3.6 (3.0, 4.2)	21.5 (0.0, 43.1)	17.4 (14.2, 20.6)	11.8 (2.3, 21.2)	22.1 (0.4, 43.8)
Vietnam	1.2 (0.8, 1.6)	7.2 (5.1, 9.3)	6.1 (1.6, 10.6)	12.6 (2.9, 22.3)	1.3 (0.0, 4.3)	2.2 (1.0, 3.4)	3.4 (0.0, 7.2)	1.0 (0.7, 1.2)	7.7 (1.1, 14.3)	9.0 (6.2, 11.8)	4.7 (3.6, 5.8)	8.0 (3.0, 13.0)
Wallis and	–	13.2 (10.1,	12.2 (3.6,	6.9 (0.0,	9.6 (0.0,	8.6 (6.2,	6.6 (1.4,	–	6.6 (0.0,	8.6 (6.3,	7.3 (1.3,	2.4 (0.0,

Futuna		16.3)	20.7)	15.8)	20.7)	11.0)	11.7)		20.4)	10.9)	13.4)	7.6)
**Pooled estimates**	**5.6 (3.5, 7.7)**	**8.3 (6.6, 10.0)**	**7.7 (5.7, 9.6)**	**6.8 (4.5, 9.1)**	**5.4 (3.3, 7.4)**	**7.1 (5.5, 8.6)**	**5.2 (3.9, 6.5)**	**3.8 (2.5, 5.2)**	**9.5 (5.9, 13.0)**	**10.7 (8.2, 13.2)**	**9.6 (7.1, 12.2)**	**8.2 (5.4, 10.9)**
**I^2 (%)**	**86.0**	**96.3**	**78.8**	**88.1**	**77.3**	**95.7**	**61.1**	**89.3**	**89.0**	**97.7**	**91.4**	**81.5**
**Total**												
**Pooled estimates**	**6.6 (5.4, 7.8)**	**6.7 (4.1, 9.3)**	**6.7 (3.9, 9.6)**	**6.7 (5.3, 8.2)**	**4.9 (3.7, 6.0)**	**5.6 (3.5, 7.6)**	**4.9 (3.3, 6.5)**	**3.5 (2.5, 4.6)**	**9.0 (7.1, 10.9)**	**8.9 (6.0, 11.9)**	**8.3 (6.5, 10.1)**	**6.7 (4.5, 8.9)**
**I^2 (%)**	**9.4**	**94.9**	**94.2**	**47.4**	**26.0**	**92.2**	**80.5**	**55.0**	**28.3**	**87.8**	**55.5**	**69.6**

Table S7 Prevalence of daily tooth brushing across different BMI levels

	Underweight			Normal			Overweight			Obese		
	0	1–3 times	>3 times	0	1–3 times	>3 times	0	1–3 times	>3 times	0	1–3 times	>3 times
**Africa Region**												
Algeria	24.5 (18.0, 31.0)	63.1 (54.1, 72.2)	12.3 (7.6, 17.1)	19.9 (16.8, 23.0)	67.5 (64.1, 70.8)	12.6 (10.3, 14.9)	15.4 (10.5, 20.4)	67.2 (61.4, 73.0)	17.4 (13.7, 21.1)	16.2 (10.8, 21.5)	68.9 (64.2, 73.7)	14.9 (10.6, 19.2)
Ghana	16.1 (10.3, 21.8)	54.9 (46.8, 63.1)	29.0 (20.8, 37.2)	13.1 (11.0, 15.1)	62.6 (59.3, 65.8)	24.4 (22.2, 26.6)	13.9 (9.6, 18.2)	65.9 (58.6, 73.2)	20.2 (14.2, 26.2)	10.9 (6.7, 15.0)	70.9 (63.6, 78.2)	18.2 (12.9, 23.5)
Namibia	17.9 (8.7, 27.1)	63.8 (54.9, 72.7)	18.3 (13.0, 23.6)	12.5 (9.1, 15.8)	67.6 (62.6, 72.5)	20.0 (16.2, 23.8)	6.6 (2.4, 10.8)	70.4 (62.2, 78.6)	23.0 (16.0, 30.0)	14.8 (0.0, 29.6)	72.3 (63.1, 81.4)	13.0 (5.9, 20.0)
Senegal	21.9 (10.0, 33.8)	67.2 (49.5, 85.0)	10.9 (5.0, 16.7)	9.6 (3.2, 15.9)	84.4 (79.5, 89.2)	6.1 (3.8, 8.4)	5.9 (0.0, 15.9)	83.6 (77.6, 89.6)	10.5 (3.1, 18.0)	10.2 (3.5, 16.8)	84.1 (74.0, 94.3)	5.7 (0.0, 14.2)
Swaziland	3.6 (0.0, 8.5)	88.3 (79.2, 97.3)	8.2 (2.1, 14.2)	5.1 (3.2, 7.0)	89.7 (87.9, 91.5)	5.2 (3.9, 6.6)	4.2 (2.1, 6.4)	93.5 (88.9, 98.2)	2.2 (0.0, 5.5)	6.1 (0.0, 13.9)	90.4 (83.6, 97.1)	3.6 (0.0, 7.6)
Uganda	1.1 (0.8, 1.4)	76.1 (68.2, 84.0)	22.8 (14.9, 30.8)	1.6 (1.0, 2.3)	83.5 (81.1, 85.9)	14.9 (12.4, 17.4)	2.0 (0.0, 4.2)	84.7 (79.5, 89.8)	13.4 (7.1, 19.6)	0.8 (0.5, 1.0)	83.2 (74.0, 92.5)	16.0 (6.6, 25.3)
**Pooled estimates**	**13.5 (5.1, 22.0)**	**69.0 (58.8, 79.1)**	**16.4 (10.9, 22.0)**	**10.2 (4.3, 16.2)**	**76.0 (66.3, 85.6)**	**13.8 (7.3, 20.3)**	**7.8 (3.6, 12.0)**	**77.8 (68.4, 87.2)**	**14.3 (6.7, 21.9)**	**9.3 (2.6, 16.0)**	**78.1 (70.2, 86.0)**	**11.7 (6.2, 17.2)**
**I^2 (%)**	**95.1**	**85.7**	**79.5**	**98.1**	**98.4**	**98.1**	**88.2**	**93.3**	**93.0**	**92.6**	**85.0**	**82.3**
**European Region**												
Macedonia	7.6 (2.2, 13.0)	88.6 (80.2, 97.0)	3.8 (0.0, 9.7)	2.8 (1.1, 4.4)	91.7 (90.2, 93.3)	5.5 (3.8, 7.2)	2.5 (0.5, 4.4)	93.5 (90.4, 96.6)	4.0 (0.7, 7.4)	1.5 (0.0, 4.7)	90.1 (82.1, 98.0)	8.4 (0.9, 15.9)
**Pooled estimates**	–	–	–	–	–	–	–	–	–	–	–	–
**I^2 (%)**	–	–	–	–	–	–	–	–	–	–	–	–
**America Region**												
Argentina	8.2 (3.6, 12.9)	79.2 (72.5, 85.9)	12.6 (9.4, 15.8)	6.7 (5.7, 7.6)	81.6 (79.8, 83.4)	11.8 (10.1, 13.5)	9.7 (7.0, 12.5)	77.9 (74.0, 81.9)	12.3 (8.9, 15.7)	6.9 (4.5, 9.3)	81.9 (76.9, 86.8)	11.3 (6.3, 16.2)
Bahamas	8.2 (0.6, 15.7)	76.6 (63.7, 89.4)	15.3 (3.9, 26.7)	5.4 (3.4, 7.3)	84.5 (80.2, 88.8)	10.1 (7.1, 13.1)	8.2 (3.1, 13.3)	86.6 (78.8, 94.5)	5.1 (0.7, 9.5)	3.7 (0.0, 9.6)	84.4 (76.2, 92.6)	11.9 (3.8, 20.0)
Barbados	7.9 (0.2, 15.6)	80.3 (68.5, 92.2)	11.8 (4.2, 19.3)	3.0 (1.8, 4.2)	90.5 (88.5, 92.6)	6.5 (4.6, 8.3)	4.4 (0.8, 8.0)	86.6 (77.8, 95.4)	9.0 (0.7, 17.4)	1.3 (0.0, 4.1)	95.2 (91.0, 99.5)	3.4 (0.3, 6.6)
Belize	–	87.1 (77.4, 96.8)	12.9 (3.2, 22.6)	2.1 (1.0, 3.3)	84.7 (81.4, 88.0)	13.1 (10.4, 15.9)	1.1 (0.0, 2.8)	81.5 (71.9, 91.1)	17.3 (8.1, 26.6)	3.0 (0.0, 6.6)	90.9 (83.7, 98.0)	6.2 (0.1, 12.3)
Bolivia	9.4 (3.6, 15.2)	83.1 (75.4, 90.8)	7.5 (1.9, 13.1)	8.4 (7.0, 9.9)	84.8 (83.2, 86.3)	6.8 (5.6, 8.0)	5.4 (2.8, 8.1)	85.6 (82.1, 89.1)	9.0 (5.5, 12.4)	5.4 (2.2, 8.6)	88.7 (83.9, 93.6)	5.9 (2.0, 9.8)
Cayman	15.2 (0.0, 32.3)	76.0 (56.0, 96.0)	8.9 (0.0, 21.9)	3.3 (1.6, 4.9)	92.2 (89.8, 94.7)	4.5 (2.6, 6.4)	5.6 (0.0, 12)	92.8 (85.7, 99.9)	1.6 (0.0, 4.9)	15.7 (0.8, 30.6)	81.2 (65.6, 96.9)	3.1 (0.0, 9.3)

Costa Rica	–	77.4 (70.8, 84.1)	22.6 (15.9, 29.2)	1.8 (1.2, 2.3)	82.1 (79.7, 84.5)	16.1 (14.0, 18.2)	2.6 (0.8, 4.4)	80.5 (74.7, 86.3)	16.9 (12.3, 21.6)	1.4 (0.0, 3.6)	81.3 (76.1, 86.4)	17.3 (13.3, 21.4)
Curaçao	–	91.8 (86.8, 96.8)	8.2 (3.2, 13.2)	3.2 (1.9, 4.6)	94.1 (92.2, 96.0)	2.7 (1.3, 4.0)	5.3 (0.0, 10.6)	85.9 (77.3, 94.4)	8.8 (3.1, 14.5)	7.9 (0.8, 15.0)	85.9 (74.1, 97.7)	6.2 (0.0, 16.0)
Ecuador	4.7 (1.8, 7.6)	95.3 (92.4, 98.2)	–	11.1 (8.9, 13.3)	88.9 (86.7, 91.1)	–	11.6 (7.3, 15.9)	87.7 (83.4, 92.0)	0.7 (0.6, 0.8)	8.9 (4.2, 13.6)	91.1 (86.4, 95.8)	–
El Salvador	–	90.3 (85.5, 95.1)	9.7 (4.9, 14.5)	2.5 (1.5, 3.4)	85.8 (83.8, 87.8)	11.8 (9.7, 13.8)	1.5 (0.0, 4.0)	89.2 (84.0, 94.4)	9.3 (4.0, 14.6)	8.3 (1.3, 15.3)	77.1 (65.2, 89.1)	14.6 (5.4, 23.7)
Guatemala	4.5 (0.0, 10.9)	84.2 (68.8, 99.7)	11.2 (0.7, 21.8)	5.4 (3.4, 7.4)	75.8 (72.1, 79.4)	18.8 (15.1, 22.5)	12.6 (4.7, 20.6)	74.0 (67.1, 80.8)	13.4 (8.5, 18.3)	0.8 (0.2, 1.3)	76.3 (67.0, 85.7)	22.9 (13.2, 32.6)
Guyana	2.2 (1.4, 3.0)	95.2 (93.4, 97.0)	2.6 (1.6, 3.6)	4.7 (3.2, 6.1)	87.8 (84.8, 90.7)	7.5 (5.3, 9.8)	1.8 (0.0, 4.1)	90.7 (86.6, 94.8)	7.5 (5.1, 9.9)	3.6 (0.0, 7.3)	85.6 (81.6, 89.5)	10.8 (4.3, 17.3)
Honduras	2.6 (1.7, 3.4)	82.2 (72.5, 91.9)	15.3 (5.8, 24.8)	4.1 (2.8, 5.3)	81.1 (78.4, 83.8)	14.8 (12.4, 17.3)	5.9 (1.8, 10.1)	75.2 (69.0, 81.5)	18.8 (12.1, 25.5)	1.2 (0.0, 4.0)	83.8 (73.7, 93.8)	15.0 (6.6, 23.4)
Peru	4.5 (1.1, 7.9)	80.5 (75.4, 85.7)	15.0 (11.2, 18.7)	3.8 (2.8, 4.9)	86.9 (84.9, 88.9)	9.3 (7.4, 11.1)	6.2 (2.9, 9.5)	87.3 (83.4, 91.1)	6.5 (3.7, 9.4)	3.3 (0.0, 6.9)	90.4 (85.6, 95.2)	6.3 (3.0, 9.6)
Saint Kitts and Nevis	1.6 (0.0, 4.7)	94.1 (88.1, 100.0)	4.4 (0, 9.4)	4.7 (3.2, 6.1)	86.0 (83.8, 88.3)	9.3 (7.4, 11.2)	1.3 (0.0, 3.2)	88.6 (82.7, 94.6)	10.0 (4.3, 15.7)	1.2 (0.0, 3.5)	87.7 (79.7, 95.7)	11.2 (3.4, 18.9)
Trinidad and Tobago	2.7 (0.0, 6.7)	94.0 (91.0, 97.0)	3.3 (0.0, 7.0)	3.6 (2.1, 5.1)	92.6 (90.8, 94.3)	3.8 (2.6, 5)	6.0 (2.9, 9.0)	90.9 (86.8, 95.1)	3.1 (0.1, 6.0)	1.0 (0.0, 3.2)	96.7 (93.1, 100.0)	2.3 (0.0, 5.1)
Uruguay	3.3 (0.0, 6.9)	75.1 (64.2, 86.0)	21.6 (12.2, 31.0)	2.4 (1.6, 3.2)	77.8 (75.9, 79.7)	19.8 (18.0, 21.6)	3.1 (0.8, 5.4)	75.2 (69.1, 81.2)	21.7 (16.0, 27.4)	3.0 (0.0, 6.4)	74.6 (66.4, 82.9)	22.4 (14.1, 30.7)
Venezuela	2.4 (2.0, 2.8)	67.9 (58.5, 77.4)	29.7 (20.3, 39.0)	1.9 (1.1, 2.7)	83.3 (79.8, 86.8)	14.8 (11.4, 18.2)	1.3 (0.0, 3.5)	85.8 (81.0, 90.6)	12.9 (7.9, 17.8)	0.9 (0.6, 1.2)	82.4 (69.8, 95.1)	16.7 (3.8, 29.6)
**Pooled estimates**	**3.0 (2.3, 3.7)**	**85.3 (81.6, 88.9)**	**11.9 (8.3, 15.5)**	**4.2 (3.3, 5.2)**	**85.7 (83.3, 88.0)**	**10.6 (8.1, 13.1)**	**4.5 (3.1, 5.9)**	**84.7 (82.1, 87.3)**	**9.8 (6.8, 12.9)**	**2.5 (1.6, 3.3)**	**86.2 (83.1, 89.3)**	**10.1 (7.1, 13.1)**
**I^2 (%)**	**44.3**	**86.7**	**89.9**	**92.1**	**94.8**	**96.6**	**79.0**	**76.3**	**95.0**	**72.3**	**77.2**	**81.1**
**Eastern Mediterranean Region**												
Afghanistan	16.1 (8.3, 23.9)	59.4 (44.7, 74.1)	24.5 (7.6, 41.4)	24.3 (18.7, 30.0)	62.7 (59.3, 66.2)	13.0 (8.8, 17.1)	21.7 (10.5, 33.0)	67.1 (57.1, 77.1)	11.2 (6.1, 16.2)	22.4 (2.6, 42.3)	61.0 (44.3, 77.6)	16.6 (7.0, 26.2)
Djibouti	13.2 (4.7, 21.6)	60.4 (47.7, 73.1)	26.5 (11.1, 41.8)	10.9 (8.1, 13.7)	57.0 (53.8, 60.2)	32.1 (29.0, 35.2)	9.6 (2.1, 17.2)	52.8 (40.9, 64.7)	37.6 (25.5, 49.6)	4.4 (0.7, 8.1)	62.0 (46.6, 77.5)	33.6 (17.4, 49.7)
Egypt	42.1 (34.4, 49.7)	46.2 (37.0, 55.4)	11.7 (6.9, 16.5)	39.8 (34.0, 45.7)	51.9 (45.7, 58.2)	8.2 (5.2, 11.2)	34.0 (27.4, 40.5)	56.6 (49.5, 63.8)	9.4 (5.1, 13.8)	35.8 (25.6, 46.1)	55.6 (44.7, 66.5)	8.5 (4.2, 12.9)
Iraq	23.1 (10.4, 35.9)	60.6 (48.8, 72.5)	16.3 (7.7, 24.8)	20.5 (17.6, 23.4)	69.0 (65.2, 72.8)	10.5 (8.2, 12.8)	26.5 (18.1, 34.9)	64.1 (55.5, 72.7)	9.4 (4.3, 14.5)	23.5 (13.7, 33.4)	69.2 (58.2, 80.1)	7.3 (2.5, 12.1)
Jordan	23.5 (11.4, 35.7)	65.7 (49.7, 81.8)	10.7 (0.0, 21.5)	19.9 (16.8, 23.1)	73.4 (69.7, 77.1)	6.6 (4.6, 8.7)	27.6 (15.9, 39.3)	65.0 (52.7, 77.4)	7.3 (1.8, 12.9)	32.0 (17.2, 46.7)	60.0 (48.8, 71.1)	8.1 (1.0, 15.1)

Kuwait	13.9 (4.6, 23.2)	83.9 (73.8, 94.0)	2.2 (0, 5.3)	14.1 (9.4, 18.7)	80.0 (74.8, 85.3)	5.9 (4.2, 7.6)	15.8 (9.7, 21.9)	78.9 (72.8, 85.1)	5.3 (1.2, 9.4)	16.1 (5.3, 26.9)	76.7 (66.2, 87.2)	7.2 (6.1, 8.3)
Lebanon	10.9 (0.0, 23.3)	77.4 (59.9, 94.9)	11.8 (0.0, 26.1)	7.7 (6.0, 9.5)	86.6 (84.1, 89.1)	5.7 (3.5, 7.9)	12.6 (7.9, 17.3)	82.2 (76.0, 88.4)	5.2 (1.2, 9.2)	16.8 (9.1, 24.5)	73.2 (53.6, 92.8)	10.0 (0.0, 23.9)
Morocco	31.2 (20, 42.3)	57.1 (46.7, 67.4)	11.8 (6.0, 17.6)	32.1 (26.6, 37.6)	54.1 (49.3, 58.8)	13.8 (11.2, 16.5)	23.1 (16.1, 30.1)	64.1 (56.6, 71.6)	12.8 (9.1, 16.4)	34.4 (20.8, 48.1)	53.8 (39.7, 68.0)	11.7 (5.5, 18.0)
Oman	17.6 (12.6, 22.5)	72.7 (67.2, 78.2)	9.8 (7.1, 12.4)	15.7 (10.8, 20.6)	77.2 (72.3, 82.0)	7.1 (5.6, 8.6)	13.9 (8.2, 19.5)	75.5 (67.3, 83.8)	10.6 (5.3, 16.0)	9.4 (2.3, 16.6)	82.8 (73.4, 92.2)	7.8 (4.4, 11.1)
Pakistan	16.0 (10.7, 21.2)	78.4 (73.3, 83.4)	5.7 (3.0, 8.3)	16.5 (12.3, 20.8)	80.1 (76.0, 84.3)	3.3 (1.7, 4.9)	15.1 (10.9, 19.4)	78.9 (74.8, 83.0)	6.0 (1.3, 10.6)	17.9 (11.4, 24.3)	78.5 (71.1, 86.0)	3.6 (0.0, 7.2)
Qatar	12.3 (0.0, 27.8)	69.5 (53.8, 85.2)	18.2 (15.5, 20.9)	22.6 (14.4, 30.8)	61.1 (54.1, 68.0)	16.4 (12.1, 20.6)	14.9 (6.0, 23.7)	72.3 (62.8, 81.8)	12.8 (4.8, 20.9)	15.2 (4.8, 25.5)	82.5 (72.0, 93.0)	2.3 (1.9, 2.8)
Sudan	4.3 (2.3, 6.2)	78.4 (70.0, 86.8)	17.3 (7.5, 27.2)	9.8 (6.9, 12.6)	77.7 (73.8, 81.6)	12.6 (9.0, 16.1)	13.2 (3.3, 23.2)	76.4 (67.4, 85.4)	10.3 (3.2, 17.5)	7.8 (0.0, 16.2)	82.1 (68.9, 95.3)	10.1 (4.4, 15.8)
Syrian Arab Republic	34.2 (25.5, 42.9)	58.0 (48.7, 67.3)	7.8 (2.8, 12.8)	33.2 (30.0, 36.5)	62.2 (58.8, 65.5)	4.6 (3.5, 5.7)	35.5 (27.2, 43.9)	60.4 (52.2, 68.7)	4.0 (1.9, 6.2)	34.4 (27.6, 41.2)	60.3 (52.7, 68.0)	5.2 (1.4, 9.1)
Tunisia	14.1 (3.7, 24.5)	74.8 (62.8, 86.9)	11.1 (5.6, 16.6)	10.3 (8.6, 11.9)	82.4 (79.4, 85.4)	7.3 (5.5, 9.2)	18.2 (13.7, 22.6)	71.4 (66.1, 76.7)	10.4 (5.5, 15.3)	14.7 (4.2, 25.2)	80.2 (68.6, 91.7)	5.2 (1.3, 9.0)
United Arab Emirates	17.9 (8.4, 27.4)	66.9 (51.9, 81.8)	15.2 (4.9, 25.6)	13.7 (10, 17.4)	77.4 (73.0, 81.8)	8.9 (7.0, 10.8)	20.0 (12.3, 27.6)	72.0 (65.3, 78.8)	8.0 (4.2, 11.8)	10.3 (3.6, 17.1)	79.1 (70.1, 88.1)	10.5 (3.6, 17.5)
UNRWA	31.3 (23.9, 38.6)	60.4 (52.9, 68.0)	8.3 (3.7, 12.9)	29.9 (27.5, 32.2)	62.5 (60.4, 64.7)	7.6 (6.5, 8.6)	30.4 (25.8, 35.0)	62.6 (58.1, 67.2)	7.0 (4.7, 9.2)	30.3 (24.0, 36.7)	58.9 (53.3, 64.4)	10.8 (6.6, 15.1)
Yemen	31.4 (14.8, 48.0)	53.8 (46.8, 60.9)	14.7 (2.0, 27.5)	35.6 (26.9, 44.4)	54.0 (46.2, 61.7)	10.4 (7.0, 13.8)	35.6 (24.8, 46.3)	55.7 (42.7, 68.8)	8.7 (1.8, 15.7)	32.3 (9.4, 55.3)	67.7 (44.7, 90.6)	–
**Pooled estimates**	**20.6 (14.4, 26.8)**	**66.0 (60.5, 71.6)**	**11.7 (8.6, 14.8)**	**20.7 (16.1, 25.3)**	**68.9 (63.6, 74.3)**	**10.0 (7.8, 12.3)**	**21.3 (17.3, 25.3)**	**68.6 (64.4, 72.8)**	**9.0 (7.0, 10.9)**	**20.2 (14.5, 26.0)**	**69.9 (64.5, 75.2)**	**8.0 (5.8, 10.2)**
**I^2 (%)**	**92.2**	**82.4**	**83**	**97.1**	**97.1**	**95.6**	**83.2**	**82.6**	**70.1**	**87.3**	**76.3**	**89**
**Southeast Asia Region**												
Bangladesh	22.8 (7.7, 37.9)	76.8 (61.7, 91.8)	0.4 (0.0, 1.3)	12.5 (10.3, 14.6)	86.6 (84.3, 88.9)	0.9 (0.1, 1.8)	5.3 (2.4, 8.2)	94.4 (91.5, 97.4)	0.2 (0.0, 0.7)	13.6 (5.8, 21.4)	84.4 (76.2, 92.6)	2.0 (0.9, 3.1)
India	4.0 (1.5, 6.4)	94.5 (91.5, 97.5)	1.6 (0.0, 3.3)	4.4 (3.4, 5.4)	93.9 (92.6, 95.3)	1.6 (1.1, 2.2)	3.7 (1.6, 5.8)	95.3 (93, 97.7)	0.9 (0.1, 1.8)	4.2 (2.6, 5.7)	94.2 (91.5, 96.9)	1.7 (0.2, 3.1)
Indonesia	1.1 (0.3, 1.9)	97.2 (95.8, 98.6)	1.7 (0.4, 3.0)	2.5 (1.8, 3.1)	93.8 (92.6, 95.0)	3.7 (2.9, 4.5)	1.7 (0.8, 2.6)	96.1 (94.9, 97.3)	2.2 (1.0, 3.4)	2.3 (0.5, 4.2)	95.8 (93.9, 97.6)	1.9 (0.2, 3.6)
Thailand	2.4 (0.0, 5.8)	88.4 (81.1, 95.6)	9.2 (3.2, 15.2)	5.2 (4.0, 6.3)	91.3 (89.4, 93.2)	3.6 (2.5, 4.7)	7.1 (2.8, 11.3)	84.6 (78.5, 90.7)	8.3 (3.4, 13.1)	6.1 (1.1, 11.0)	89.6 (82.7, 96.5)	4.4 (1.0, 7.7)
Timor-Leste	16.0 (4.8, 27.2)	77.2 (65.4, 88.9)	4.4 (1.0, 7.7)	16.8 (13.2, 20.4)	77.6 (74.0, 81.2)	5.7 (4.4, 6.9)	19.0 (13.8, 24.2)	77.8 (72.0, 83.6)	3.2 (0.0, 6.7)	12.9 (0.0, 27.3)	72.6 (57.5, 87.7)	14.5 (7.5, 21.4)
**Pooled**	**4.1 (0.9,**	**90.5 (85.3,**	**2.0 (0.5,**	**7.8 (4.7,**	**88.9 (85.0,**	**3.0 (1.6,**	**6.7 (2.8,**	**90.6 (86.2,**	**1.7 (0.4,**	**5.0 (2.4,**	**91.0 (86.7,**	**2.9 (1.2,**

**estimates**	**7.3)**	**95.7)**	**3.6)**	**11.0)**	**92.9)**	**4.5)**	**10.6)**	**95.0)**	**3.0)**	**7.7)**	**95.3)**	**4.6)**
**I^2 (%)**	**79.4**	**83.4**	**74.9**	**97.0**	**96**	**93.5**	**92.1**	**91.7**	**83.2**	**64.8**	**77.5**	**72.2**
**Western Pacific Region**												
Brunei Darussalam	3.6 (0.0, 7.3)	89.6 (82.9, 96.2)	6.8 (1.2, 12.5)	2.1 (1.3, 2.9)	88.0 (86.2, 89.7)	10.0 (8.4, 11.6)	3.4 (1.1, 5.7)	85.7 (80.8, 90.6)	10.9 (6.5, 15.3)	0.7 (0.0, 2.2)	86.4 (78.6, 94.1)	12.9 (5.3, 20.5)
Cambodia	1.5 (0.0, 4.7)	98.5 (95.3, 100.0)	–	4.9 (3.4, 6.4)	93.5 (91.9, 95.2)	1.6 (0.8, 2.3)	4.7 (0.4, 9.1)	94.7 (90.1, 99.3)	0.6 (0.0, 1.4)	2.2 (0.0, 6.9)	96.1 (91.1, 100.0)	1.8 (0.2, 3.3)
China	3.6 (1, 6.2)	94.4 (91.5, 97.3)	2.1 (0.5, 3.6)	2.3 (1.8, 2.8)	94.5 (93.8, 95.2)	3.2 (2.5, 3.8)	3.3 (1.4, 5.2)	94.5 (92.2, 96.7)	2.2 (1.4, 3.0)	4.3 (0.2, 8.4)	90.5 (86.3, 94.8)	5.1 (2.3, 8.0)
Cook	7.5 (5.2, 9.8)	85.5 (64.5, 100.0)	7.0 (0.0, 27.1)	12.3 (7.7, 16.9)	77.7 (73.5, 81.9)	10.0 (7.2, 12.8)	18.4 (0.0, 38.8)	73.7 (49.8, 97.7)	7.8 (0.0, 20.2)	9.9 (0.0, 20.9)	81.0 (66.3, 95.7)	9.1 (0.0, 25.7)
Kiribati	20.8 (9.3, 32.3)	50.4 (36.1, 64.6)	28.8 (15.0, 42.6)	20.1 (16.0, 24.2)	67.2 (62.3, 72.0)	12.7 (10.1, 15.4)	16.0 (6.2, 25.8)	64.9 (54.0, 75.9)	19.1 (9.7, 28.5)	16.8 (9.2, 24.4)	71.1 (56.8, 85.4)	12.1 (3.8, 20.4)
Laos	1.0 (0.0, 3.1)	99.0 (96.9, 100.0)	–	1.9 (0.7, 3.1)	94.1 (92.7, 95.5)	4.0 (2.6, 5.4)	3.8 (0.3, 7.2)	95.6 (92.7, 98.6)	0.6 (0.0, 1.9)	2.3 (0.0, 5.8)	94.4 (87.7, 100.0)	3.3 (0.0, 8.0)
Malaysia	3.4 (1.9, 5.0)	84.4 (81.6, 87.2)	12.2 (9.7, 14.6)	2.6 (2.1, 3.0)	86.4 (85.2, 87.6)	11.1 (10.0, 12.1)	3.7 (2.3, 5.0)	83.8 (81.5, 86.1)	12.5 (10.2, 14.8)	4.2 (2.5, 5.9)	85.6 (82.7, 88.5)	10.2 (7.8, 12.6)
Mongolia	6.5 (2.6, 10.4)	76.9 (71.1, 82.7)	16.6 (9.6, 23.6)	5.5 (4.5, 6.5)	84.1 (82.6, 85.7)	10.4 (9.0, 11.7)	4.6 (2.4, 6.8)	83.5 (79.1, 87.9)	11.9 (7.7, 16.1)	5.8 (2.2, 9.3)	86.2 (81.2, 91.3)	8.0 (4.0, 11.9)
Nauru	26.1 (0.0, 56.8)	30.9 (0.0, 62.6)	43.0 (6.9, 79.2)	11.7 (7.6, 15.9)	55.4 (49.1, 61.7)	32.8 (26.8, 38.8)	13.0 (0.3, 25.7)	51.2 (32.6, 69.9)	35.8 (18, 53.6)	15.8 (0.0, 32.2)	26.3 (6.5, 46.1)	57.9 (35.7, 80.1)
Philippines	3.9 (1.0, 6.8)	87.3 (81.0, 93.6)	8.8 (3.4, 14.2)	3.6 (2.7, 4.5)	87.6 (86.0, 89.1)	8.8 (7.7, 10.0)	4.0 (1.9, 6.0)	85.8 (80.5, 91.2)	10.2 (5.1, 15.4)	1.9 (0.2, 3.6)	91.5 (88.8, 94.3)	6.6 (4.5, 8.6)
Samoa	22.6 (8.2, 37.1)	59.3 (54.2, 64.5)	18.0 (4.9, 31.1)	15.7 (11.9, 19.5)	58.4 (53.6, 63.2)	25.9 (21.5, 30.4)	16.0 (9.2, 22.8)	62.1 (53.7, 70.4)	21.9 (15.8, 28.0)	15.1 (6.9, 23.3)	66.4 (58.1, 74.6)	18.6 (10.9, 26.2)
Solomon	26.8 (0.0, 55.5)	63.8 (45.3, 82.3)	9.4 (0.0, 22.6)	23.7 (19.1, 28.4)	57.6 (50.5, 64.7)	18.7 (12.2, 25.1)	22.7 (14.8, 30.6)	51.3 (37.8, 64.7)	26.0 (9.9, 42.2)	8.4 (0.2, 16.6)	71.2 (46.6, 95.7)	20.4 (0.0, 44.7)
Tonga	10.9 (3.7, 18.1)	66.3 (55.3, 77.3)	22.8 (14.6, 31.1)	9.0 (7.1, 10.9)	68.6 (64.9, 72.3)	22.4 (19.1, 25.7)	7.2 (3.6, 10.8)	70.2 (63.7, 76.7)	22.6 (16.1, 29.1)	12.5 (6.6, 18.3)	66.3 (58.5, 74.2)	21.2 (14.0, 28.4)
Tuvalu	28.3 (6.5, 50.1)	57.0 (31.6, 82.4)	14.7 (0.0, 36.2)	7.9 (5.1, 10.8)	55.6 (50.4, 60.7)	36.5 (31.5, 41.5)	2.1 (0.0, 6.2)	59.7 (44.9, 74.5)	38.2 (23.5, 52.9)	10.7 (0, 22.9)	56.2 (36.2, 76.3)	33.1 (14.0, 52.2)
Vanuatu	19.5 (0.0, 41.5)	61.8 (20.4, 100.0)	18.7 (0.0, 38.8)	18.7 (14.7, 22.6)	61.3 (52.7, 69.8)	20.1 (13.3, 26.9)	15.7 (1.9, 29.5)	61.9 (42.7, 81.2)	22.4 (13.4, 31.4)	17.5 (0.0, 44.7)	59.5 (34.4, 84.6)	23.0 (18.4, 27.7)
Vietnam	2.5 (1.7, 3.4)	95.0 (93.5, 96.5)	2.5 (1.8, 3.2)	3.6 (2.5, 4.7)	95.5 (94.3, 96.6)	0.9 (0.5, 1.3)	1.1 (0.0, 2.9)	97.5 (95.6, 99.3)	1.4 (1.1, 1.7)	5.6 (2.5, 8.8)	93.4 (88.1, 98.7)	1.0 (0.0, 3.4)
Wallis and Futuna	13.3 (0.0, 29.6)	69.4 (45.0, 93.9)	17.3 (0.0, 35.4)	8.9 (6.3, 11.5)	75.4 (71.0, 79.8)	15.7 (11.5, 19.9)	12.0 (4.1, 19.9)	65.6 (55.0, 76.2)	22.4 (12.3, 32.4)	9.2 (0.0, 20.1)	74.6 (56.1, 93.1)	16.2 (3.7, 28.7)
**Pooled estimates**	**4.9 (3.2, 6.5)**	**80.0 (74.6, 85.4)**	**11.3 (7.8, 14.8)**	**7.9 (6.5, 9.4)**	**77.3 (73.3, 81.4)**	**13.6 (10.8, 16.3)**	**5.7 (4.0, 7.4)**	**78.1 (73.0, 83.3)**	**10.0 (8.0, 12.1)**	**5.7 (3.8, 7.6)**	**80.4 (75.5, 85.4)**	**11.5 (8.1, 14.9)**

**I^2 (%)**	**74.9**	**96.1**	**89.1**	**95.9**	**98.7**	**98.7**	**77.3**	**95.2**	**95.2**	**76.3**	**89.3**	**91.6**
**Total**												
**Pooled estimates**	**7.3 (4.2, 10.4)**	**80.1 (72.4, 87.9)**	**9.4 (4.3, 14.4)**	**8.3 (5.1, 11.4)**	**81.9 (75.7, 88.2)**	**9.1 (5.5, 12.6)**	**7.8 (4.1, 11.4)**	**82.4 (74.9, 89.9)**	**7.7 (3.8, 11.6)**	**6.2 (3.2, 9.3)**	**82.7 (76.4, 88.9)**	**8.5 (5.1, 11.9)**
**I^2 (%)**	**88.0**	**90.8**	**93.1**	**93.3**	**95.3**	**93.2**	**93.1**	**95.1**	**93.5**	**89.9**	**88.9**	**86.3**
